# Reductive Transformation of Imine Covalent Organic Frameworks into Emissive Polymers: Insights into Emission Quenching

**DOI:** 10.1021/acs.chemmater.5c01951

**Published:** 2025-10-01

**Authors:** Agata Tyszka-Gumkowska, Mateusz Brzeziński, Sylwester Gawinkowski, Tomasz Polczyk, Wojciech Wegner, Mateusz Wlazło, Piotr Bernatowicz, Jan Nawrocki, Przemysław Gaweł, Krzysztof Noworyta, Jakub Ostapko

**Affiliations:** † 656563 Centre of Excellence ENSEMBLE3 Sp. zo.o., Wólczyńska 133, 01-919 Warsaw, Poland; ‡ 119463 Institute of Physical Chemistry, Polish Academy of Sciences, Marcina Kasprzaka 44/52, 01-224 Warsaw, Poland; § Faculty of Chemistry, 37799 Jagiellonian University, Gołębia 24, 31-007 Kraków, Poland; d Center of New Technologies, University of Warsaw, Stefana Banacha 2c, 02-097 Warsaw, Poland; e Institute of Organic Chemistry, Polish Academy of Sciences, Marcina Kasprzaka 44/52, 01-224 Warsaw, Poland

## Abstract

Although imine-linked covalent organic frameworks (COFs) are readily synthesized, their lack of intrinsic emission constrains their potential in optoelectronic applications. Moreover, the underlying mechanisms governing excited-state energy dissipation remain poorly understood. To overcome these limitations, we present a straightforward strategy to transform nonemissive, anthracene-based imine-COFs (**im-COF**) into highly emissive amine-based covalent organic polymers (**am-COP**) through NaBH_3_CN/benzoic acid reduction, achieving solid-state emission quantum yields of up to 30%. To clarify the excited-state energy dissipation mechanisms in COFs and COPs, we synthesized molecular reference compounds featuring either imine or amine functionalities. Photophysical studies, supported by density functional theory (DFT) calculations, reveal that the excited states of both amines and imines exhibit weak charge transfer (CT) properties. Furthermore, a comparative analysis of the photophysical behavior of molecular references, **im-COFs**, and **am-COPs** demonstrates that the polymer emission arising from charge-transfer processes is modulated by the polymer’s intrinsic polarity. These findings provide fundamental insights into the photophysical behavior of COFs, thus paving the way for the development of emissive porous materials with promising advanced applications.

## Graphical abstract

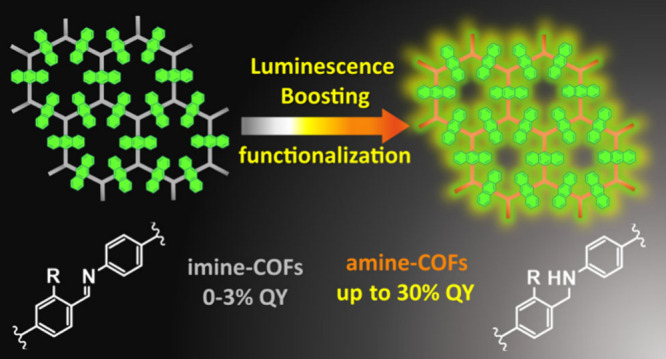

## Introduction

Combining the functional versatility of organic molecules with the structural benefits of crystalline porous materials, covalent organic frameworks (COFs) have attracted significant attention since 2005, following the first report of this novel class of crystalline porous polymers by Yaghi.^1^ The unique interplay between crystal topology and physicochemical properties has enabled COFs to be integrated into diverse scientific disciplines, such as catalysis,^2,3^ gas sorption,^4–7^ chemical separation,^8^ and sensing,^9^ along with their potential applications in prominent areas such as optoelectronics^10,11^ and energy storage.^12–14^

Forming a reticular framework of COFs relies on the chemical compatibility of specifically chosen functional groups of the building blocks. Among the various reported approaches, the condensation of amine and carbonyl groups, which results in the formation of imine-COFs, is one of the most widely utilized.^15,16^ This strategy benefits from the accessibility of building blocks, the relatively mild conditions of solvothermal synthesis, and the reversibility of imine bond formation, which facilitates the “self-healing” of structural defects.^17^ Consequently, imine-COFs are readily available and typically exhibit high crystallinity and stability. Additionally, the presence of the imine group allows for further postsynthetic functionalization of the framework.^18–25^

The imine-COFs share key properties with other COFs, including a tunable architecture and broad applicability. However, they differ significantly in their optical behavior, as imine-COFs often exhibit very weak or no fluorescence, even when they are derived from intrinsically luminescent building blocks. In the literature, it is commonly attributed to the aggregation-caused quenching (ACQ) and nonradiative decays associated with intramolecular rotations.^26–37^ Additionally, strong π–π interactions between adjacent stacked layers in the 2D imine-COF structure can lead to charge separation, resulting in dissipation and nonradiative recombination of the excited state. Despite the widespread acceptance of these explanations, the quenching phenomena in COFs remain largely unexplored, highlighting a critical gap in understanding their photophysical behavior.

Several strategies have been reported to overcome the emission quenching problem in imine-COFs, and they can be broadly categorized into two groups. The first focuses on modulating through-space interactions between aromatic emitters by controlling π–π stacking, thereby reducing nonradiative deactivation. This is achieved through fine-tuning the stacking mode,^26^ introducing bulky aromatic linkers,^38^ limiting both intralayer conjugation and interlayer π–π stacking by incorporating aliphatic linkers,^28^ and employing emissive units prone to aggregation-enhanced emission.^27,38^ The second group involves modification of the intrinsic properties of the emitters. Examples include the introduction of push–pull systems,^31–33,39^ some of which exhibit dual emission due to intramolecular charge transfer^40,41^ or excited-state intramolecular proton transfer.^42^ Another strategy involves COFs incorporating fluorene-derived linker units, from which the emission originates.^43^ Additionally, studies have demonstrated the isotope effect of H–D substitution as a tool for fine-tuning vibrational states to enhance emission.^34^ Considering all mentioned strategies, the highest reported quantum yields were achieved by Peng et al., who employed a flexible aromatic vortex (43.5%),^35^ and by Müllen et al., who utilized an aliphatic linker for the reticular interconnection of emitters (57%).^28^

To date, all reported strategies for constructing highly emissive COFs have relied on specially designed frameworks that align with one of the strategies listed above. Consequently, stringent structural requirements significantly limit the range of accessible emitters and frameworks for functional COF construction, thereby restricting their potential applications.

In this study, we present a postsynthetic method that transforms nonemissive imine-COFs into highly emissive amine analogues. To demonstrate this approach, we synthesized two imine-based polymers, amorphous **im-COP-1** and crystalline **im-COF-2**, both incorporating a prototypical anthracene emitter unit and exhibiting nonemissive or weakly emissive properties. A straightforward reduction of **im-COP-1** and **im-COF-2** using NaBH_3_CN yielded highly emissive amine-based polymers, **am-COP-3** and **am-COP-4**, with solid-state emission quantum yields of 30% and 17%, respectively. Furthermore, we compared the photophysical properties of the imine- and amine-based polymers with those of their molecular reference compounds, denoted here as **im-ref** and **am-ref**, respectively. This comparison, combined with DFT calculations, provides valuable insight into the mechanisms underlying excited-state energy dissipation in imine-based COF­(P)­s and explains the emission behavior of their amine-derived analogues.

## Experimental Section

### Synthesis of **im-COP-1**

An 8 mL vial was filled with TFPB (19.52 mg, 0.05 mmol) and BAPA (27.03 mg, 0.15 mmol). The vial was then transferred to the glovebox, and solvents (0.8 mL of 1,2-dichlorobenzene and 0.2 mL of *n*-butanol) were added, followed by the addition of 6 M AcOH_aq_ (50 μL). The vial was capped and placed in a heating block set at 150 °C, where it was left undisturbed for 7 days. After this period, the yellow solid that formed was filtered off and washed extensively with CH_2_Cl_2_. The solid was further purified by Soxhlet extraction with THF overnight. After extraction, the solid was dried under vacuum for at least 5 h at 120 °C, yielding the desired compound as a yellow solid (40.6 mg, 89%). Elemental analysis calcd for C_132_H_84_N_6_: C, 90.40; H, 4.79; N, 4.79. Found: C, 88.24; H, 4.86; N, 4.40.

### Synthesis of **im-COF-2**

An 8 mL vial was filled with TFHPB (21.91 mg, 0.05 mmol) and BAPA (27.03 mg, 0.15 mmol). The vial was then transferred to the glovebox, and 1.9 mL of 1,2-dichlorobenzene and 0.1 mL of *n*-butanol were added, followed by the addition of 6 M AcOH_aq_ (100 μL). The vial was capped and transferred to a heating block set at 120 °C and left undisturbed for 5 days. After this time, the yellow solid that formed was filtered off and washed extensively with CH_2_Cl_2_. The solid was further purified by Soxhlet extraction with THF overnight. After extraction, the solid was dried under vacuum for at least 5 h at 120 °C to yield the desired compound as a yellow solid (39 mg, 85%). Elemental analysis calcd for C_132_H_84_N_6_O_6_: C, 85.71; H, 4.45; N, 4.45. Found: C, 81.58; H, 4.36; N, 4.21.

### Synthesis of **am-COP-3**

**im-COF-1** (98 mg, 0.056 mmol) was placed in a vial along with benzoic acid (54 mg, 0.45 mmol) and sodium cyanoborohydride (NaBH_3_CN, 141 mg, 2.24 mmol). Then, 10.8 mL of tetrahydrofuran (THF) and 3.2 mL of ethanol (EtOH) were added, causing gas evolution. The reaction mixture was left in an open vial for at least 30 min before being sealed and left to react overnight. After this, the solid was collected by filtration and washed with methanol (MeOH). The solid was further purified by Soxhlet extraction with MeOH overnight. After extraction, the solid was vacuum-dried at 120 °C for at least 5 h, yielding the desired compound as a yellow solid (94 mg, 96%). Elemental analysis calcd for C_132_H_96_N_6_: C, 89.80; H, 5.44; N, 4.76. Found: C, 88.46; H, 4.42; N, 5.05.

### Synthesis of **am-COP-4**

The material was synthesized in an analogous manner, as **am-COP-3** using **im-COF-2** (100 mg, 0.055 mmol) as a starting material, yielding the desired compound as a yellow solid (88 mg, 88%). Elemental analysis calcd for C_132_H_96_N_6_O_6_: C, 85.16; H, 5.16; N, 4.52. Found: C, 79.39; H, 5.33; N, 3.91.

## Results and Discussion

### Design and Synthesis

The guiding principle of the COF­(P) structure design was to incorporate the prototypical emitter, anthracene, into the lattice structure while concurrently circumventing enol-imine to keto-amine tautomerization, as observed in triformylphloroglucinol-derived COFs.^44^ Preventing tautomerization simplifies the analysis of the physicochemical properties and enables comparative studies between the synthesized COFs and structurally similar molecular reference compounds.

Therefore, the designed imine-COFs consist of 9,10-diphenylanthracene units as linkers and vortexes derived from 1,3,5-triphenylbenzene (Figure 1). These building blocks are connected via imine bonds, with a nitrogen atom bonded to the side phenyl group on diphenylanthracene. Proposed structures may be constructed using known COF’s building blocks: 9,10-bis­(4-aminophenyl)­anthracene (BAPA),^45–47^ 1,3,5-tris­(4-formylphenyl)­benzene (TFPB),^48^ and 1,3,5-tris­(4-formyl-3-hydroxyphenyl)­benzene (TFHPB)^49^ by aldehyde–imine condensation (Figure 1). Each designed polymer has an associated molecular reference, where the 1,3,5-triphenylbenzene core is replaced with monofunctional di-*tert*-butylbenzene motifs to provide better solubility in organic solvents (Figure 2).

**Figure 1:**
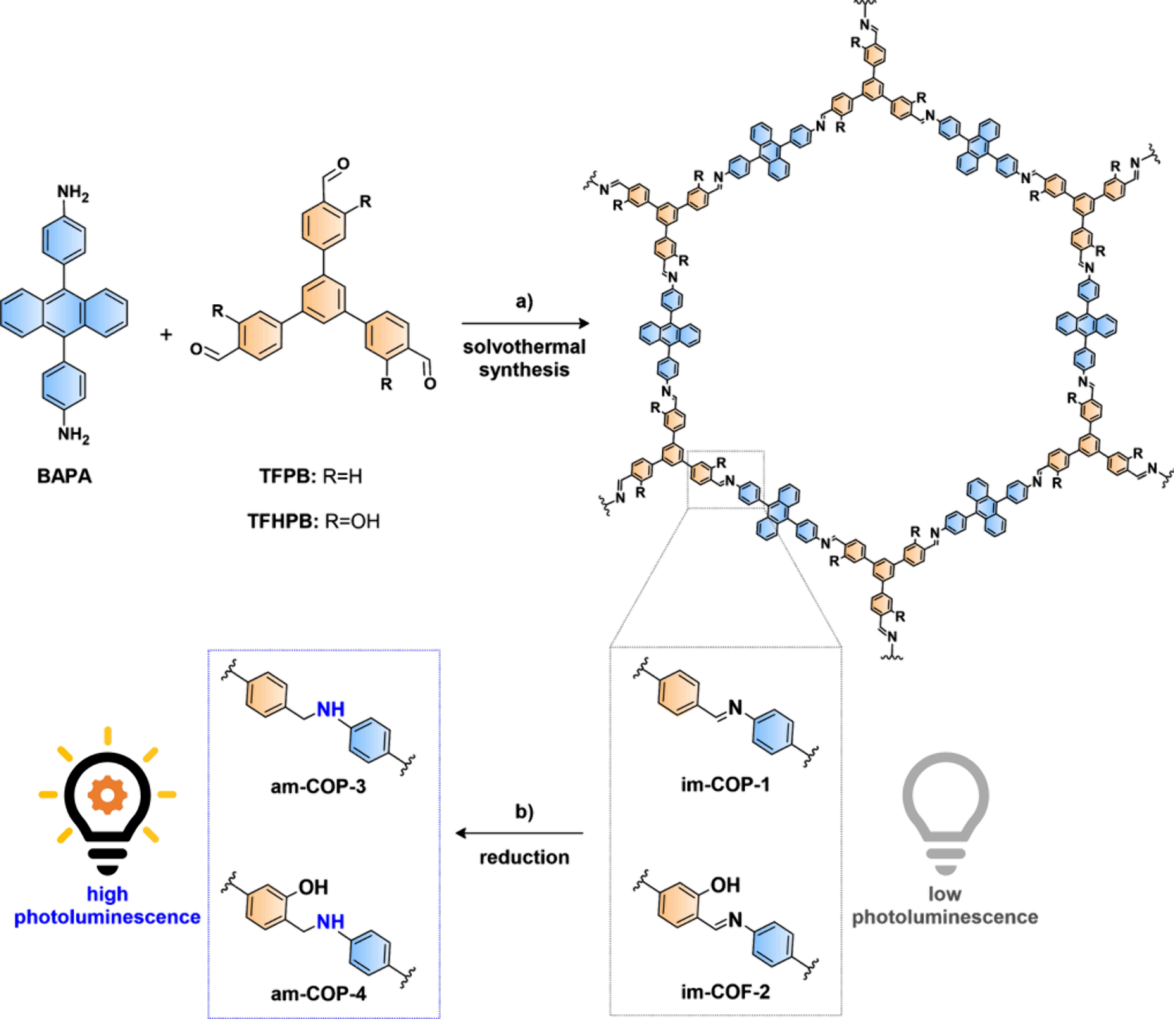
Solvothermal synthesis of imine-COF­(P)­s and their reduction into amine-COFs. (a) For **im-COP-1**: 6 M AcOH, ODCB/*n*-BuOH (8:2, v/v), 150 °C, 7 days, 89% yield. For **im-COF-2**: 6 M AcOH, ODCB/*n*-BuOH (19:1, v/v), 120 °C, 5 days, 85% yield. (b) NaBH_3_CN/BzOH, THF/EtOH (3:1, v/v), RT, 16 h; 96% yield of **am-COP-3**, 88% yield of **am-COP-4**.

**Figure 2:**
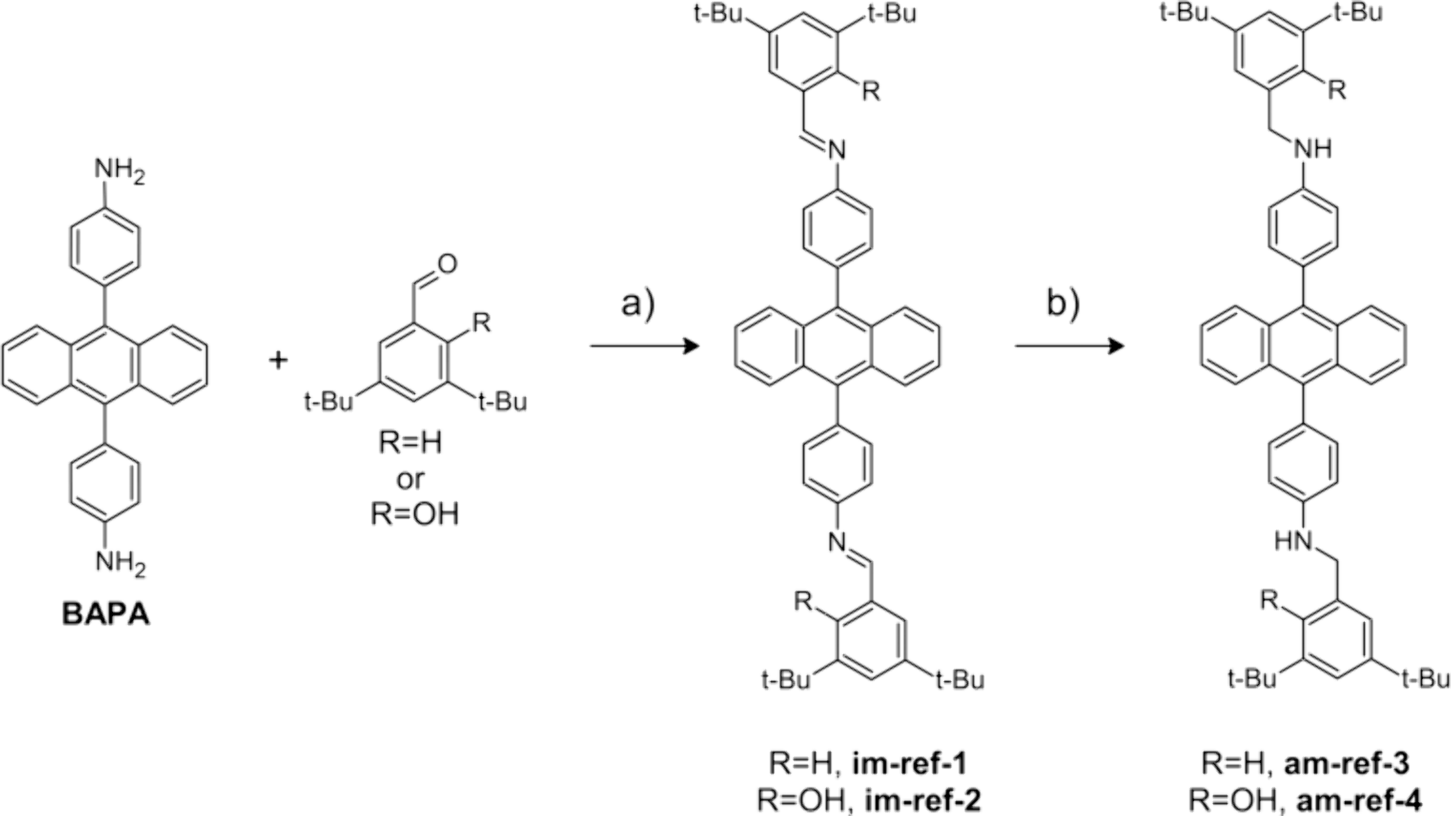
Synthesis of reference imines and their reduction into reference amines. (a) DMSO, 1 h, 100 °C, 95% yield of **im-ref-1**, 95% yield of **im-ref-2**. (b) NaBH_4_, THF/EtOH (3:1, v/v), RT, 2 h, 95% yield of **am-ref-3**, 95% yield of **am-ref-4**.

COF­(P)­s and their molecular references were synthesized in two variants, distinguished by the presence or absence of a hydroxyl group at the position adjacent to the imine group. The hydroxyl group is expected to form an intramolecular hydrogen bond with the imine nitrogen atom, thereby restricting rotational freedom and enhancing the structural rigidity in COF­(P)­s and their references.

The synthesis of COF­(P)­s was optimized using the solvothermal method, with crystallinity monitored by powder X-ray diffraction (PXRD) (Figures S9 and S10).

**im-COF-2** was obtained as a crystalline material under the optimized solvothermal conditions, employing a solvent mixture of *o*-dichlorobenzene and *n*-butanol (19:1, v/v), a reaction temperature of 120 °C, and a reaction time of 5 days. It is noteworthy that in some experiments conducted at 150 °C, the crystallinity of the material was even higher than that observed at 120 °C. However, synthesis at higher temperatures suffered from low reproducibility and poor scalability.

Attempts to apply the previously optimized conditions for synthesizing crystalline **im-COP-1**, not possessing an intramolecular hydrogen bond, were unsuccessful. Further optimization did not improve the crystallinity (Figure S10). Consequently, **im-COP-1** was obtained in an amorphous form using a solvent mixture of *o*-dichlorobenzene and *n*-butanol (8:2, v/v) at 150 °C after reaction for 7 days. These results indicate a significant influence of the intramolecular hydrogen bond present in the COF structure on the formation of its crystalline phase due to the strengthening of the π–π interaction in the lattice.

The synthesis of **am-COP-3** and **am-COP-4**, achieved by postfunctionalization of imine-COF­(P)­s, was monitored by Raman spectroscopy during optimization (Figure S11), while the product’s crystallinity has been examined by PXRD (Figure S12). Unfortunately, we were unable transform imine into respective amine-COF maintaining its crystallinity, despite examining several different reduction conditions.^22,25,50^ Eventually, amine-COPs were synthesized by reduction with NaBH_3_CN/benzoic acid.

Reference compounds were synthesized via the direct condensation of 9,10-di­(4,4′-bisaminophenyl)­anthracene (BAPA) with aldehydes in the absence of an acidic catalyst. DMSO used as the reaction medium facilitated the straightforward isolation of **im-ref-1** and **im-ref-2** as precipitates with high yields (Figure 2a). Their subsequent reduction to amines **am-ref-1** and **am-ref-2**, respectively, was carried out using NaBH_4_ (Figure 2b and Figures S16 and S17).

The formation of the imine-COF­(P)­s, **im-COP-1** and **im-COF-2**, was confirmed by Raman spectroscopy (for IR results, see Figure S17) by the presence of C=N stretching bands at 1623 and 1628 cm^–1^, respectively (Figure 3). For molecular references, the analogous bands appear at 1632 cm^–1^ for **im-ref-1** and 1617 cm^–1^ for **im-ref-2** (Figures S13–S16 for simulated IR/Raman spectra). The subsequent disappearance of these bands suggested the successful reduction of imines to their corresponding amines in both COF­(P)­s and their molecular analogues. Additionally, spectra of **im-COP-1** and **im-COF-2** do not reveal any traces of the substrates, TFPB and TFHPB, which exhibit C=O stretching bands at 1693 and 1657 cm^–1^, respectively (Figure 3a,b, highlighted by a blue strip), indicating a conversion of the starting aldehydes and high purity of obtained polymers.

**Figure 3:**
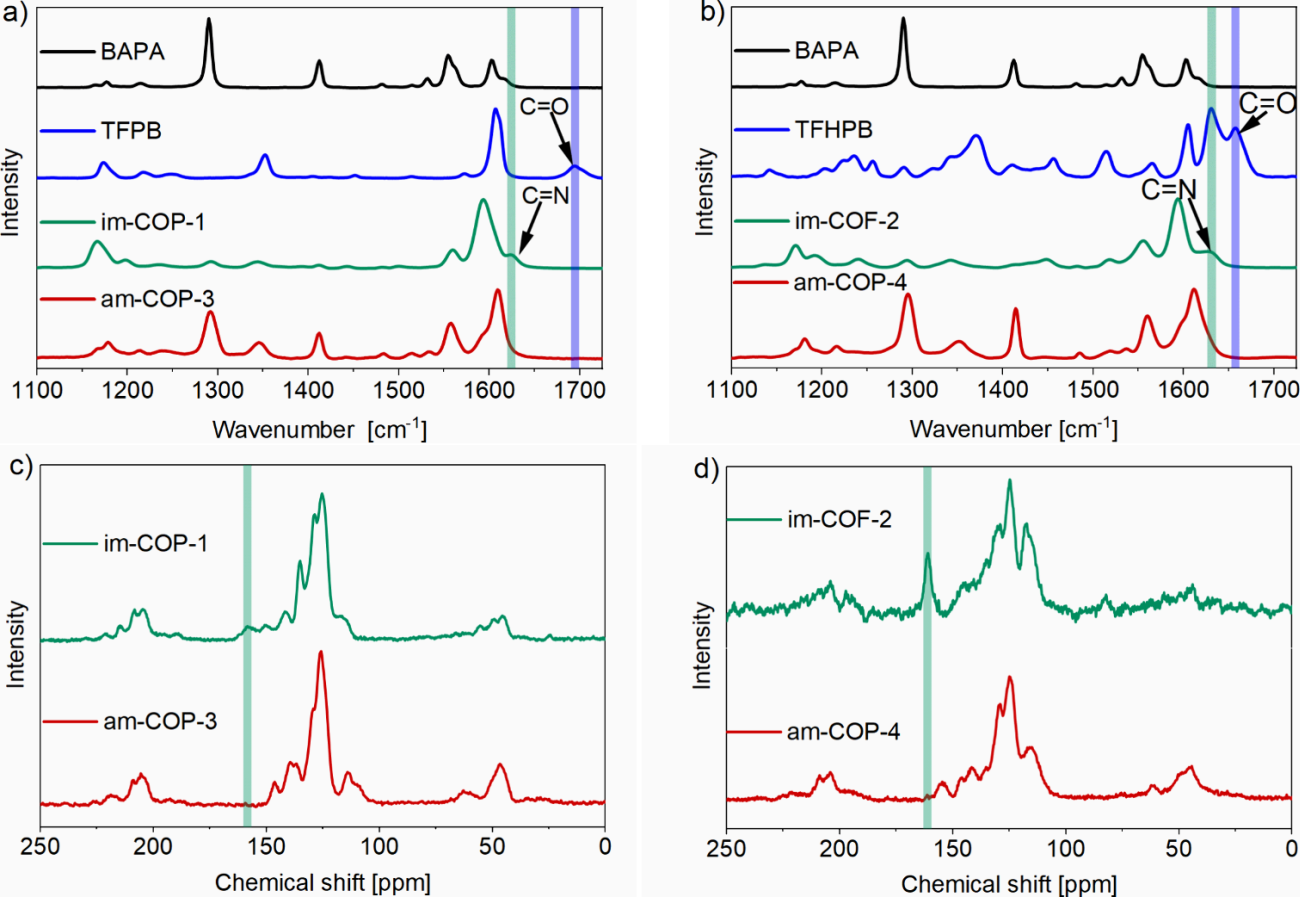
(a and b) Raman spectra of **im-COP-1** and **im-COF-2**, its reduced forms, **am-COP-3** and **am-COP-4**, and the substrates used for synthesis. (c and d) ^13^C CP-MAS NMR spectra of imine-COFs **im-COF-2** and **im-COP-1** and their reduced analogues **am-COP-4** and **am-COP-3**.

The formation of im-COF­(P)­s and their reduction to am-COPs was also confirmed by ^13^C cross-polarization magic angle spinning (CP-MAS) NMR spectroscopy. The imine carbon atoms give a characteristic signal at 158 ppm for **im-COP-1** and a slightly downfield one at 161 ppm for **im-COF-2** (Figure 3c,d, highlighted by a blue strip). These signals disappear upon reduction, confirming the quantitative transformation of imine into amine groups, which is consistent with the Raman and IR data.

### Structural Characterization

PXRD measurements of **im-COF-2** revealed a pattern consisting of several reflections appearing in the small-angle region. Structural modeling allowed us to propose a plausible structure. Using VASP software, we generated two stacking mode models: eclipsed AA and staggered AB (see Figure S9). The comparison between experimental and simulated PXRD patterns suggests the presence of the AB stacking mode in **im-COF-2**; however, the assignment is tentative due to similar cell parameters found for AA and AB stacked structures (Figure 4 and Table S5). Nevertheless, the simulated structure allowed us to assign the three visible peaks on the experimental PXRD pattern of **im-COF-2** to the following plane reflections: 2.20° (100, 110), 3.64° (120, 1–10), 4.18° (200, 220), 7.18° (2–20, 240), and 11.16° (different reflexes). Furthermore, Le Bail refinement was applied to the experimental PXRD pattern (Figure S20). The refined unit cell parameters were as follows: *a* = 50.86 Å, *b* = 52.37 Å, *c* = 7.60 Å, α = 89.04°, β = 91.67°, and γ = 63.14° with *R*_wp_ = 0.34 and *R*_p_ = 0.25.

**Figure 4:**
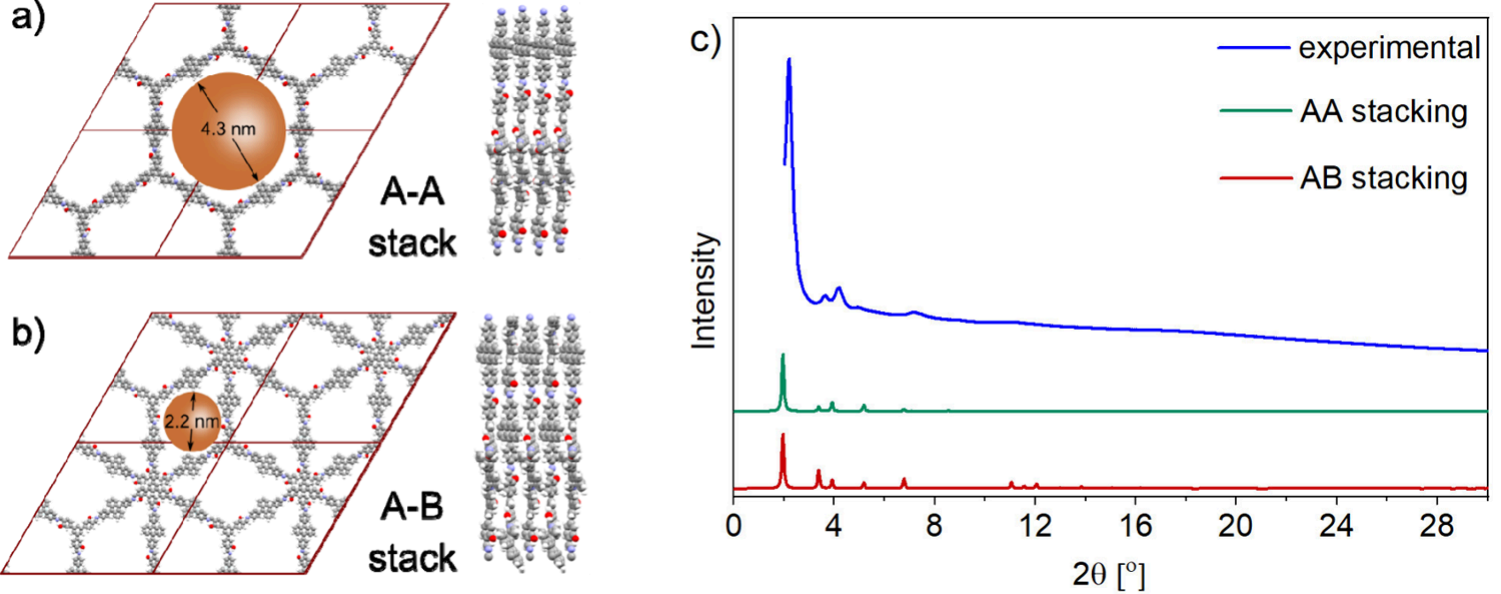
(a) AA stacking mode unit cell of **im-COF-2**. (b) PXRD pattern of **im-COF-2**. (c) Experimental pattern (blue), simulated pattern for the AA stacking mode (green), and simulated pattern for the AB stacking mode (red).

Additionally, the synthesized polymers were evaluated for surface area and pore size distribution using nitrogen adsorption–desorption isotherm analysis at 77 K. **im-COF-2** exhibits a combination of type I and IV adsorption isotherms according to the IUPAC classification,^51^ which is characteristic of the micropores and mesopores present in the material. The Brunauer–Emmett–Teller (BET) surface area of **im-COF-2** was determined to be 819 m^2^/g. The pore size distribution, calculated using nonlocal density functional theory (NLDFT), was found to be 2.4 nm (Figure 5, inset), suggesting the presence of the staggered (AB) stacking mode in **im-COF-2**, with a predicted pore size of 2.2 nm (Figure 4a).

**Figure 5:**
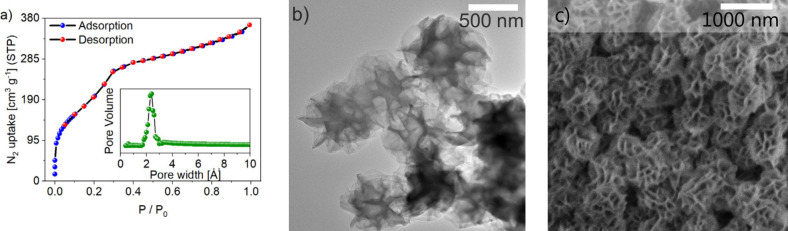
(a) N_2_ adsorption and desorption isotherms for **im-COF-2**. The inset shows the pore size distribution. (b) TEM image of **im-COF-2**. (c) SEM image of **im-COF-2**.

The reliable isotherms for **im-COP-1** and **am-COPs** could not be obtained due to their low porosities. Given the sensitivity of the sorption instrument, we conclude that the surface areas for all amorphous materials are less than 70 m^2^/g.

The morphological differences between **im-COP-1** and **im-COF-2** are reflected in the scanning electron microscopy (SEM) images (Figure S21). The **im-COP-1** sample revealed the presence of spherical grains, and this morphology is retained upon its transformation into **am-COP-3**. SEM images of the **im-COF-2** sample show flower-like structures with distinguishable discrete morphological features. Transmission electron microscopy (TEM) imaging of **im-COF-2** further reveals that these flower-like structures are composed of bent layers of organic material rather than visibly arranged crystals (Figure 5b and Figure S22). Reduction of **im-COF-2**, resulting in the formation of **am-COP-4**, does not change the sample’s morphology, despite the loss of material crystallinity as evidenced by PXRD (Figure S12), and thus, we attribute the amorphization of **im-COF-2** to **am-COP-4** to the relaxation of the reduced framework.

Both imine-COF­(P)­s, **im-COP-1** and **im-COF-2**, exhibit high thermal stability, with decomposition temperatures above 400 °C determined by thermogravimetric analysis (TGA) and differential scanning calorimetry (DSC). Their amine derivatives, **am-COP-3** and **am-COP-4**, show lower stability, decomposing at approximately 250 and 200 °C, respectively (Figure S23). For all of the studied COFs, the decomposition proceeds in two distinct exothermic steps.

### Optoelectronic and Electrochemical Properties

To gain a deeper understanding of the physicochemical properties of the synthesized polymers, we first discuss the spectroscopic characteristics and the DFT calculation results within the series of molecular references (**im-ref-1**, **im-ref-2**, **am-ref-3**, and **am-ref-4**) and then compare them with those of the corresponding COF­(P)­s.

All reference materials exhibit similar absorption spectra (Figure 6 a,c,e,g). The absorption maxima are almost unaffected by the type of chemical bond (imine or amine) or solvent polarity. This is consistent with the DFT calculations (Figure 7). For all discussed references, the mapped HOMO and LUMO orbitals of the S_0_ state are strictly localized on the anthracene core, highlighting the similar character of their vertically excited states. Additional evidence for the similarity between the ground states of the model compounds is provided by cyclic voltammetry (CV) measurements (Figure S35) since all studied compounds exhibit similar electrochemical bandgap values (2.6–2.7 eV (Table S13)), which are in good agreement with the observed optical characteristics.

**Figure 6:**
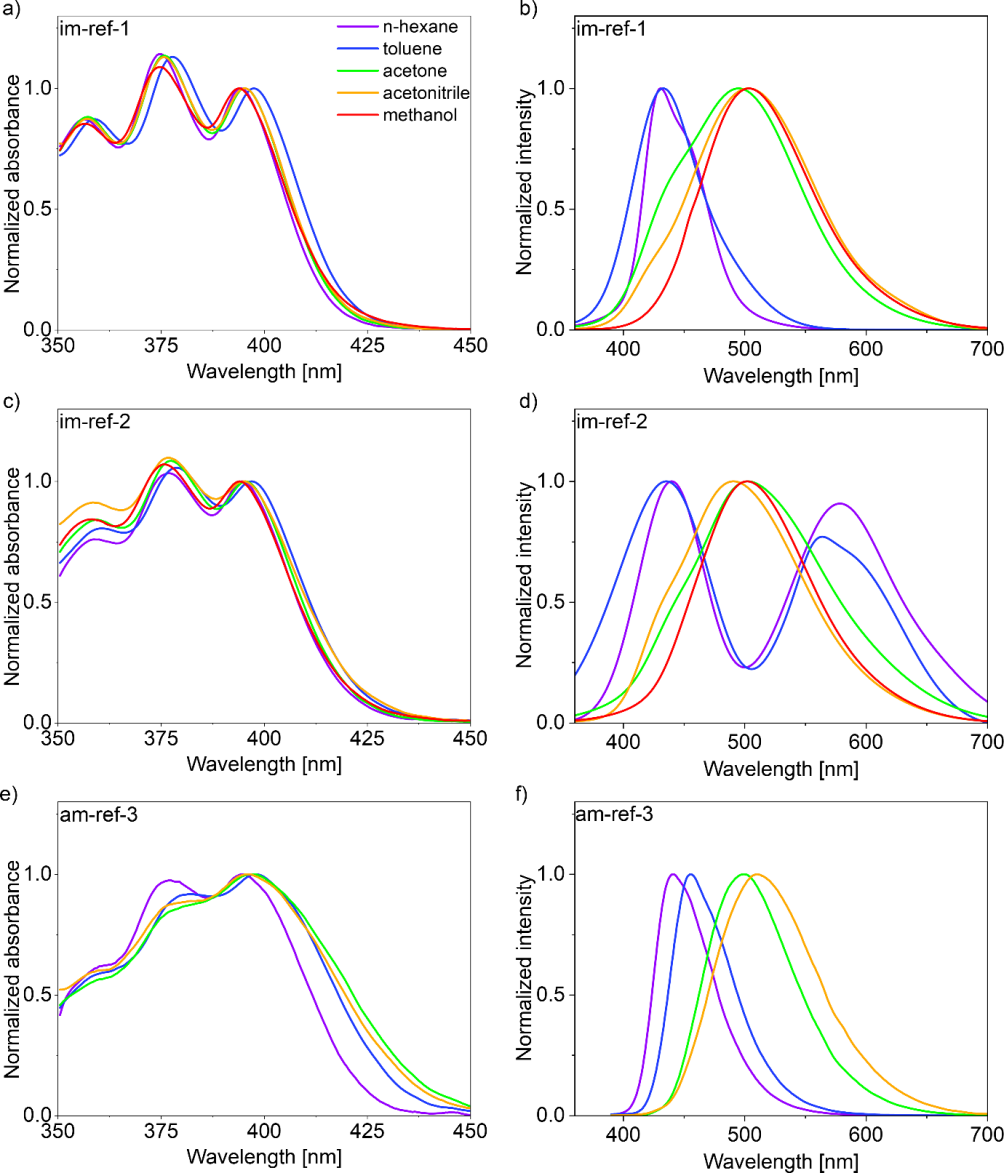
(a, c, e, and g) Absorption spectra (normalized to the third peak) and (b, d, f, and h) emission spectra (normalized globally) of reference imines (a–d) and amines (e–h) measured in different solvents (marked using global the color theme presented in panel a).

**Figure 7:**
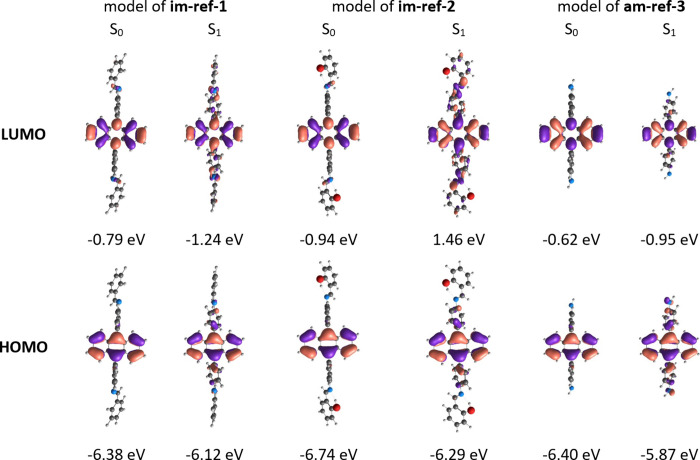
Frontier orbital distribution calculated for the relaxed ground and excited states of **im-ref-1**, **im-ref-2**, and **am-ref-3** model representatives.

Contrary to the absorption spectra, all references exhibit a gradual red-shift of the emission spectra maximum as the solvent polarity increases (Figure 6 b,d,f,h; see Figure S28 for relations to *E*_T_(30)). Additionally, the vibronic structure of the emission band vanishes with an increase in solvent polarity. Both features are reminiscent of the geometric and electronic changes in excited states. As evaluated by the DFT calculations, all compounds relax in the excited state by rotation of the phenyl substituents attached directly to the anthracene unit (Figure 7). This allows the excited-state HOMO* and LUMO* orbitals to delocalize over the phenyl substituents. Such changes in excited-state electron distribution resemble the features of excited-state charge transfer.

Among the discussed references, **im-ref-2** is the only studied compound that exhibits dual emission in nonpolar solvents. Thus, we hypothesized that the low-energy emission of **im-ref-2** originates from the aggregates. To evaluate this hypothesis, we monitored the emission spectra of **im-ref-2** as a function of its concentration. Indeed, as the sample concentration decreased, the intensity of the low-energy band also decreased (Figure S31). Consistent with this behavior, the solid-state emission of **im-ref-2** exhibits only a low-energy emission band at 560 nm (Figure S32). Additionally, excitation spectral measurements provide further evidence of aggregate formation (Figure S31). When excitation was monitored by probing the low-energy emission at 560 nm, additional maxima appeared around 415 nm in the excitation spectra, indicating that the emission originates from species other than monomeric **im-ref-2**. Again, this observation aligns with the absorbance spectra acquired for the solid-state sample (Figure S32), which show red-shifted bands compared to the solution-phase measurements. Overall, these results indicate that the low-energy emission at 560 nm arises from the presence of aggregates.

The characteristics of the reference compounds discussed above provided insight into the properties of the studied COF­(P)­s. By empirical observation, imines **im-ref-1**, **im-ref-2**, and **im-COP-1** are nonemissive while **im-COF-2** exhibits a yellow emission of moderate intensity. The situation changed significantly upon the imine-to-amine transformation. The amine derivatives (**am-ref-3**, **am-ref-4**, **am-COP-3**, and **am-COP-4**) display bright greenish fluorescence, quantitated by measured fluorescence quantum yields provided in Tables 1 and 2 (see also Table S12).

**Table 1:** Measured Quantum Yields (QY) of Reference Compound Fluorescence in Different Solvents

	QY (%)		
	toluene	acetonitrile	fluorescence lifetime (ns)
**im-ref-1**	0.21 ± 0.03	0.67 ± 0.07	–^a^
**im-ref-2**	0.16 ± 0.05	0.56 ± 0.04	–^a^
**am-ref-3**	74.82 ± 5.83	43.94 ± 2.91	2.12/5.45^b^
**am-ref-4**	75.18 ± 6.06	38.62 ± 0.68	2.25/5.97^b^

a The result is at the limit of measurement error.

b Solutions in toluene/acetonitrile.

**Table 2:** Measured QYs and Lifetimes of the Fluorescence of COF­(P)­s in a Solid State

	solid-state fluorescence QY (%)	fluorescence lifetime (ns)
**im-COP-1**	–^a^	–^b^
**im-COF-2**	3.08	–^b^
**am-COP-3**	29.76	1.5/1.8^c^
**am-COP-4**	17.10	1.8/2.3^c^

a The result is at the limit of measurement error.

b Unreliable data due to the low emission intensity.

c COP suspensions in toluene/acetonitrile.

The solid-state emission of **im-COP-1**, **am-COP-3**, and **am-COP-4** appears as a broad signal with a maximum at approximately 500 nm, devoid of vibronic structure (Figure 8). The highly emissive COPs, **am-COP-3** and **am-COP-4**, exhibit emission lifetimes comparable to those of **am-ref-3** and **am-ref-4** measured in nonpolar solvents (Tables 1 and 2). However, no significant influence of solvent polarity on the lifetime or position of the emission maximum was observed for the polymeric emitters (Figure S30). This suggests that the excited-state relaxation pathway is determined by interactions with the polymer matrix rather than with external environment.

**Figure 8:**
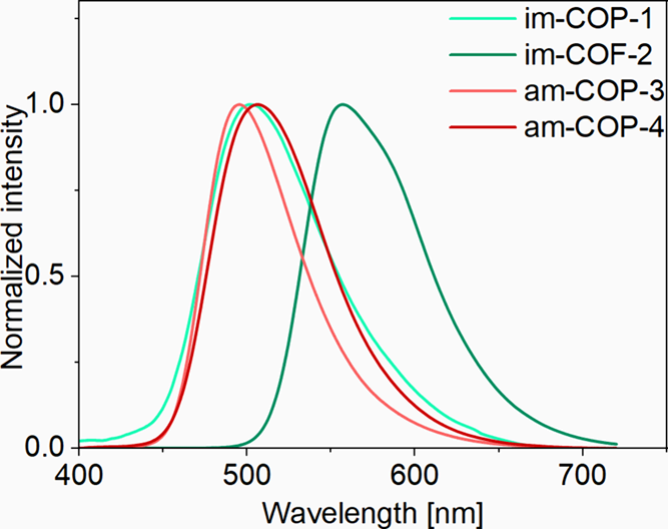
Solid-state emission spectra of imine- and amine-COF­(P)­s.

The dependence of emission on solvent polarity was also examined for both **am-COP-3** and **am-COP-4**. The obtained spectra exhibit a more subtle yet discernible response to changes in solvent polarity, indicating a form of solvatochromic behavior. As shown in Figure S27, the emission maxima for both COPs generally shift to longer wavelengths (red-shift) with an increase in solvent polarity. However, unlike the step-like and more pronounced shifts observed for the molecular reference compounds (Figure 6b,d,f,h), the emissions of COPs tend to cluster into two groups: nonpolar solvents (*n*-hexane and toluene) and more polar solvents (acetone, acetonitrile, and methanol). For **am-COP-4**, the maximum shifts from 461–469 nm in nonpolar solvents to 485–503 nm in polar solvents. Similarly, for **am-COP-3**, the shift is from 474–478 to 494–502 nm. This indicates that the polymeric matrix modulates the extent of solvatochromism compared to that of isolated molecular units.

Interestingly, the emission intensity of both **am-COP-3** and **am-COP-4** is sensitive to the water content added to the organic solvent (Figure S38). Titration of the COPs’ suspension in acetonitrile with water revealed emission quenching, reaching a plateau at 75% of the initial intensity for **am-COP-3** and 60% for **am-COP-4**.

Considering the emission features of the studied COFs, one may postulate that the emissions of **im-COP-1**, **am-COP-3**, and **am-COP-4** originate from weak charge transfer states, analogous to the reference compounds. The low quantum yield (QY) of **im-COP-1** can be explained by considering the material’s morphology and the electronic structure of its molecular reference **im-ref-1**. The LUMO* of the **im-ref-1** excited state exhibits a node at the C=N bond (Figure 7). This allows stabilization of the excited state through free rotation around the C–N bond, leading to the formation of a twisted intramolecular charge transfer (TICT) state, which subsequently decays nonradiatively.^52,53^ The C=N rotation may be restricted by the external environment; however,^54^ this is not the case for **im-COP-1**, as it is amorphous and lacks strong π–π interactions that would hinder such high-amplitude motions.

A different emission characteristic is observed for **im-COF-2**. This COF exhibits red-shifted emission, with a band maximum at 560 nm. A similar band appears for the reference compound, **im-ref-2**, in nonpolar solvents (Figure 6d). For **im-COF-2**, which is crystalline, rotation around the C=N bond is restricted by π–π interactions, similar to those in the reference compound aggregates. Moreover, the presence of intramolecular hydrogen bonds facilitates a planar conformation of the molecular skeleton. This not only explains the tendency of **im-ref-2** to form aggregated states but also promotes the formation of the crystalline phase of **im-COF-2**. Consequently, the restriction of high-amplitude motions involved in nonradiative processes, along with the formation of aggregated states, accounts for the moderate emission quantum yield of **im-COF-2**. Finally, considering the pronounced Stokes shift observed for **im-ref-2** (Figure S32) and for **im-COF-2** solid states, we propose that the low-energy emission arises via a mechanism distinct from that observed in the other imine- and amine-COF­(P)­s and their references. We speculate that in systems featuring intramolecular hydrogen bonding, the emission is induced by aggregation and originates from an excited-state intramolecular proton transfer (ESIPT) process. However, further experimental studies are required to validate this hypothesis but are outside the scope of this study.

Contrary to the scenario proposed for imine-linked COF­(P)­s, **am-COP-3** and **am-COP-4** are not prone to undergoing the TICT process. This is due to the subsequent transformation of the C=N imine into an amine group. As a result, the absence of the TICT deactivation channel leads to an increase in the emission quantum yield (QY). In detail, the high-yield emission of **am-COP-3** and **am-COP-4** originates from the weak charge transfer state. As evidenced for molecular references, relaxation of the excited state (S_1_) occurs through the rotation of phenyl substituents attached to the anthracene core, delocalizing the LUMO* orbitals over the substituents (Figure 7) and dictating the charge transfer characteristic of the HOMO*–LUMO* transition. This behavior aligns with the coupling between the locally excited state and the charge transfer state, a mechanism previously proposed for the structurally similar anthracene derivative.^55,56^ Here, the intrinsic polarity of the polymer facilitates CT emission over local excited (LE) emission.

Additional insights into the structure–property relationships of the studied COF­(P)­s were gained through cyclic voltammetry (CV) measurements performed on COF­(P) layers applied to electrode surfaces.

The CV profiles of **im-COP-1** and **im-COF-2** reveal that both materials undergo reversible redox processes. In the anodic region, the first oxidation peaks of the imine-linked COF­(P)­s appear at potentials that are 0.20–0.25 V lower than those observed for their corresponding molecular imine references (Figures S35 and S36 and Table S13). This shift suggests stabilization of the oxidized species, likely facilitated by π–π stacking interactions within the solid-state framework.

In contrast, the **amine-COPs** exhibit anodic peaks shifted to higher potentials by approximately 0.41–0.46 V, relative to those of their molecular analogues. This behavior may be attributed to limited diffusion of PF_6_^–^ counterions from the electrolyte into the more compact layer of the **amine-COPs**. Differences in film morphology are also suggested by the observation of the sharp peaks linked with charge trapping at around −0.13 to −0.14 V versus Fc/Fc^+^ for **am-COPs** in CVs recorded in a broader potential range (Figure S37). This hypothesis is corroborated by SEM analyses, which demonstrate a denser morphology for the amine COPs than for the imine-COF­(P)­s (Figure S21), as well as PXRD results, which confirm the crystalline nature of **im-COF-2** and the amorphous character of the remaining materials.

## Conclusions

In this study, imine-linked covalent organic framework **im-COF-2** and imine-linked covalent organic polymer **im-COP-1** were synthesized, exhibiting either crystalline or amorphous structure. The formation of the crystalline phase is driven by intramolecular hydrogen bonding, which promotes structural ordering. Subsequent reduction of the imine bonds afforded the corresponding amine-linked COPs, **am-COP-3** and **am-COP-4**, resulting in a significant enhancement of the photoluminescence quantum yield to 30%. To elucidate the excited-state deactivation pathways, a set of molecular references were employed.

Photophysical studies of the reference compounds demonstrated a dependence of emission spectra on solvent polarity, indicating an excited-state redistribution of electron density consistent with a weak charge transfer process. These findings are supported by DFT calculations, which revealed a common excited-state relaxation pathway for both studied imines and amines involving rotation of phenyl substituents and partial electron density transfer from the anthracene core to the adjacent phenyl groups. Additionally, **im-ref-2**, which contains an intramolecular hydrogen bond, was found to exhibit aggregate-state emission tentatively attributed to an excited-state proton transfer mechanism.

The photophysical properties of the COF­(P)­s can be rationalized through considering the properties of their molecular references. For both **im-COP-1** and **im-COF-2**, excited-state energy dissipation involves emission from the weak CT states. In amorphous **im-COP-1**, nonradiative deactivation is dominated by a twisted intramolecular charge transfer (TICT) process. In contrast, crystalline **im-COF-2**, stabilized by intramolecular hydrogen bonds, displays red-shifted emission with a quantum yield of 3%, which is attributed to increased structural rigidity suppressing nonradiative decay channels.

The amine-linked COPs, **am-COP-3** and **am-COP-4**, exhibit markedly higher emission efficiencies. Their radiative decay originates from weak CT states, whose excited-state energetics are modulated by interactions with the polymer matrix. The improved quantum yields are attributed to the elimination of nonradiative TICT pathways through the chemical reduction of the imine linkage.

These findings suggest that the absence of emission in imine-linked COFs is an inherent property of the imine group itself rather than a result of interlayer interactions in the two-dimensional COF architecture. In cases where emission is present, its intensity and nature are significantly influenced by the COF microenvironment. Further in-depth mechanistic investigations are needed to fully understand the origins and mechanisms of emission and quenching in imine-based systems.

## Supplementary Material







## Data Availability

The data sets generated and analyzed during the current study are available in the RepOD repository at 10.18150/Q9PKFX.

## Notes

General remarks, synthetic protocols, characterization of materials, and computational calculations (PDF)
DFT-optimized strutures (CIF)
DFT-optimized strutures (CIF)
