# Supplementary material for: Reductive Transformation of Imine Covalent Organic Frameworks into Emissive Polymers: Insights into Emission Quenching
Source: Chem Mater. 2025 Oct 1;37(19):8048–58. doi: 10.1021/acs.chemmater.5c01951 (PMC12529896; doi:10.1021/acs.chemmater.5c01951)
Supplement: Supplementary file 1 [file cm5c01951_si_001.pdf]

Supporting Information
for

Reductive Transformation of Imine Covalent Organic Frameworks into Emissive Polymers: Insights into Emission Quenching

Agata Tyszka-Gumkowska,[a)] Mateusz Brzeziński,[b)] Sylwester Gawinkowski, [b)] Tomasz Polczyk,[c)] Wojciech Wegner, [a)] Mateusz Wlazło,[d)] Piotr Bernatowicz, [b)] Jan Nawrocki, [b)] Przemysław Gaweł,[e)] Krzysztof Noworyta, [b)] Jakub Ostapko [a)]

a) Centre of Excellence ENSEMBLE3 Sp. z o.o., Wólczyńska 133, 01-919 Warsaw, Poland
b) Institute of Physical Chemistry, Polish Academy of Sciences, Marcina Kasprzaka 44/52, 01-224 Warsaw, Poland
c) Faculty of Chemistry, Jagiellonian University, Gołębia 24, 31-007 Kraków, Poland
d) Center of New Technologies, University of Warsaw, Stefana Banacha 2c, 02-097 Warsaw, Poland
e) Institute of Organic Chemistry, Polish Academy of Sciences, Marcina Kasprzaka 44/52, 01-224 Warsaw, Poland

Table of contents

1. **General Remarks** S1
2. **Synthesis** S10
3. **NMR spectra** S17
4. **COF synthesis optimization** S21
5. **Raman and Fourier transformed IR spectroscopy** S27
6. **PXRD** S29
7. **SEM** S31
8. **TEM** S32
9. **DSC-TGA** S33
10. **DFT** S34
11. **Spectroscopy** S39
12. **Chromaticity diagrams** S46
13. **Electrochemical measurements** S48
14. **Literature** S53

1. General Remarks

Methodology and chemical handling

All solvents and reagents were obtained from common suppliers and used as received. All non-aqueous reactions were performed in oven-dried glassware under an inert atmosphere of N_2 by means of standard Schlenk line and glovebox (GS Systemtechnik and MBraun) techniques. If needed, solvents were dried and stored with molecular sieves (4 Å). Additionally, solvent used for COFs synthesis were degassed by freeze-pump-thaw procedure repeated three times. Flash column chromatography was performed on silica gel (230–400 mesh); thin-layer chromatography (TLC) was carried out on aluminum sheets coated with SiO_2-60 F254 obtained from Merck; visualization with a UV lamp (254 or 366 nm). All products were dried under high vacuum (ca. 10^{-2} mbar) before analytical characterization.

Nuclear magnetic resonance (NMR) spectra were acquired on the Bruker AVANCE II instrument (300 MHz or 500 MHz) and Agilent DD2 400 MHz. Chemical shifts are reported in parts per million (ppm) and referenced to solvent residue peak. The splitting pattern of multiplets is described by abbreviations (s – singlet, d – doublet, t – triplet, q – quartet, dd – doublet of doublets, m –multiplet, c – covered signal, b – broad peak). Coupling constants (*J*) values are reported in Hz.

Magic angle spinning nuclear magnetic resonance (MAS NMR). The spectra were acquired at room temperature on BRUKER AVANCE II 500MHz spectrometer equipped with a 4mm DVT probehead. They were referenced to external methyl signal of glycine which was at 43.3 ppm. The sample spinning frequency was 10 kHz, acquisition time 20.5 ms, recycle delay 10 s, and cross-polarization contact time 1.5 ms.

High-resolution mass spectrometry (HRMS) measurements were performed using Synapt G2-Si mass spectrometer (Waters).

Elemental Analysis (EA) was obtained on Vario EL Cube (Elementar) apparatus.

Differential Scanning Calorimetry - Thermogravimetric Analysis (TGA) was performed on the SDT Q600 (TA Instruments) under nitrogen (50 L/ min) with a 10°C/ min temperature ramp.

Powder X-Ray Diffraction (PXRD) was performed using a Panalytical Empyrean diffractometer equipped with a Cu Kα radiation source (λ = 1.5406 Å) and operated in Bragg-Brenatano geometry. The measurement range was set to 1.8–40° 2θ, with a step size of 0.039° for synthesis optimization and a step size of 0.014° for synthesis under optimized conditions, with total acquisition times of 1 hour and 8 hours, respectively. The X-ray tube was operated at 45 kV and 40 mA. A programmable divergence slit (1/16°) and a fixed anti-scatter slit (1/8°) were employed to optimize the beam geometry and reduce background noise.

The Jana2006 program[1] was used to perform the Le Bail fit. The structure obtained from DFT calculations was used as the preliminary structural model. For the background, 30 Legendre polynomials were applied. The peak shape was modeled using a pseudo-Voigt function.

Scanning electron microscopy (SEM)

Scanning electron microscopy (SEM) was carried using FEI Nova NanoSEM 450 microscope.

Transmission electron microscopy (TEM)

TEM investigations were performed on a Talos F200X (FEI Company) microscope operated at 200 kV. The measurements were performed in TEM and scanning TEM (STEM) modes using a high-angle annular dark-field (HAADF) detector and energy-dispersive X-ray spectroscopy on a Brucker BD4 spectrometer.

Cyclic voltammetry (CV)

The voltammograms have been recorded using Reference 620 potentiostat (Gamry, Warminster, PA, USA) driven by Gamry Framework v. 7.8.2 software of the same manufacturer. All electrochemical experiments have been performed using three-electrode V-shaped mini cell designed in Institute of Physical Chemistry PAS (Warsaw, Poland). The glass carbon (GC) electrode 2 mm in diameter has been used as working electrode, while Pt wire and Ag wire has been used as counter and quasi-reference electrodes, respectively. Before measurements, GC working electrode has been cleaned by polishing with alumina of grain sizes from 3 to 0.05 µm. Then, it has been washed with deionized water, acetone and isopropanol. Ferrocene p.a. (Sigma-Aldrich) was used as internal potential reference and all potentials were recalculated versus potential of Fc/Fc^{+} redox couple. For cyclic voltammetry experiments with reference compounds **im-ref-**1 and **im-ref-2**, **am-ref-3** and **am-ref-4**, were performed in 0.1 M tetra-n-butylammonium hexafluorophosphate, $(TBA)PF_6$, solution in anhydrous dichloromethane (Sigma-Aldich) ot toluene/acetonitrile 2:1 (v : v). Both acetonitrile and toluene were anhydrous of Sigma-Aldrich. The electrochemical grade, anhydrous, $(TBA)PF_6$ of Fluke was used for all experiments. Studied amine and imine precursor concentration was at the level of 1.0 -1.7 mM. The solution was thoroughly deaerated by argon purging prior to experiments. Then the argon flow above the solution was maintained throughout the CV measurements to preserve anaerobic conditions.
In order to perform electrochemical studies, the COF material films were deposited on GC electrodes using drop-casting method. In brief, the 1 mg of the COF material was sonicated for 30 min in 1 mL of acetonitrile in order to obtain suspension. Then, 20 µL of such suspension has been drop-casted on the electrode and leaved to evaporate. This process was repeated, thus forming visible COF film on the electrode surface. The GC electrode surface was roughened by sand paper in order to increase the film adhesion. The fabricated electrodes were then immersed in 0.1 M $(TBA)PF_6$ solution in acetonitrile (anhydrous, Sigma-Aldrich). All CVs have been performed with the same cell, electrodes and equipment as used for precursors studies.

N_2 adsorption/desorption measurements were performed using a 3P Instruments Micro200 Surface Area and Porosity Analyzer at 77 K. Before analysis, samples (50mg - 100 mg) were degassed at 120 °C for 10 h under dynamic vacuum. Brunauer-Emmett-Teller (BET) method was utilized to calculate the specific surface areas. The relative pressure regimes for the BET analysis were chosen according to the criteria for evaluating BET surface areas for microporous materials.[2] The pore volumes were derived from the sorption curves using the non-local density functional theory model.

Preparation of Solutions and Suspensions

All solutions of reference compounds and suspensions of polymeric COFs were prepared in spectroscopic purity grade solvents (Sigma Aldrich, Supelco, Uvasol series). The solvents used were

freshly opened. To further purify and dry the solvents and remove dissolved oxygen, they were transferred into 20 mL vials, to which approximately 10–20 beads of 4 Å molecular sieves were added. The solvents were then transferred to an extractor, the bottom of which was also filled with identical molecular sieves (approximately 50 g). Each solvent was degassed individually by applying vacuum until bubbling ceased, followed by purging the chamber with nitrogen. This vacuum-purge cycle was repeated five times, after which the solvents were left for an additional 24 hours. The degassing process (five additional cycles) was then repeated the following day.

The solutions were prepared by dissolving a small amount of the reference compound (a few milligrams) in 20 mL of solvent, followed by vortex mixing for at least 60 s. Subsequently, a series of dilutions was performed to ensure that the absorbance in the 360–420 nm range did not exceed 0.05 (for further information please see “Absorbance Measurements of Solutions” part).

The suspensions were prepared by dispersing 5 mg of freshly synthesized and finely ground (using an agate mortar) COF powder in 20 mL of solvent. The suspensions were then mixed using a vortex mixer for at least 60 s, followed by ultrasonic treatment in a bath sonicator for 10 min without heating. Throughout all spectroscopic measurements, the suspensions were subjected to continuous gentle stirring using a micro stirring bar to prevent sedimentation of the powder at the bottom of the cuvette. Care was taken to avoid turbulent mixing or vortex formation, which could alter the effective optical path length.

Absorbance Measurements of Solutions

Absorbance measurements were performed using quartz cuvettes (Hellma, HL111-10-40) with an optical path length of 1 cm and a Shimadzu UV-Vis 2700 spectrophotometer. The baseline was established by filling both cuvettes with pure solvent and recording a spectrum in the 200–800 nm range. During the actual measurements, a cuvette filled with pure solvent was used as a reference, and spectra were recorded in the 300–700 nm range, with each measurement repeated three times. All measurements were conducted with the excitation and emission monochromator slit widths set to 5 nm, with an accumulation time of 1 nm/0.1 s and a wavelength step size of 0.5 nm. No step correction was applied during the lamp-switching which wavelengths were set at 290 nm and 1100 nm. Data acquisition was performed using the UV Probe ver. 2.43 software installed on a Windows 7 operating system.

Absorbance Data Processing for Solution

The data, recorded in ASCII format as absorbance vs. wavelength, were imported into OriginPro 2024b. For each sample, three consecutive spectra were collected. A relevant wavelength range was selected using a masking function to exclude solvent absorbance (lower wavelength range <350 nm) and part of the spectra where the solution did not exhibit any absorbance (upper wavelength range >475 nm). The spectra were then averaged to obtain a mean spectrum, which was subsequently processed using a Savitzky-Golay filter with a 5th-degree polynomial and a window size of 30 points. The processed spectrum was first globally normalized within a 0–1 scale, followed by normalization to a selected absorbance peak. The processed spectra were used to generate final plots without further modifications.

Luminescence Measurements of Solutions and Suspensions

Luminescence measurements were performed using quartz cuvettes (Hellma, HL111-10-40) with an optical path length of 1 cm and a Varian Cary Eclipse fluorescence spectrophotometer. Excitation wavelengths ranged from 360 nm to 400 nm for reference compounds and from 360 nm to 420 nm for COFs, with a step size of 10 nm. Emission data were collected in the 300–700 nm range. During each measurement, the photomultiplier voltage was adjusted to ensure that the emission peak fell within the range of 200–800 counts. If this was not achievable due to weak luminescence, the voltage was set to its maximum value. All measurements were conducted with the excitation and emission monochromator slit widths set to 5 nm, with an accumulation time of 1 nm/0.1 s or at a slower rate, with a wavelength step size of 1 nm. Data acquisition was performed using the Scan ver. 1.1(132) software installed on a Windows XP operating system.

Luminescence Data Processing for Solutions and Suspensions

The data, recorded in ASCII format as counts vs. wavelength, were imported into OriginPro 2024b for initial visualization. Spectra for each excitation wavelength were plotted, and a masking function was applied to manually remove regions corresponding to the Raman peak of the solvent (if necessary) and the excitation lamp wavelength.

The subsequent data processing varied depending on the emission properties of the compounds:

- For solution spectra of weakly emissive compounds, spectra from all excitation wavelengths were averaged to obtain a representative mean spectrum, which was then globally normalized within a 0–1 intensity scale. Due to the extensive masking of short-wavelength regions, a global multi-peak Gaussian fitting was applied using the multi-peak fit function with 4 unconstrained sub-peaks to interpolate the missing emission data. Following this correction, the fitted spectrum was globally renormalized within the 0–1 intensity scale.
- For solution spectra of highly emissive compounds, the spectrum corresponding to 380 nm excitation was selected as the representative one. This spectrum was smoothed using a Savitzky-Golay filter with a 5th-degree polynomial and a 30-point window, followed by global normalization within a 0–1 intensity scale.
- For suspension spectra, spectra from all excitation wavelengths were averaged to obtain a mean spectrum. This averaged spectrum was then smoothed using a Savitzky-Golay filter with a 5th-degree polynomial and a 50-point window, followed by global normalization within a 0–1 intensity scale.

The processed spectra were used to generate final plots without further modifications.

Reichardt's solvent polarity parameters [$E_T(30)$] were used to plot the position of the emission maxima versus solvent polarity.[3]

Quantum Yield Calculation for Solutions

The quantum yield was estimated relative to Coumarin 153, which served as the reference standard, following equation:

$$\Phi_S = \Phi_R \cdot \left(\frac{I_S}{I_R}\right) \cdot \left(\frac{A_R}{A_S}\right) \cdot \left(\frac{n^2{}_S}{n^2{}_R}\right)$$

where:

I_S,I_R – integrated florescence intensities of sample and reference

A_S,A_R – absorbances of sample and reference

n_S, n_R – refractive indices of the solvents used for the sample and reference[4,5]

Φ_R – quantum yield of reference, for Coumarine 153 in methanol $\Phi_R = 0.426$

Coumarin 153 solution was prepared in methanol using the same procedure as for other samples, and its absorbance and emission spectra were measured as described in the Preparation of Solutions and Suspensions, Absorbance Measurements of Solutions, and Luminescence Measurements of Solutions and Suspensions sections respectively. However, for emission measurements, care was taken to ensure that the instrument settings matched those used for the respective sample.

All data were processed using OriginPro 2024b. For each excitation wavelength, the integrated fluorescence intensities were calculated. If necessary, a masking function was applied to manually remove regions corresponding to solvent Raman peaks and the excitation lamp wavelength. The spectra were then smoothed using the Loess smoothing method with a span proportion of 0.1–0.3, and the area under the processed spectra was integrated using the peak analyzer function. Absorbance values were directly read at each excitation wavelength from three consecutive absorbance spectra. The quantum yield was then calculated for each excitation wavelength, and the final value was reported as the mean quantum yield with its standard deviation.

Scattering Measurements of Solid States

Measurements were performed using a Horiba Fluorolog modular spectrofluorometer (1997) equipped with an integrating sphere QuantaPhi-2. The experiments were conducted in Integrating Sphere (IS) mode. The powdered sample was speeded evenly across the full surface of a PTFE disk (holder) and was then covered with a quartz glass plate. The prepared sample was positioned at the bottom of the integrating sphere. The incident light port was directed from above, directly onto the sample, while the exit port was connected to the rear part of the sphere, forming a 90-degree angle between the two ports. All measurements were performed with an accumulation time of 1 nm/1 s and a wavelength step size of 0.5 nm. Data were collected from the main detector (within its linear range, up to 1 million counts) and the reference detector. The excitation and emission monochromator slit widths were set to 3 nm. The im-COF1 sample was excited with a 360 nm line and its scattering was recorded in a range of 340-380 nm. The rest of the samples were excited with a 380 nm line and their scatterings were collected in the range of 360-400 nm. Neutral density filters (Edmund Industrial Optics) OD3.0 for **im-COP-1** and **im-COF-2** and OD2.5 for **am-COP-1** and **am-COP-2** were used. The reference measurements were proceeded identically by replacing the samples with fine-grounded $BaSO_4$ powder. Data acquisition was carried out using the FluorEssence ver. 3.51.20 with Origin ver. 8.1090 software, installed on a Windows XP operating system.

Luminescence Measurements of Solid States

Measurements were performed using a Horiba Fluorolog modular spectrofluorometer (1997) equipped with an integrating sphere QuantaPhi-2. The experiments were conducted in two modes:

- Integrating Sphere (IS) mode – The powdered sample was spread evenly across the full surface of a PTFE disk (holder) and was then covered with a quartz glass plate. The prepared sample was positioned at the bottom of the integrating sphere. The incident light port was directed from above, directly onto the sample, while the exit port was connected to the rear part of the sphere, forming a 90-degree angle between the two ports.
- ii) Front-Face (FF) mode – The powdered sample was placed between two quartz plates (cuvettes QM2704) to achieve a uniform thickness of approximately 1 mm. The sample was then positioned in a rotating holder. It was aligned so that the incident beam formed an angle of approximately 5 degrees with respect to the normal of the quartz plate.

All measurements were performed with an accumulation time of 1 nm/1 s and a wavelength step size of 1 nm. Data were collected from the main detector (within its linear range, up to 1 million counts) and the reference detector. The excitation and emission monochromator slit widths were set to 3 nm (IS) and 1.8 nm (FF). The im-COF1 sample was excited with a 360 nm line and its luminescence was recorded in a range of 375-640 nm. The rest of the samples were excited with a 380 nm line and their luminescences were collected in the range of 400-720 nm. No filter was used for this type of measurement. For both configurations reference measurements were proceeded identically by replacing the samples with fine-grounded $BaSO_4$ powder. Data acquisition was carried out using the FluorEssence ver. 3.51.20 with Origin ver. 8.1090 software, installed on a Windows XP operating system.

Luminescence Data Processing of Solid States

The data, recorded in .opj format, were processed using OriginPro 2024b. For the emission spectra presented in the main text and SI, only data recorded in the FF mode were used, as this configuration minimizes the likelihood of reemission and reabsorption artifacts. The spectrum for im-COF1 was obtained by subtracting the reference sample spectrum, measured in the same configuration, from the raw emission spectrum. Before subtraction, both spectra were normalized to the maximum intensity of the reference spectrum to recalibrate the counts. The resulting corrected spectrum, free from residual emission contributions, was then smoothed using a Savitzky-Golay filter with a 5th-degree polynomial and a window size of 50 points. Finally, the processed spectrum was globally normalized within a 0–1 intensity scale. For the remaining samples, no such correction was necessary, as the contribution of residual emission was negligible in comparison to their intrinsic emission. These spectra were only smoothed using and globally normalized as for **im-COP-1**. The processed spectra were used to generate final plots without further modifications.

Quantum Yield Calculation for Solid State

The quantum yield was estimated using $BaSO_4$ as a scattering reference following the equation:

$$\Phi_S = \frac{L_{S\,em}}{L_{S\,abs}} = \frac{L_{S\,em}}{L_{R\,scat} - L_{S\,scat}}$$

where:

$L_{S\,em}$ – integrated total florescence intensities of sample

$L_{S\,abs}$ – total absorbance of sample

$L_{R\,scat}$, $L_{S\,scat}$ – integrated total scattering intensities of reference and sample

All data were processed using OriginPro 2024b. For each sample, the raw emission intensity was corrected using a spectral sensitivity correction curve, following Maroncelli's method.[6,7] The corrected spectra were then integrated using the peak analyzer function. Scattering intensities for the samples were adjusted by applying the Rayleigh correction factor for UV excitation (0.33) and were further weighted by the transmission curves of neutral density (grey) filters. The resulting corrected scattering spectra were integrated using the peak analyzer function, and the obtained values were used to compute the final quantum yield.

Lifetime Measurements for Solutions and Suspensions

Lifetime measurements were conducted using the Time-Correlated Single Photon Counting (TCSPC) method with quartz cuvettes (Hellma, HL111-10-40) of 1 cm optical path length. The measurements were performed using an Edinburgh nF900 spectrometer, equipped with a CD900 router and a 370 nm diode controlled by IBH NanoLED a driver operating at a 300 kHz repetition rate. The diode was positioned in a standard 90-degree configuration relative to the monochromator entrance and was focused on the cuvette surface. The measurements were performed in the presence of a 420 nm cutoff filter (Thorlabs, FGL420S – 2) placed on the emission side. The monochromator was set to collect emission at 520 nm, with slit widths adjusted to 3 nm. Data acquisition was conducted using 1024 channels of the counting card, corresponding to a time window of 0–62.5 ns. The count rate was carefully maintained below 3000 counts per second, and data collection continued until 2000 counts were recorded at the peak. A reference instrument response function (IRF) measurement was performed at the end of each measurement series. This was carried out using an aqueous micelle solution, with the cutoff filter removed, the detection wavelength set to 370 nm, and the monochromator slit widths unchanged. Data acquisition was performed using F900 ver. 5.13 software, installed on a Windows 98 operating system.

Lifetime Calculation for Solutions and Suspensions

Lifetime decay and IRF curves were globally normalized within a 0–1 scale and deconvoluted by subtracting the IRF signal using a custom Python script based on the Richardson-Lucy deconvolution method. The deconvoluted decay data were then transferred to OriginPro 2024b, where they were fitted using an exponential decay function. The fitting process was constrained to the lowest number of decay components necessary for an adequate fit. In this case, a two-component exponential model provided a satisfactory fit, yielding an $R^2 > 0.9$. Only the second-lifetime component was considered in the final analysis, as the first decay component was below 1 ns, representing a residual signal related to

the IRF rather than the intrinsic lifetime of the sample. The deconvoluted decay data and their respective fits were used to generate the final plots without further modifications.

Luminescence Chromaticity Graphs

Chromaticity graphs in the CIE 1931 color space were generated using a custom Python script based on the Colour Science, Color library.[8] The script was designed to accurately map emission spectra onto the CIE chromaticity diagram, ensuring precise color representation.

Raman Spectroscopy Measurements

Raman spectroscopy measurements were performed *in-situ* using Raman microscopy realized by the Renishaw system inVia, a Leica microscope DM2500M, and a Leica objective 50×/0.75 N PLANE EPI. A He-Ne 633 nm laser (Renishaw) was used as the excitation source, with laser power set in the range of 3–5 mW. Measurements were conducted on solid powdered compounds dispersed on a coverslip, recording a Raman map with a minimum area of 20 µm × 20 µm, a 1 µm step size, and an integration time of 1 spectrum/s within the spectral range of 0–1800 cm^{-1}. The measurement area was selected to be representative of the entire sample, ensuring that the mapping was performed within a single granule of the powder. Data acquisition was carried out using the WiRe ver. 5.6 software, installed on a Windows 11 operating system.

Raman Spectroscopy Data Processing

The data, recorded in .wdf format, were processed using WiRe version 5.6. The processing began with loading all spectra corresponding to individual measurement points within the mapped area. Using the trimming function, the peak originating from the laser line was removed. Next, a component analysis was performed, selecting only the components that contributed significantly across the entire mapped surface. Baseline correction was applied using a 12th-degree polynomial fitting, with the noise level set to 2. The processed data were then smoothed using the Savitzky-Golay method with a 5th-degree polynomial and a window size of 15. Afterward, the spectra from all measurement points were averaged and globally normalized within a 0–1 intensity scale. The final normalized intensity vs. Raman shift data were exported as an ASCII file and imported into OriginPro 2024b. No further processing was applied, and the data were directly used for plotting the final graphs.

FT-IR Spectroscopy Measurements

FT-IR spectroscopy measurements were performed using a Nicolet Magna-IR 560 spectrometer. The analyzed compound was prepared in the form of a compressed pellet with a diameter of ½ inch and a thickness of 1 mm. The pellet was prepared by grinding approximately 2 µg of the sample with 200 mg of KBr in an agate mortar, followed by compression of the powder mixture in a pellet die using a hydraulic press at 100 atm. The KBr used for pellet preparation was preheated for at least 24 h at 200 °C to remove adsorbed water and then cooled to room temperature before mixing and grinding. A background measurement (reference scan) was conducted using an identically prepared pure KBr pellet, without the analyzed compound. During measurements, the spectrometer chamber was continuously purged with nitrogen. Spectra were recorded in the range of 500–4000 cm^{-1} with a wavelength step size

of 0.5 cm^{-1}, using an aperture setting between 8–10 and a signal gain of 1×. In total 256 single spectra were automatically averaged to produce final data. Data acquisition was carried out using the Omnic E.S.P ver. 5.0 software, installed on a Windows 11 operating system.

FT-IR Spectroscopy Data Processing

The data, recorded in ASCII format as absorbance vs. wavenumber, were imported into OriginPro 2024b for initial visualization. Baseline correction was performed using the Peak Analyzer function to account for the reference KBr pellet residual contribution. The user-defined BSpline mode was applied, with the number and positions of anchor points manually selected based on the curvature of the raw spectrum. Following baseline subtraction, the data were globally normalized within a 0–1 scale, and the processed spectra were used to generate the final plots without further modifications.

Computational Modeling

Quantum-chemical simulations were performed using the Gaussian16 software package. The CAM-B3LYP/6-31++G(d,p) combination of functional and basis set was used for the optimization of ground-state geometries and the simulation of Raman and IR spectra. For the optimization of electronically excited-state geometries and the calculation of electronic transition energies, time-dependent density functional theory (TD-DFT) with the same CAM-B3LYP/6-31++G(d,p) level of theory was employed. All geometry optimizations were followed by normal mode analysis, and the identification of stationary points was confirmed by the absence of imaginary frequencies. Simulated Raman and IR spectra were generated assuming a Lorentzian band shape with a full width at half maximum (FWHM) of 5 cm^{-1}. The resulting spectra were scaled by a factor of 0.97 for comparison with experimental data.

A planewave DFT calculation was performed as implemented in the package VASP.[9–12] The electronic exchange and correlation effects were treated by the Perdew−Burke−Ernzerhof (PBE) functional[13] with PAW-type pseudopotentials.[14] PBE-D3(BJ) theory level was applied to take into account the vdW interactions.[15,16] A planewave basis set cutoff of 500 eV was used, and Brillouin zone integration considered only the Γ point. Due to periodic boundary conditions, a 20 Å-thick layer of vacuum was introduced above the monolayer to eliminate interactions between neighboring unit cells. The system was relaxed until forces acting on each atom vanished below 0.05 eV/Å.

The eclipsed AA structure was obtained by doubling the monolayer unit cell in the c direction with an initial interlayer spacing of 3.5 Å. To create the staggered AB structure, the atoms from the upper layer were shifted by [⅓a, -⅔b]. Both structures were then relaxed to the same accuracy as the initial monolayer unit cell.

2. Synthesis

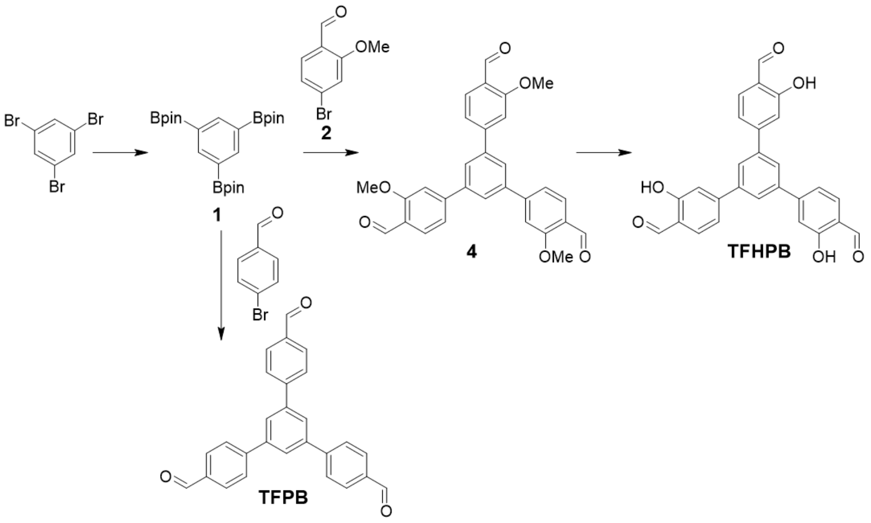

Scheme 1. Synthesis of aldehydes used for COFs synthesis.

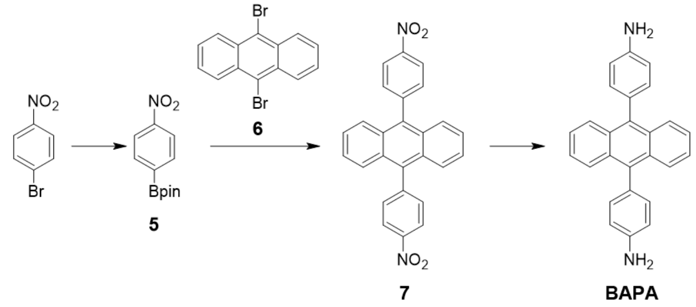

Scheme 2. Synthesis of **BAPA**.

1,3,5-Tris(4,4,5,5-tetramethyl-1,3,2-dioxaborolan-2-yl)benzene (1)

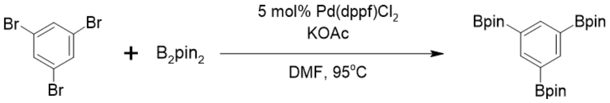

Using the Schlenk technique, 1,3,5-tribromobenzene (2.0 g, 6.4 mmol, 1.0 equiv.), bis(pinacolato)diboron (B_2pin_2, 5.2 g, 20.5 mmol, 3.2 equiv.), potassium acetate (KOAc, 3.77 g, 38.4 mmol, 6.0 equiv.), and $Pd(dppf)Cl_2$ (0.234 g, 0.32 mmol, 0.05 equiv.) were placed in a flask, and anhydrous DMF (20 mL) was added. The reaction mixture was degassed and stirred at 95 °C overnight. After that time, water was added and reaction mixture was extracted with ethyl acetate (3x20 mL). Collected organic fractions were washed with water (3x10 mL), brine and dried over Na_2SO_4. The organic fraction was then passed through a silica gel pad (15 cm height, 3 cm diameter), eluting with an additional portion of ethyl acetate. After evaporating the solvent, the residue was dissolved in 50 mL of hot hexane, and the residual slurry was filtered to remove any insoluble material. The filtrate was then left in the freezer for 2 hours, resulting in the formation of a precipitate (2.0 g), which was filtered and collected. The filtrate was partially evaporated and returned to the freezer, yielding an additional portion of the desired product (0.43 g) as colorless crystals. The total yield was 2.43 g (5.33 mmol, 83%).

^1H NMR (300 MHz, $CDCl_3$) δ 8.40 (s, 1H), 1.37 (s, 12H). The characterization data agrees with the literature.[15]

4-Nitrophenylboronic acid pinacol ester (5)

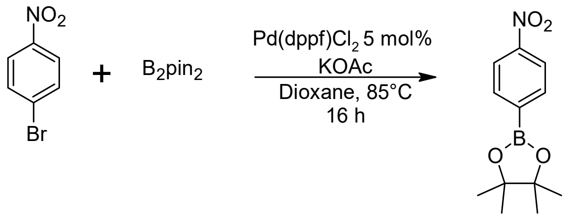

Using the Schlenk technique, 1-Bromo-4-nitrobenzene (3.74 g, 18.5 mmol, 1.0 equiv.), bis(pinacolato)diboron (B_2pin_2, 5.64 g, 22.2 mmol, 1.2 equiv.), potassium acetate (KOAc, 3.63 g, 37.0 mmol, 2.0 equiv.), and Pd(dppf)Cl_2 (0.677 g, 0.925 mmol, 0.05 equiv.) were placed in a flask, and anhydrous dioxane (50 mL) was added. The reaction mixture was degassed and stirred at 85 °C overnight. After that time, the dioxane was evaporated, and the residue was re-dissolved in CH_2Cl_2, then washed with water and brine. The organic phase was dried over Na_2SO_4, filtered, and passed through a silica gel pad (15 cm height, 3 cm diameter), eluted with an additional portion of the CH_2Cl_2. After concentrating the solvent, the residue was dissolved in a minimal amount of hexane, and the residual slurry was filtered to remove any insoluble material. The filtrate was then left in the freezer for 2 hours. The formed precipitate was filtered to yield the desired product as colorless crystals (4.275 g, 17.2 mmol, 93%). ^1H NMR (500 MHz, $CDCl_3$) δ 8.19 (d, J = 10 Hz, 2H), 7.96 (d, J = 10 Hz, 2H), 1.37 (s, 12H). The characterization data agrees with the literature.[16]

4-Bromo-2-methoxybenzaldehyde (2)

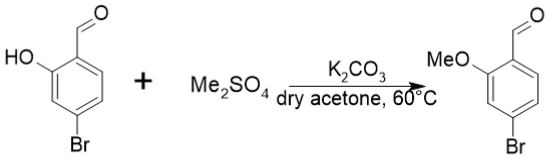

4-Bromo-2-methoxybenzaldehyde (5.0 g, 24.85 mmol, 1 equiv.) was dissolved in dry acetone (125 mL) containing molecular sieves (4 Å) in a pressure ampoule. K_2CO_3 (10.3 g, 74.55 mmol, 3 equiv.) was added, followed by dimethyl sulfate (2.8 mL, 29.82 mmol, 1.2 equiv.). The ampoule was then sealed, and the mixture was stirred at 60 °C for 1 hour. The reaction progress was monitored by TLC (5% ethyl acetate in hexane), indicating full conversion of the starting material. The mixture was then filtered, and the collected filtrate was concentrated. The solid residue was dissolved in ethyl acetate (200 mL), and the organic phase was washed with saturated $NaHCO_3$, filtered, and the solvent was removed. The resulting solid was dried under reduced pressure (3×10^{-3} mbar) for 2 hours, yielding the product as a light brownish solid (4.7 g, 21.86 mmol) with an 88% yield. ^1H NMR (300 MHz, $CDCl_3$) δ 10.43 (s, 1H), 7.72 (d, J = 8.1 Hz, 1H), 7.33 – 7.20 (m, 2H), 3.97 (s, 3H). The characterization data agrees with the literature.[17]

9,10-Dibromoantharecene (6)

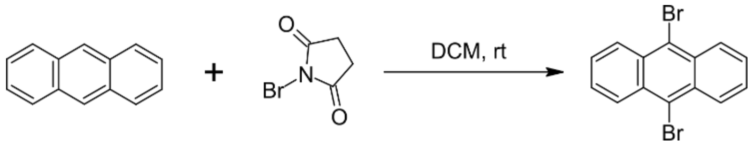

Anthracene (5.0 g, 0.028 mol, 1.0 equiv.) was dissolved in 250 mL of dichloromethane at room temperature, and N-bromosuccinimide (NBS, 10.24 g, 0.058 mol, 2.05 equiv.) was added in one portion. The reaction mixture was stirred at room temperature for 3 hours, after which the CH_2Cl_2 was evaporated. The resulting solid was suspended in methanol (MeOH, 180 mL), and the precipitate was filtered, washed with an additional portion of MeOH (20 mL), and dried to afford a light green solid of 9,10-dibromoanthracene (8.5 g, 0.0253 mol, 90%). ^{1}H NMR (500 MHz, $CDCl_3$) δ 8.60 (dd, J = 6.8, 3.2 Hz, 1H), 7.65 (dd, J = 6.8, 3.1 Hz, 1H).The characterization data agree with the literature.[18]

9,10-bis(4-nitrophenyl)anthracene (7).

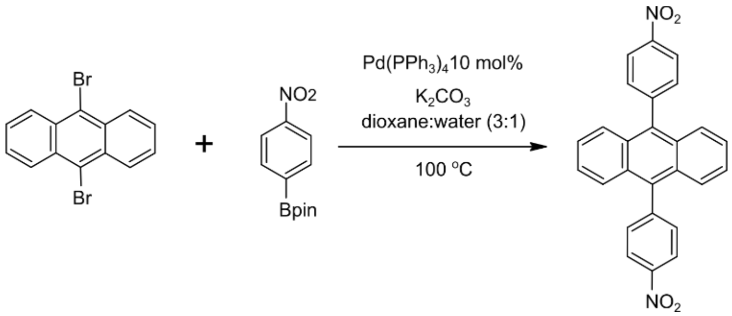

9,10-Dibromoantharecene (2.66 g, 7.92 mmol, 1 equiv.), boronic ester **5** (4.34 g, 17.43 mmol, 2.2 equiv.), K_2CO_3 (10.95 g, 79.2 mmol, 10 equiv.) and $Pd(PPh_3)_4$ (92 mg, 0.792 mmol, 0.1 equiv.) were placed into a Schlenk flask containing a mixture of solvents (160 mL, dioxane:water, 3:1 v/v), degassed, and stirred at 100 °C for 3 hours. After cooling to room temperature, the yellow solid was filtered, washed with MeOH (4x20 mL) and dried to give 9,10-bis(4-nitrophenyl)anthracene as yellow solid (2.24 g, 5.33 mmol, 67%). The NMR spectra could not be obtained due to the low solubility of the material. HRMS (APCI) calcd for $C_{26}H_{16}N_2O_4$ $(M)^-$: 420.1110, found 420.1106.

9,10-bis(4-aminophenyl)anthracene (BAPA).

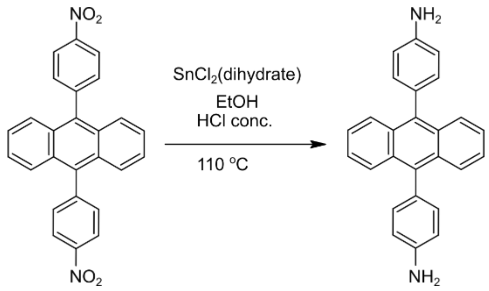

9,10-bis(4-nitrophenyl)anthracene (1.8 g, 4.28 mmol, 1 equiv.) and $SnCl_2{\cdot}2H_2O$ (23.2 g, 102.8 mmol, 24 equiv.) were suspended in EtOH (96 mL) and concentrated HCl_{aq} (35 mL) in an ampoule. The reaction was stirred at 110 °C for 15 h. After this time, the white precipitate of the product was filtered and washed with a small amount of EtOH. The solid was then suspended in ethyl acetate (50 mL), and 10%

$NaHCO_3$ was added until the pH was basic. Phase were separated, and the aqueous phase was extracted with ethyl acetate (5x50 mL). The organic phase was washed with water and brine, and dried over Na_2SO_4. The solvent was evaporated, and the residue was suspended in hot MeOH and filtered, washed with MeOH, and dried to give a yellow solid. The solid was dissolved in a small amount of CH_2Cl_2 (ca. 40 mL), and hexane (10 mL) was slowly added until turbidity appeared. The mixture was left in the freezer. The yellow crystals were filtered off and additionally purified by dry-column vacuum chromatography (silicagel pad 15 cm height, 3 cm diameter), eluting with CH_2Cl_2 to give the 9,10-bis(4-aminophenyl)anthracene (1.19 g, 3.30 mmol, 77%). ^{1}H NMR (400 MHz, DMSO-d_6) δ 7.70 (dd, J = 6.8, 3.3 Hz, 4H), 7.35 (dd, J = 6.9, 3.3 Hz, 4H), 7.05 (d, J = 8.3 Hz, 4H), 6.80 (d, J = 8.3 Hz, 4H), 5.29 (s, 4H).The characterization data agree with the literature.[19]

1,3,5-Tris(4-formylphenyl)benzene (TFPB)

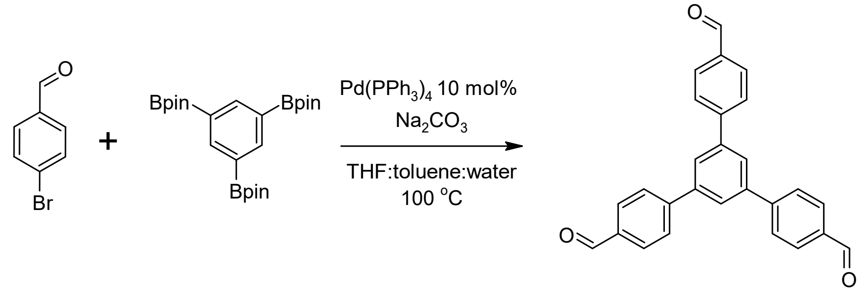

The boronic ester **1** (500 mg, 1.1 mmol, 1 equiv.), bromobenzaldehyde (812 mg, 4.39 mmol, 4 equiv.), Na_2CO_3 (1.16 g, 10.97 mmol, 10 equiv.), and the palladium catalyst (127 mg, 0.110 mmol, 0.1 equiv.) were placed in a Schlenk flask containing 15 mL of a degassed solvent mixture (THF:toluene:water, 6:6:3, v/v/v). The reaction mixture was further degassed and stirred at 100 °C for 15 h. After cooling to room temperature, the reaction mixture solidified. Methanol (8 mL) was then added, and the solid was filtered and washed with water and methanol. The solid was recrystallized from 25 mL of boiling acetone and left in the freezer for 3 hours. The resulting solid was filtered, washed with a small amount of methanol, and dried. The desired compound was obtained as a colorless solid (380 mg, 0.973 mmol, 89% yield).^{1}H NMR (400 MHz, $CDCl_3$) δ 10.11 (s, 3H), 8.06 – 8.00 (m, 6H), 7.91 (s, 3H), 7.90 – 7.84 (m, 6H).The characterization data agree with the literature.[20]

1,3,5-Tris(4-formyl-3-methoxyphenyl)benzene (4)

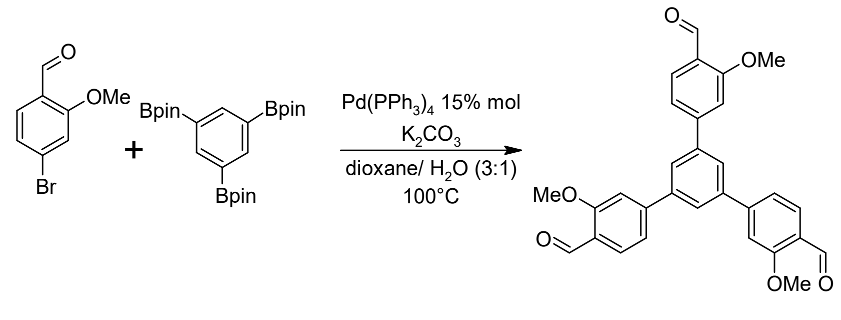

Compound **2** (1.25 g, 5.84 mmol, 3.6 equiv.), boronic ester **1** (0.74 g, 1.623 mmol, 1 equiv.), potassium carbonate (2.24 g, 16.23 mmol, 10 equiv.), and tetrakis(triphenylphosphine)palladium(0) (0.28 g, 0.24

mmol, 0.15 equiv.) were placed into a Schlenk tube. Dioxane (45 mL) and water (15 mL) were then added. The mixture was degassed by applying vacuum and introducing nitrogen into the Schlenk tube five times. The reaction mixture was stirred at 100°C for 2 hours, during which a white precipitate formed. The precipitate was filtered off and washed with acetone, water, and methanol. The crude product (735 mg) was then crystallized from hot N,N-dimethylacetamide (10 mL, 160°C). After cooling, the precipitated material was collected by filtration and washed with acetone. After air drying, the product was obtained as a light grey solid (640 mg, 1.33 mmol) with an 82% yield. ^{1}H NMR (400 MHz, cdcl3) δ 10.53 (s, 1H), 7.97 (d, J = 8.0 Hz, 1H), 7.82 (s, 1H), 7.34 (d, J = 8.0 Hz, 1H), 7.23 (s, 2H), 4.03 (s, 3H).The characterization data agree with the literature.[21]

1,3,5-Tris(4-formyl-3-hydroxyphenyl)benzene (TFHPB)

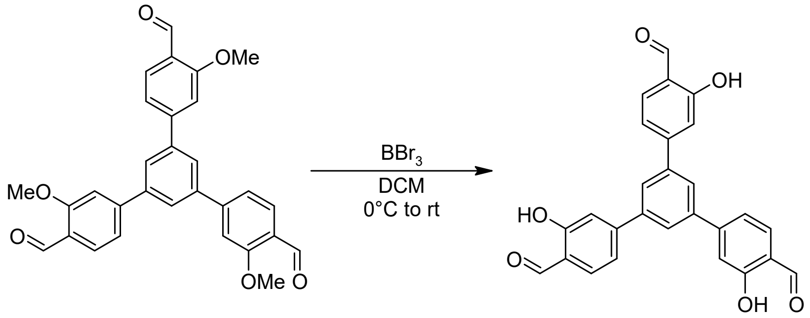

To a suspension of compound 4 (1.0 g, 2.08 mmol, 1 equiv.) in dry dichloromethane (300 mL), under a nitrogen atmosphere and cooled to 0 °C in an ice bath, a solution of BBr_3 (10.5 mL, 10.4 mmol, 1 M in CH_2Cl_2, 5 equiv.) was added dropwise. The mixture was then stirred overnight, allowing it to reach room temperature. Next, an aqueous $NaHCO_3$ solution (5%, 30 mL) was added, and the organic solvent was evaporated. The residue was filtered, washed with water and methanol, and then dried. The crude product (810 mg) was crystallized from boiling N,N-dimethylacetamide (20 mL, 165 °C). After cooling, the precipitated material was collected by filtration and washed with N,N-dimethylacetamide (5 mL) and methanol (20 mL). The final product was dried in air, yielding a light grey solid (766 mg, 1.75 mmol) with an 84% yield. ^{1}H NMR (400 MHz, DMSO-d_6) δ 10.90 (s, 1H), 10.28 (s, 1H), 7.98 (s, 1H), 7.80 (d, J = 8.1 Hz, 1H), 7.49 (dd, J = 8.0, 1.5 Hz, 1H), 7.43 (d, J = 1.6 Hz, 1H).The characterization data agree with the literature.[21]

p-[(E)-(3,5-Di-tert-butylphenyl)methyleneamino](10-{p-[(E)-(3,5-di-tert-butylphenyl)methyleneamino]phenyl}-9-anthryl)benzene (**im-ref-1)**

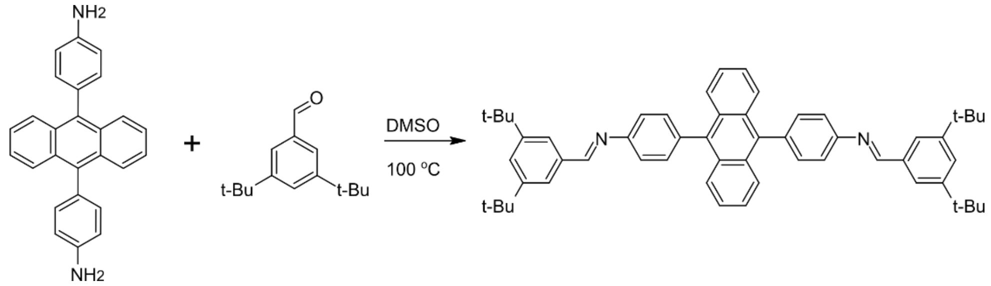

9,10-bis(4-aminophenyl)anthracene (**BAPA**) (0.6 g, 1.66 mmol, 1 equiv.) and 3,5-di-*tert*-butylbenzaldehyde (0.91 g, 4.16 mmol, 2.5 equiv.) were placed into a round bottom flask and DMSO (7.5 mL) was added. The reaction mixture was stirred at 100 °C for 1 h. The formed precipitate was filtered

and washed with EtOH (20 mL) to give desired compound as light yellowish powder (1.216 g, 1.6 mmol, 95%). Sample for the NMR characterization was prepared by an additional crystallization from dioxane. ^{1}H NMR (400 MHz, *o*-DCB-d$_4$) δ 8.79 (s, 1H), 8.11 (d, J = 1.8 Hz, 2H), 8.08 (dd, J = 6.8, 3.3 Hz, 2H), 7.80 (t, J = 1.8 Hz, 1H), 7.65 (s, 4H), 7.48 (dd, J = 6.9, 3.3 Hz, 2H), 1.51 (s, 18H). ^{13}C NMR (126 MHz, *o*-DCB-d$_4$) δ 161.23, 151.90, 151.38, 136.96, 136.50, 136.22, 125.88, 125.32, 123.59, 121.28, 34.84, 31.38. HRMS (ESI, m/z) calcd for $C_{56}H_{61}N_2$ $(M+H)^+$: 761.4835, found 761.4829.

4,6-Di-tert-butyl-2-[(E)-[p-(10-{p-[(E)-(3,5-di-tert-butyl-2-hydroxyphenyl)methyleneamino]phenyl}-9-anthryl)phenylimino]methyl]phenol **(im-rerf-2)**

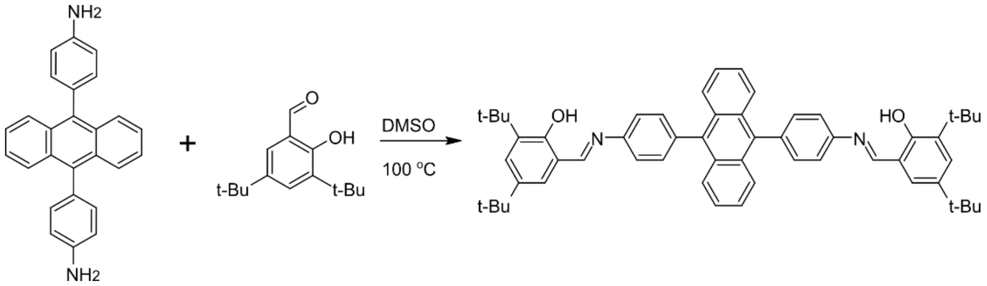

9,10-bis(4-aminophenyl)anthracene (**BAPA**) (0.2 g, 0.55 mmol, 1 equiv.) and 3,5-di-*tert*-butyl-hydroxy-benzaldehyde (0.325 g, 1.38 mmol, 2.5 equiv.) were placed into a round bottom flask and DMSO (2.5 mL) was added. The reaction mixture was stirred at 100 °C for 1 h. After 45 min the formed precipitate was filtered off and washed with DMSO (5 mL), and ethanol (5 mL) to give desired compound as light yellowish powder (405 mg, 0.51 mmol, 93%). ^{1}H NMR (500 MHz, *o*-DCB-d$_4$) δ 13.99 (s, 1H), 8.74 (s, 1H), 7.95 (dd, J = 6.8, 3.3 Hz, 2H), 7.66 (d, J = 2.2 Hz, 1H), 7.54 (d, J = 8.2 Hz, 2H), 7.44 (d, J = 8.2 Hz, 2H), 7.41 (dd, J = 6.9, 3.1 Hz, 2H), 7.38 (d, J = 2.2 Hz, 1H), 1.67 (s, 9H), 1.43 (s, 9H). 13C NMR (126 MHz, *o*-DCB-d$_4$) δ 163.87, 158.64, 147.81, 140.73, 137.51, 136.97, 136.68, 128.26, 125.43, 121.35, 118.67, 35.17, 34.14, 31.47, 29.55. HRMS (ESI, m/z) calcd for $C_{56}H_{61}N_2O_2$ $(M+H)^+$: 793.4733, found 793.4726.

p-{[(3,5-Di-tert-butylphenyl)methyl]amino}[10-(p-{[(3,5-di-tert-butylphenyl)methyl]amino}phenyl)-9-anthryl]benzene (**am-ref-**

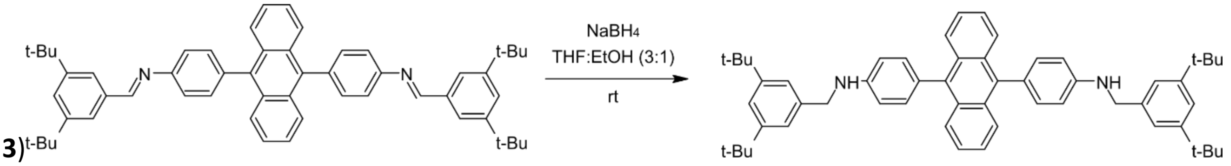

3)

Im-ref-1 (100 mg, 0.131 mmol, 1 equiv.) and NaBH$_4$ (99.4 mg, 2.63 mmol, 20 equiv.) were placed into a round bottom flask, and solvents were added (THF, 3 mL and EtOH, 1 mL). After 2 hours, 15 mL of MeOH was added and the reaction mixture stirred for an additional 2 hours. The formed precipitate was filtered and washed with an additional portion of MeOH. The solid was dried to give desired compound as light yellowish powder (95 mg, 0.124 mmol, 95%). ^{1}H NMR (400 MHz, *o*-DCB-d$_4$) δ 7.84 (dd, J = 6.8, 3.3 Hz, 4H), 7.35 (t, J = 1.8 Hz, 2H), 7.25 (d, J = 1.3 Hz, 4H), 7.17 (dd, J = 6.8, 3.2 Hz, 4H), 7.15 – 7.10 (m, 4H), 6.75 – 6.67 (m, 4H), 4.26 (s, 4H), 3.98 (s, 2H), 1.23 (s, 36H). ^{13}C NMR (126 MHz, *o*-DCB-d$_4$) δ 151.09, 147.88, 138.46, 137.33, 130.77, 124.78, 122.32, 121.39, 112.92, 49.26, 34.73, 31.45. HRMS (ESI, m/z) calcd for $C_{56}H_{65}N_2$ $(M+H)^+$: 765.5148, found 765.5146.

2,4-Di-tert-butyl-6-({p-[10-(p-{[(3,5-di-tert-butyl-2-hydroxyphenyl)methyl]amino}phenyl)-9-anthryl]phenylamino}methyl)phenol (**am-ref-4**)

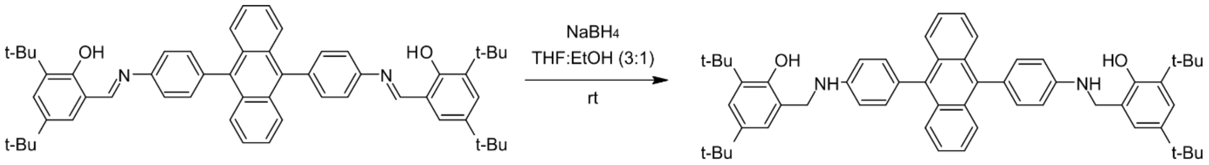

Im-ref-2 (180 mg, 0.227 mmol, 1 equiv.) and $NaBH_4$ (171.7 mg, 4.54 mmol, 20 equiv.) were placed into a round bottom flask, and solvents were added (THF, 5.2 mL and EtOH, 1.7 mL). After 2 hours, 15 mL of MeOH was added and reaction mixture was stirred for an additional 2 hours. The formed precipitate was filtered and washed with additional portion of MeOH. The solid was dried to give desired compound as light yellowish powder (172 mg, 0.216 mmol, 95%). ^1H NMR (500 MHz, *o*-DCB-d_4) δ 8.52 (s, 1H), 7.94 (dd, J = 6.6, 3.0 Hz, 2H), 7.50 (s, 1H), 7.37 (dd, J = 6.8, 2.8 Hz, 2H), 7.34 (d, J = 8.0 Hz, 2H), 7.19 (d, 2H, overlaps with solvent signal), 6.99 (s, 1H, overlaps with solvent signal), 4.37 (s, 2H), 3.83 (s, 1H), 1.61 (s, 9H), 1.42 (s, 9H). ^{13}C NMR (126 MHz, *o*-DCB-d_4) δ 153.52, 146.68, 141.71, 136.88, 136.30, 131.19, 125.09, 124.05, 123.71, 122.51, 115.98, 49.64, 35.03, 34.20, 31.71, 29.83. HRMS (ESI, m/z) calcd for $C_{56}H_{65}N_2O_2$ $(M+H)^+$: 797.5046, found 797.5048.

3. NMR spectra

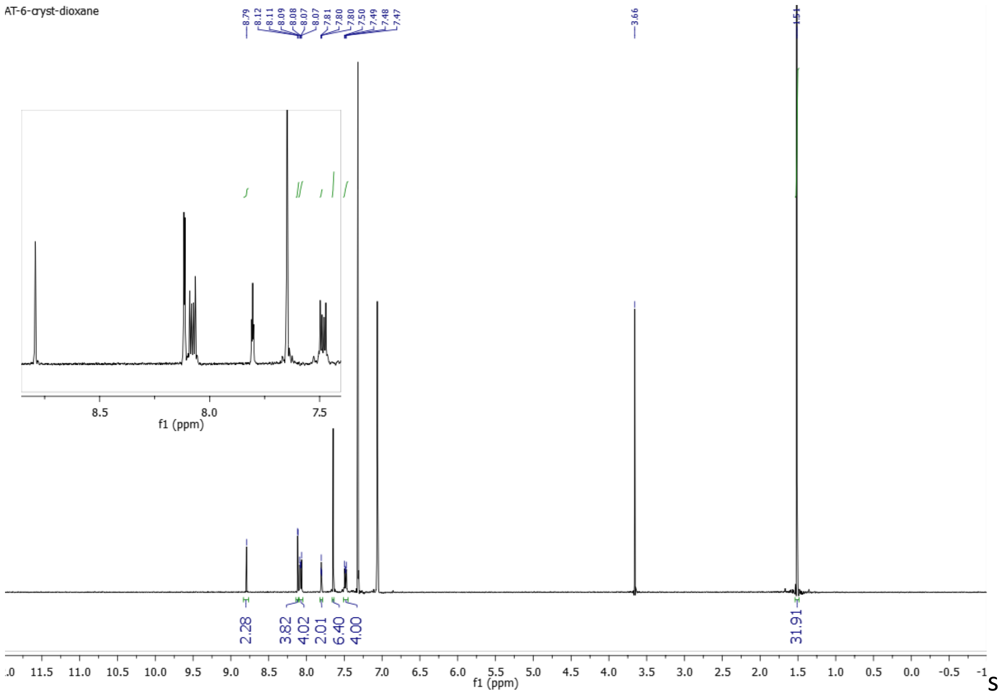

Figure S1. ^{1}H NMR spectrum of **im-ref-1** in *o*-DCB-d_4 (400 MHz, 298 K).

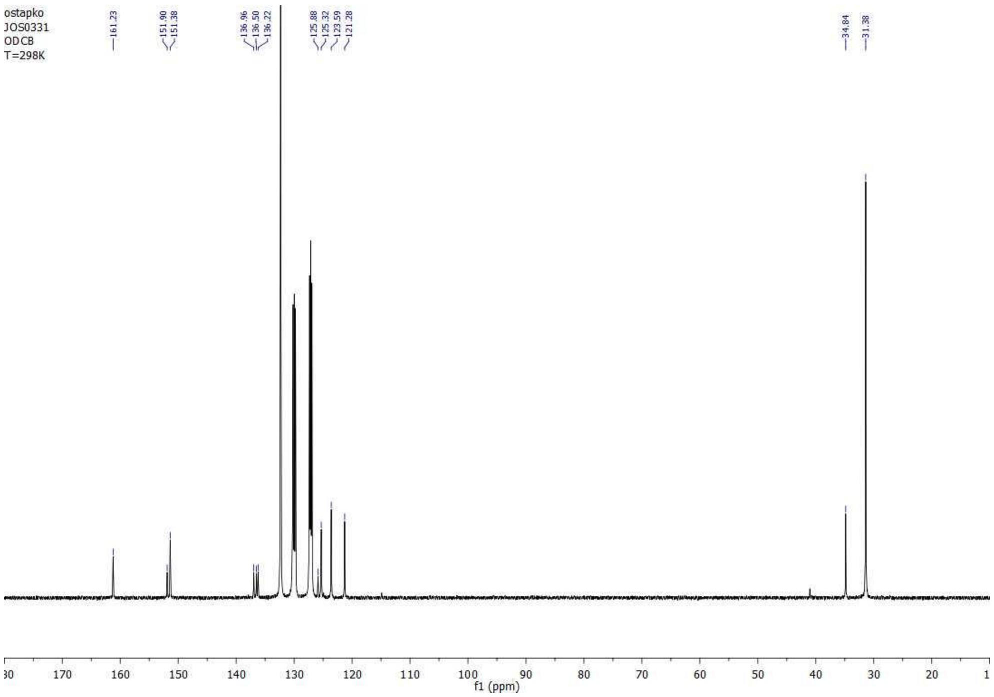

Figure S2. ^{13}C NMR spectrum of **im-ref-1** in *o*-DCB-d_4 (125 MHz, 298 K).

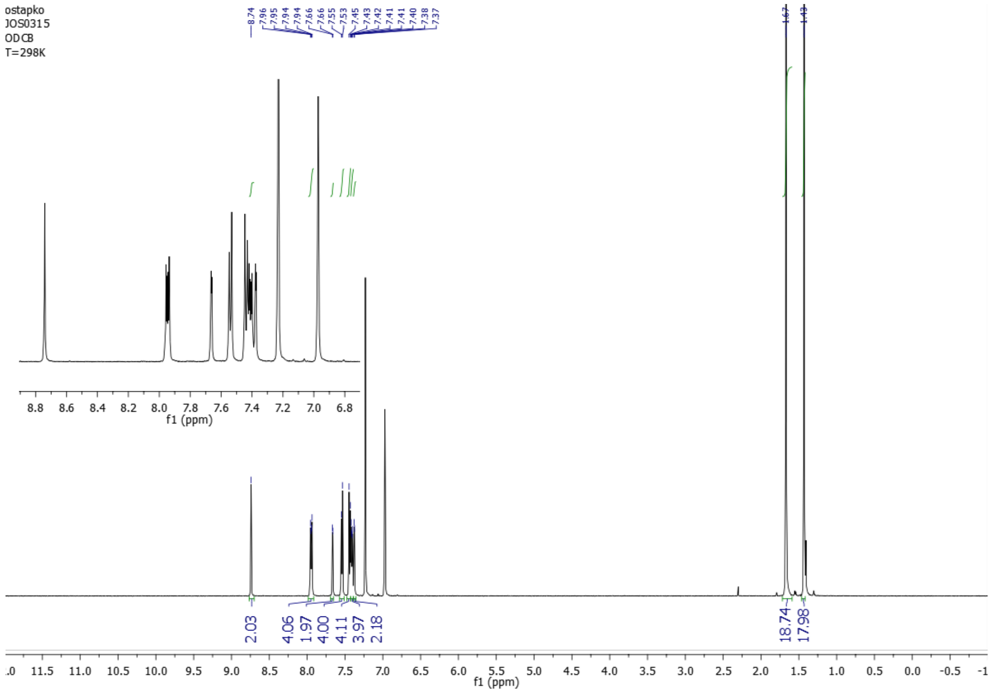

Figure S3. ^{1}H NMR spectrum of **im-ref-2** in *o*-DCB-d_4 (500 MHz, 298 K).

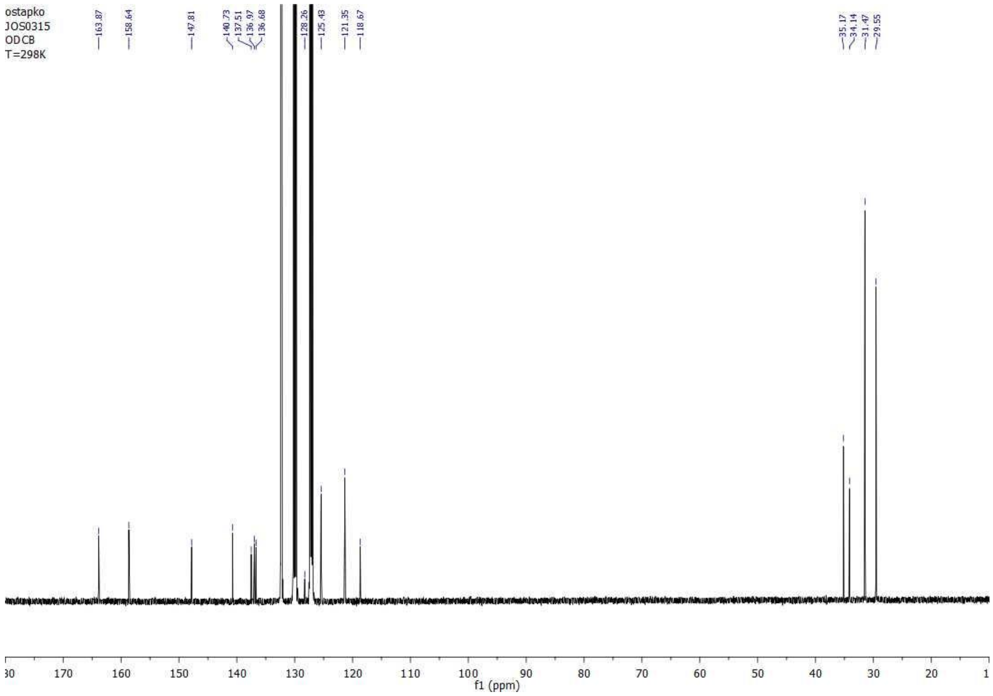

Figure S4. ^{13}C NMR spectrum of **im-ref-2** in *o*-DCB-d_4 (125 MHz, 298 K).

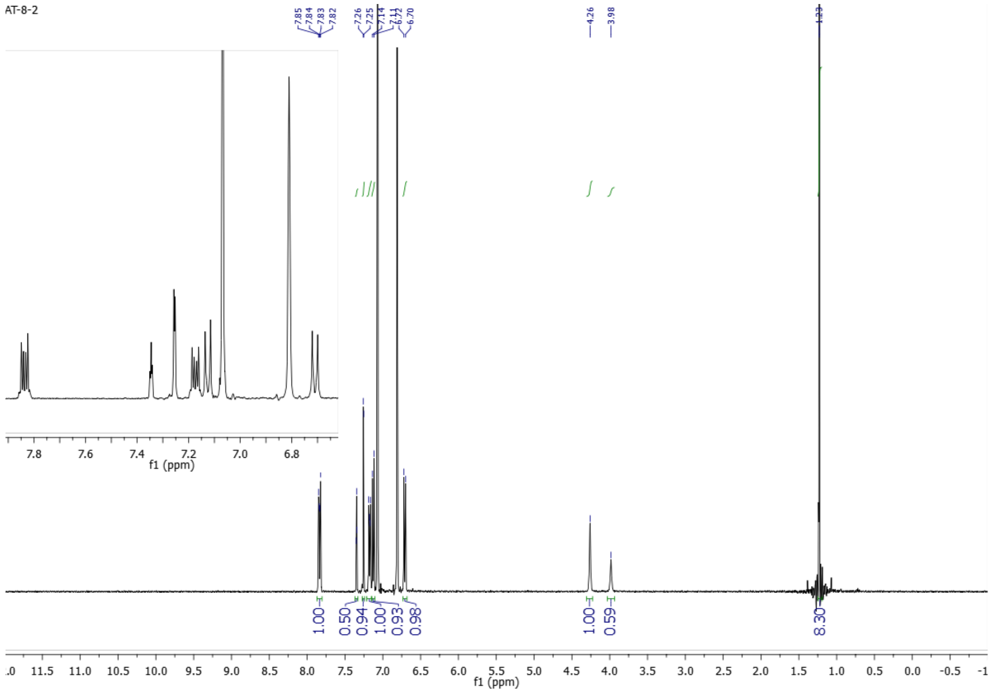

Figure S5. ^{1}H NMR spectrum of **am-ref-3** in *o*-DCB-d$_4$ (400 MHz, 298 K).

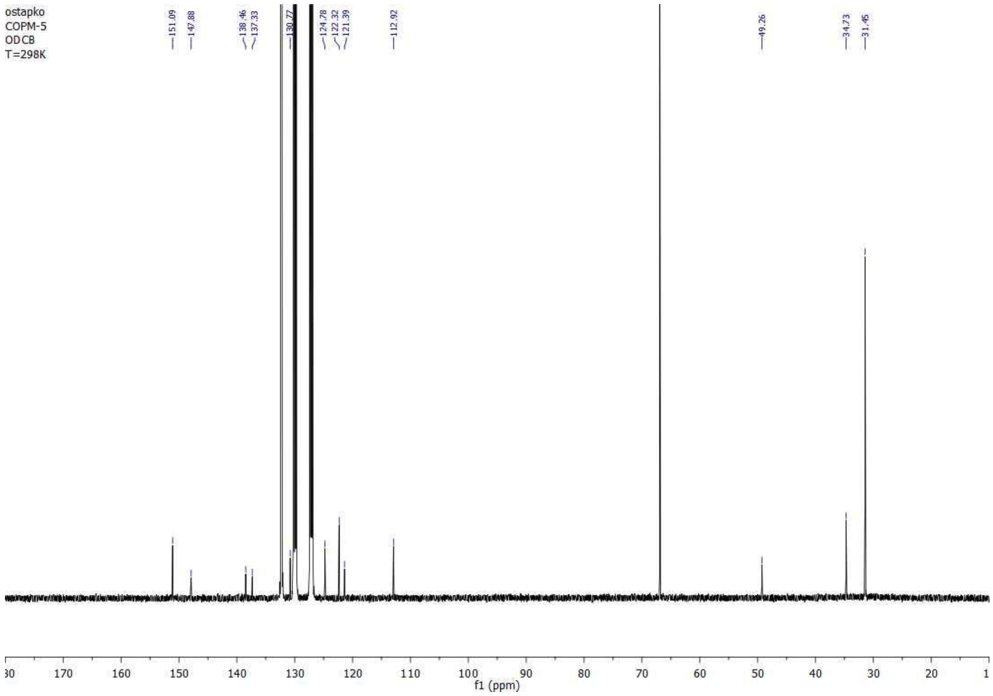

Figure S6. ^{13}C NMR spectrum of **am-ref-3** in *o*-DCB-d$_4$ (125 MHz, 298 K).

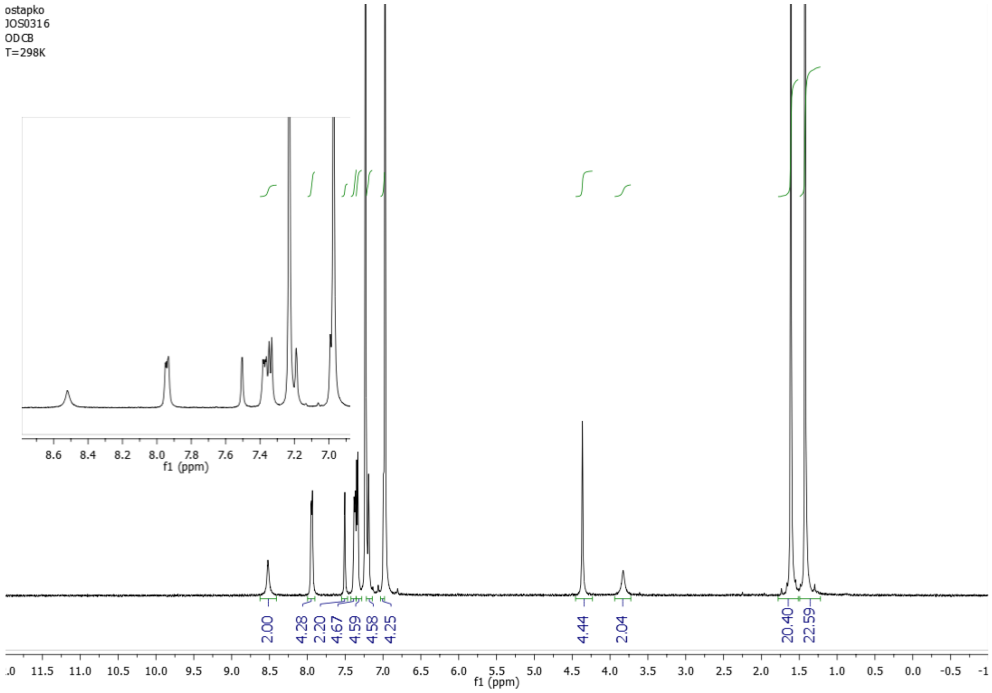

Figure S7. ^{1}H NMR spectrum of **am-ref-4** in *o*-DCB-d$_4$ (500 MHz, 298 K).

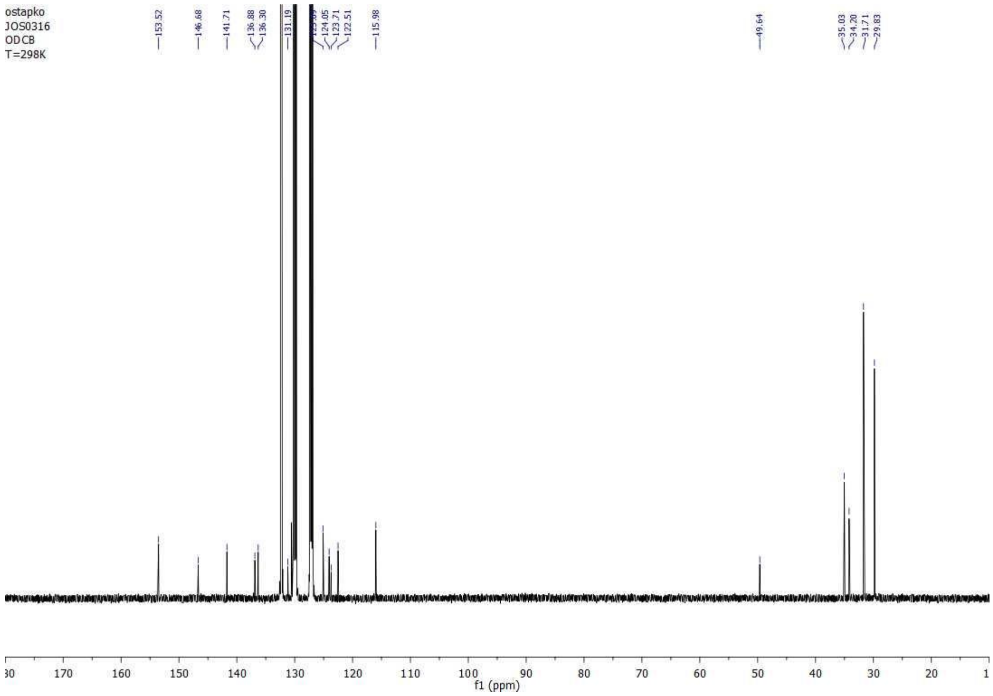

Figure S8. ^{13}C NMR spectrum of **am-ref-4** in *o*-DCB-d$_4$ (125 MHz, 298 K).

4. COF synthesis optimization

Table S1. Optimization of **im-COF-2** synthesis conditions

Entry	Solvent	Temperature/ °C	Appearance/ Comment
1	o-DCB	150	Orange solid
2	o-DCB:n-BuOH (2:1, v/v)	150	Orange solid
3	o-DCB:n-BuOH (4:1, v/v)	150	Orange solid
4	o-DCB:n-BuOH (19:1, v/v)	150	Orange solid
5	o-DCB:n-BuOH (19:1, v/v)	120	Orange solid

Reaction conditions: 0.05 mmol of the aldehyde and 0.15 mmol of amine were placed in to screw-cap vials with a volume of 8 mL, then 2 mL of degassed and anhydrous solvents mixture, and 100 µL of 6M AcOH were added in nitrogen atmosphere in glovebox and reaction mixture was placed in pre-heated aluminum heating blocks outside of the glovebox for 5 days. After that solids were filtrated washed with acetone, CH_2Cl_2 and purified using Soxhlet apparatus with THF for one day, next dried under dynamic vacuum at 120 °C overnight

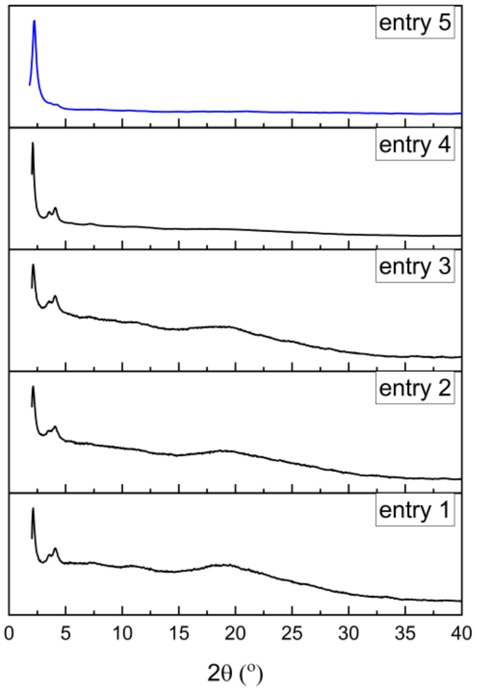

Figure S9. PXRD patterns for **im-COF-2** synthesis optimization. For entries specific conditions see Table S1.

Table S2. Optimization of **im-COP-1** synthesis conditions

Entry	Solvent	Temperature/ °C	Additive	Yield	Appearance
**1**	DMSO	120	-	46.5%	heterogeneous yellow solid
**2**	DMSO	120	aniline	58%	heterogeneous yellow solid
**3**	DMSO	120	6M AcOH	x	x
**4**	DMAC	150	6M AcOH	15%	heterogeneous and patchy, greenish solid
**5***	mesitylene	120	6M AcOH	96%	amorphous, hyaline, yellow solid
**6***	dioxane	120	6M AcOH	91%	amorphous, hyaline, yellow solid
**7**	dioxane:mesitylene (1:1, v/v)	120	6M AcOH	x	x
**8**	dioxane:mesitylene (1:4, v/v)	120	6M AcOH	20%	heterogeneous and patchy, greenish solid
**9***	dioxane:mesitylene (3:2, v/v)	120	6M AcOH	91%	amorphous, hyaline, yellow-orange solid
**10***	dioxane:mesitylene (4:1, v/v)	120	6M AcOH	67.5%	yellow powder
**11**	*o*-DCB	120	6M AcOH	traces	heterogeneous and patchy, greenish solid
**12***	*o*-DCB	120	6M AcOH	46%	amorphous, hyaline, yellow solid
**13**	*o*-DCB:*n*-BuOH (19:1, v/v)	120	6M AcOH	x	X
**14**	*o*-DCB:*n*-BuOH (2:1, v/v)	120	6M AcOH	23%	heterogeneous yellow solid
**15**	*o*-DCB:*n*-BuOH (4:1, v/v)	120	Glacial AcOH	83%	yellow solid
**16**	*o*-DCB:*n*-BuOH (4:1, v/v)	120	6M AcOH	78%	yellow solid
**17**	*o*-DCB:*n*-BuOH (4:1, v/v)	150	6M AcOH	74.5%	yellow solid
**18***	*o*-DCB:*n*-BuOH (4:1, v/v)	150	6M AcOH	89%	yellow solid

Reaction conditions: 0.05 mmol of the aldehyde and 0.15 mmol of amine were placed in to screw-cap vials with a volume of 8 mL, then 2 mL of degassed and anhydrous solvents mixture, and additives were added in nitrogen atmosphere in glovebox and reaction mixture was placed in pre-heated aluminum heating blocks outside of the glovebox for 7 days. After that solids were filtrated washed with acetone, CH_2Cl_2, and purified using Soxhlet apparatus with THF for one day, next dried under vacuum at 120 °C overnight

*1 mL of solvents mixture was used

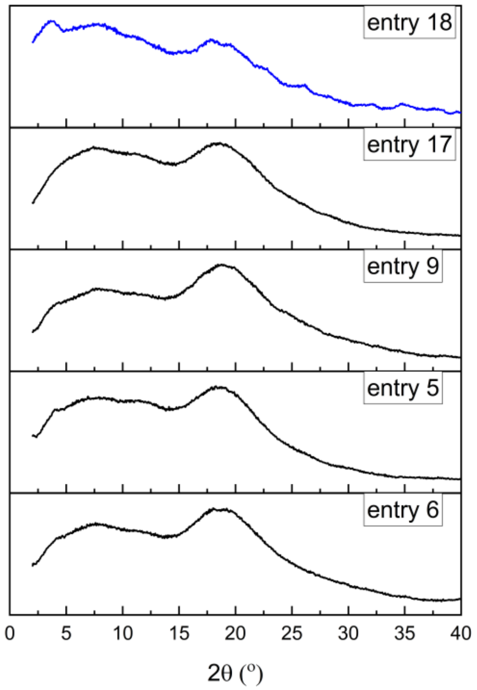

Figure S10. PXRD patterns for **im-COP-1** synthesis optimization. For entries specific conditions see Table S2.

Table S3. Optimization of synthesis of **am-COP-3**.

entry	Starting material	solvent	Reducing agent	Additive	Emission properties
**1***	im-COP-1	DCE	$NaBH(OAc)_3$ 40 equiv.	-	No emission
**2***	im-COP-1	THF	$NaBH(OAc)_3$ 40 equiv.	-	No emission
**3***	im-COP-1	MeOH	$NaBH(OAc)_3$ 40 equiv.	-	No emission
**4***	im-COP-1	Dioxane	$NaBH(OAc)_3$ 40 equiv.	-	No emission
**5***	im-COP-1	THF	$NaBH_3CN$ 40 equiv.	-	No emission
**6***	im-COP-1	THF	$NaBH_4$ 40 equiv.	AcOH conc., 8 equiv.	Strong emission
**7***	im-COP-1	THF	$NaBH_4$ 40 equiv.	AcOH conc., 8 equiv.	Strong emission
**8***	im-COP-1	EtOH	$NaBH_4$ 40 equiv.	AcOH conc., 8 equiv.	No emission
**9**	im-COP-1	THF:EtOH (3:1, v/v)	$NaBH_4$ 40 equiv.	-	Strong emission
**10**	im-COP-1	THF:EtOH (3:1, v/v)	$NaBH_4$ 40 equiv.	Benzoic acid 8 equiv.	Strong emission

Reaction conditions: 0.020 mmol of COP was placed in a vial, together with appropriate reducing agent and additive. Then 4mL of solvent (or mixture of solvents) was added and reaction mixture was stirred overnight at room temperature. Then solid was filtered washed with MeOH, CH_2Cl_2, and purified using Soxhlet apparatus with MeOH for one day, next dried under dynamic vacuum at 120 °C overnight.
* 3 mL of solvent was used

Table S4. Optimization of synthesis of **am-COP-4**.

entry	Starting material	solvent	Reducing agent	Additive	Observations
**1***	im-COF-2	MeOH	$NaBH_4$ 40 equiv.	p-phthalic acid 10 equiv.	no emission
**2**	im-COF-2	THF:EtOH (3:1, v/v)	$NaBH_4$ 40 equiv.	AcOH conc., 8 equiv.	strong emission
**3**	im-COF-2	THF:EtOH (3:1, v/v)	$NaBH_4$ 40 equiv.	benzoic acid 8 equiv.	strong emission
**4**	im-COF-2	THF:EtOH (3:1, v/v)	$NaBH_4$ 40 equiv.	p-phthalic acid 8 equiv.	strong emission
**5**	im-COF-2	THF:EtOH (3:1, v/v)	$NaBH_3CN$ 40 equiv.	benzoic acid 8 equiv.	strong emission
**6**	im-COF-2	Mes:Dioxane (2:1, v/v)	HCOOH (98%) 20 equiv.		strong emission
**7**	im-COF-2	Mes:Dioxane (2:1, v/v)	HCOOH (98%) 60 equiv.		decomposition
**8**	im-COF-2	THF:EtOH (3:1, v/v)	HCOOH (5 M) 20 equiv.	benzoic acid 8 equiv.	decomposition

Reaction conditions: 0.020 mmol of COF was placed in a vial, together with appropriate reducing agent and additive. Then 4mL of solvent (or mixture of solvents) was added and reaction mixture was stirred overnight at room temperature. Then solid was filtered washed with MeOH, CH_2Cl_2, and purified using Soxhlet apparatus with MeOH for one day, next dried under dynamic vacuum at 120 °C overnight.
*13 mL of solvent was used

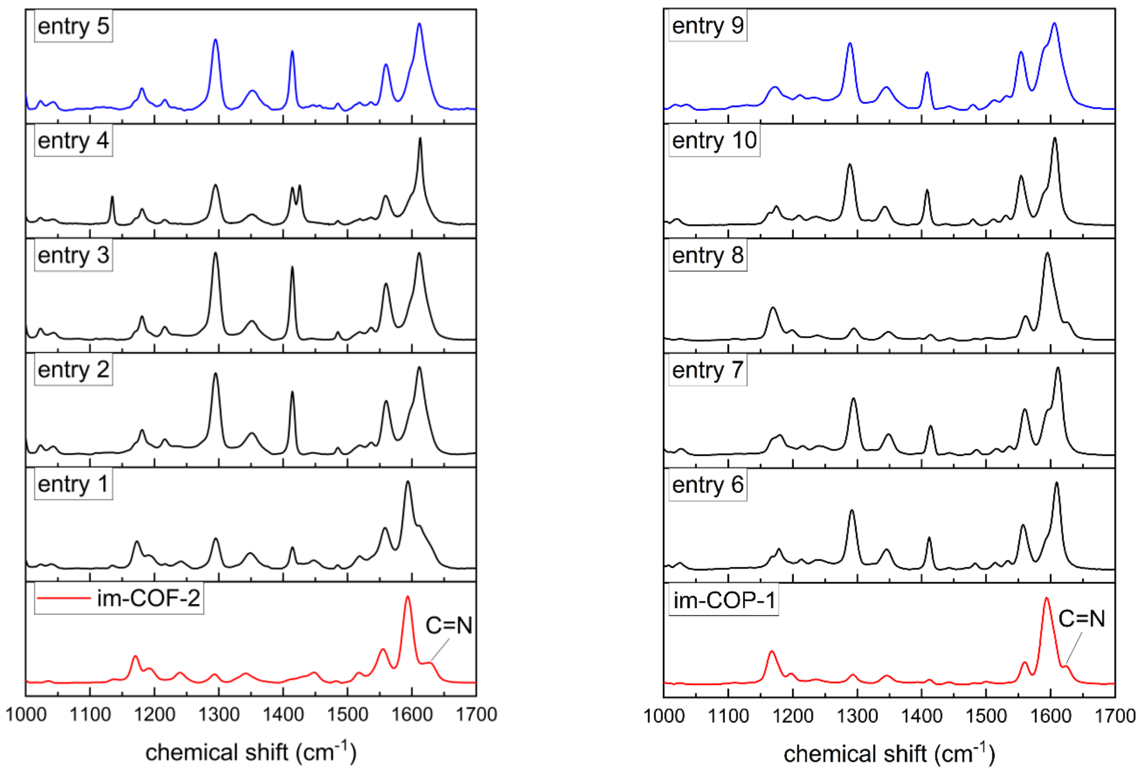

Figure S11. Raman spectra: optimization of **am-COP-4** (left) and **am-COP-3** (right) synthesis. For entries conditions assignment see Table S4 and Table S3, respectively.

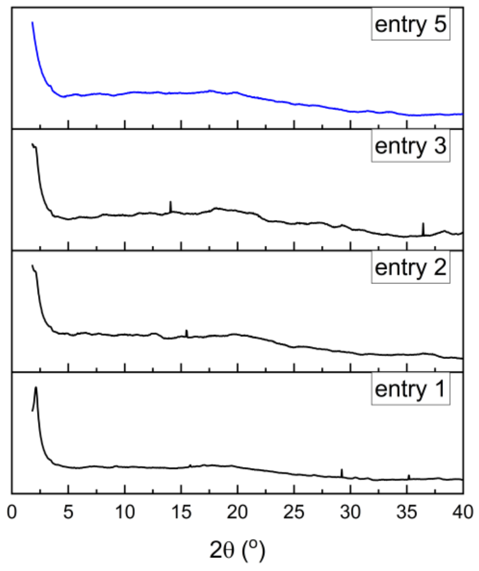

Figure S12. PXRD patterns. Optimization of **am-COP-4** synthesis. For entries conditions assignment see Table S4.

5. Raman and Fourier transformed IR spectroscopy

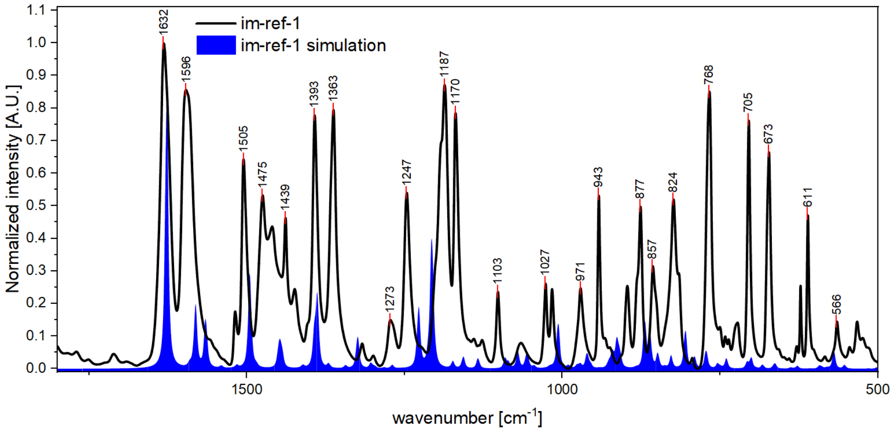

Figure S13. FT-IR spectrum of **im-ref-1** and its comparison with simulated spectrum.

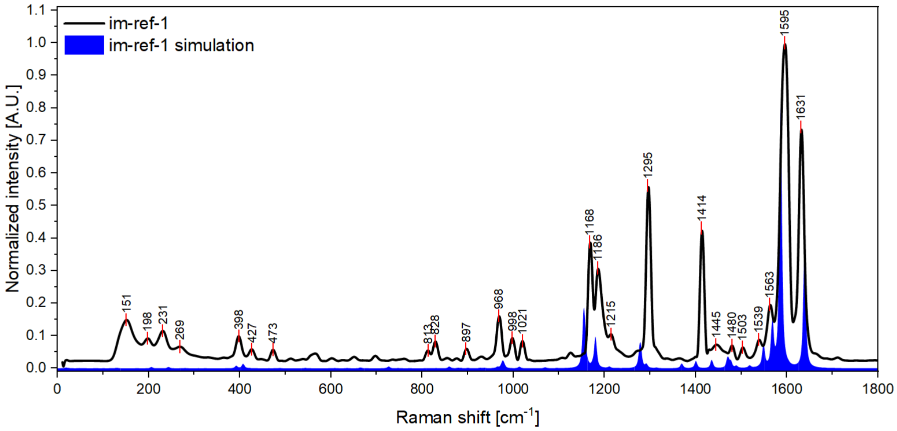

Figure S14. Raman spectrum of **im-ref-1** and its comparison with simulated spectrum.

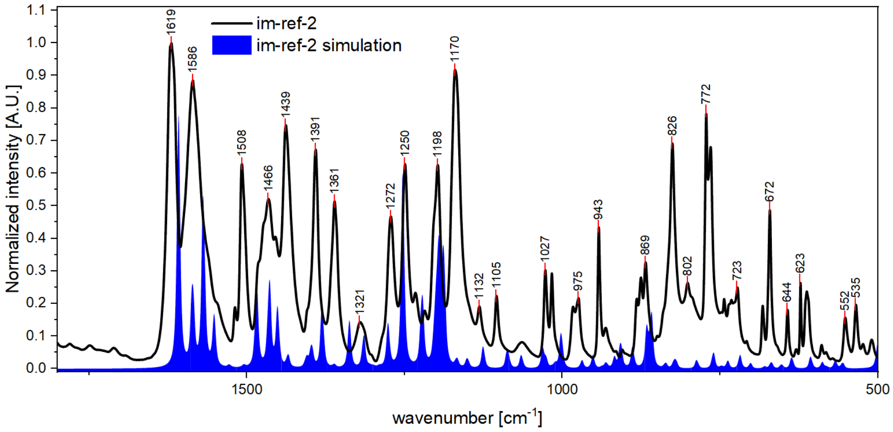

Figure S15. FT-IR spectrum of **im-ref-2** and its comparison with simulated spectrum.

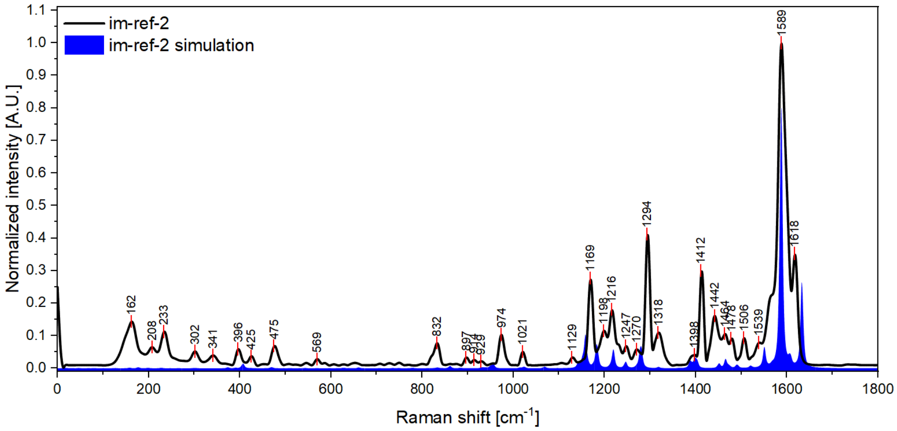

Figure S16. Raman spectrum of **im-ref-2** and its comparison with simulated spectrum.

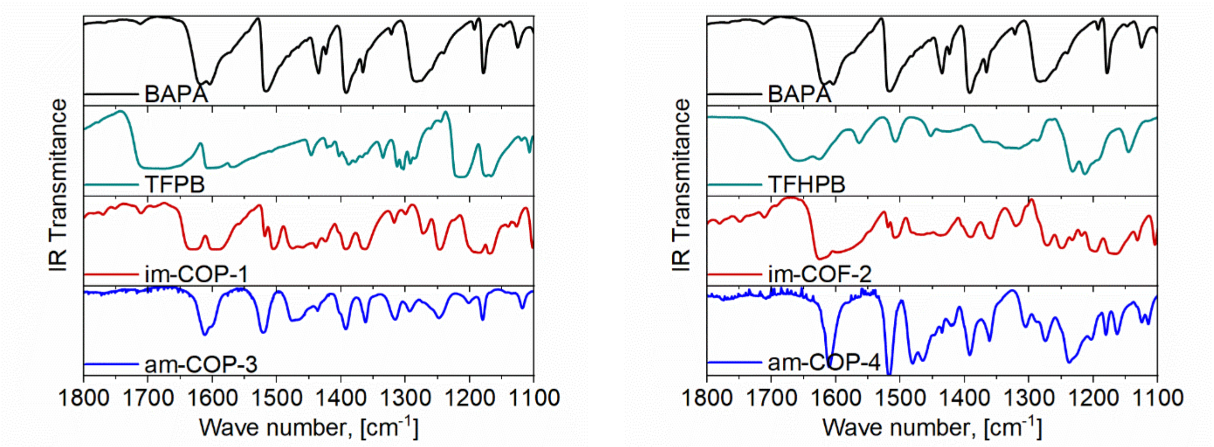

Figure S17. Left: FT-IR spectra of **im-COF-1**, substrates used for its synthesis (**BAPA** and **TFPB**) and product of its reduction, **am-COF-3**. Right: FT-IR spectra of **im-COF-2**, substrates used for its synthesis (**BAPA** and **TFHPB**) and product of its reduction, **am-COF-3**.

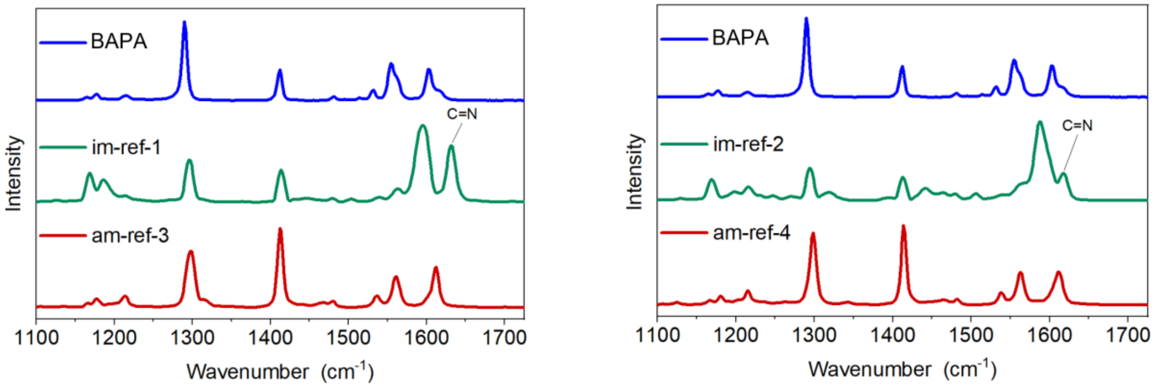

Figure S18. Left: Raman spectra of **im-ref-1**, substrates used for its synthesis (**BAPA**) and product of its reduction, **am-ref-3**. Right: Raman spectra of **im-ref-2**, substrates used for its synthesis (**BAPA**) and product of its reduction, **am-ref-4**.

6. PXRD

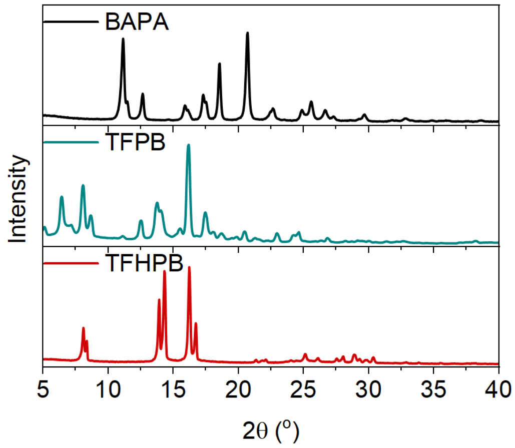

Figure S19. PXRD patterns of the substates used for the synthesis of **im-COF-1** (BAPA, TFPB), and **im-COF-2** (BAPA, TFHPB).

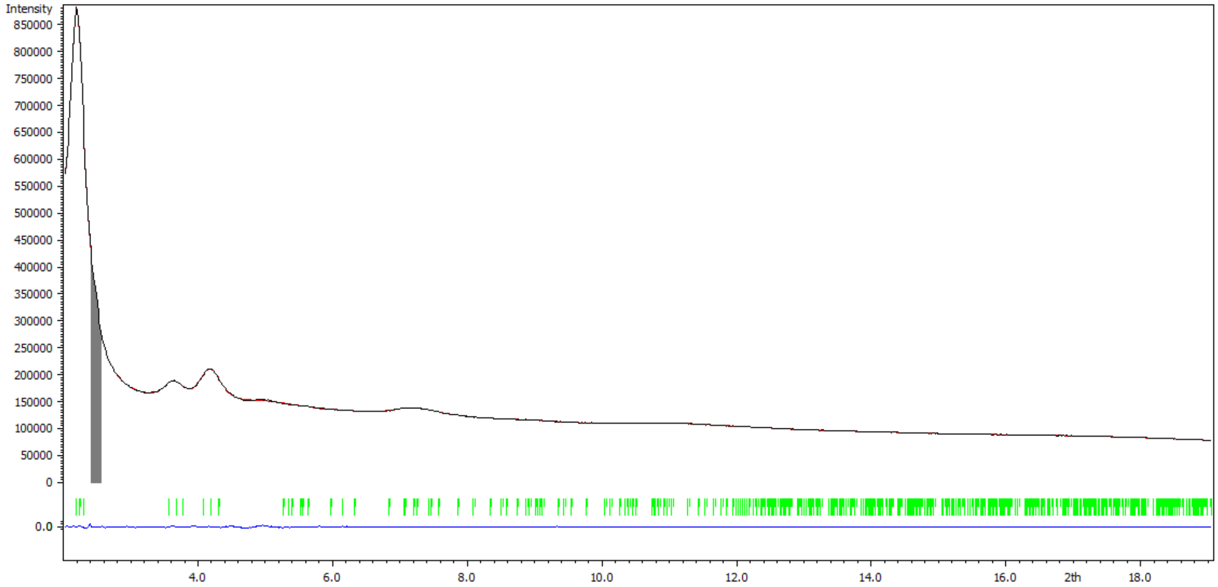

Figure S20. La Bail refined PXRD pattern of **im-COF-2**. Experimental PXRD pattern (black), refined PXRD pattern (red), difference (blue), Bragg positions (green). Excluded region due to the apparatus signal (grey)

Table S5. DFT calculated lattice parameters for **im-COF-2** AA and AB stacking models and refined lattice parameters.

Stacking mode	Calculated model	Refined parameters
**AA**	a=52.1255 Å<br>b=52.137 Å<br>c=8.12646 Å<br>α=90.8501°<br>β=91.2884°<br>γ=60.074°	a=50.86 Å<br>b=52.37 Å<br>c=7.60 Å<br>α=89.04°<br>β=91.67°<br>γ=63.14°<br><br>R_{wp} = 0.34, R_p = 0.25.
**AB**	a=52.177 Å<br>b=52.1748 Å<br>c=8.0445 Å<br>α=89.9709°<br>β=89.9804°<br>γ=59.9882°	

7. SEM

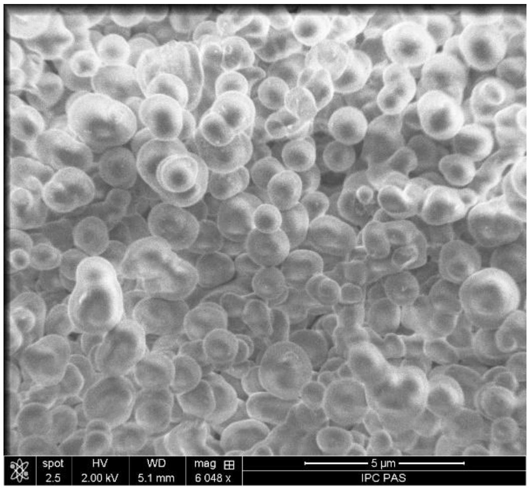

im-COP-1

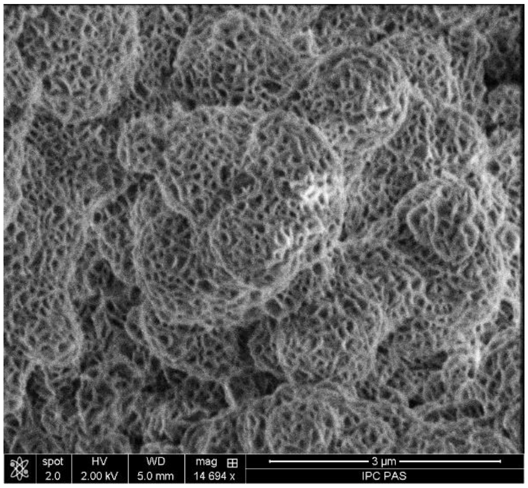

im-COF-2

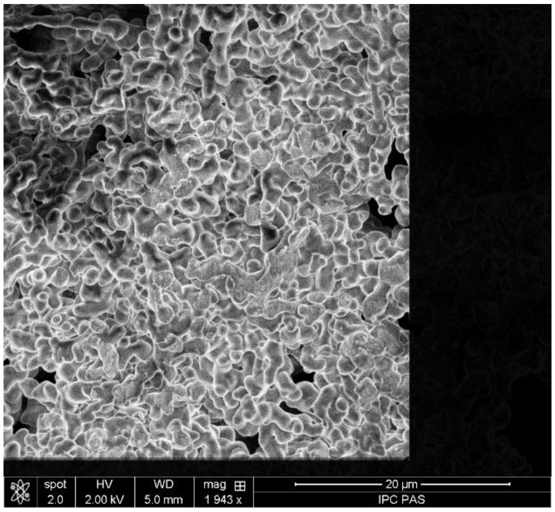

am-COP-3

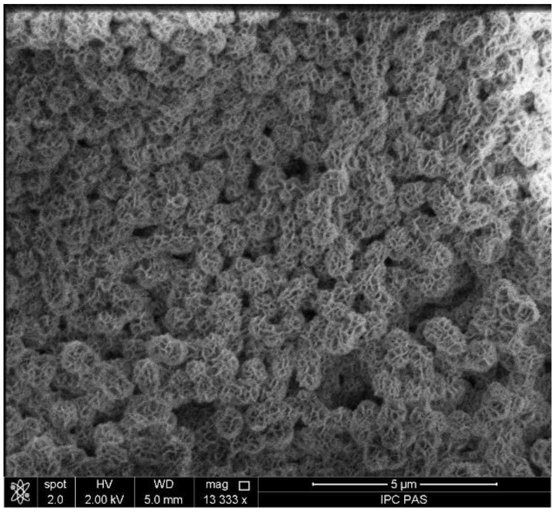

am-COP-4

Figure S21. SEM images of synthesized COF(P)s. The black area for the **am-COP-3** images comes from the drift correction.

8. TEM

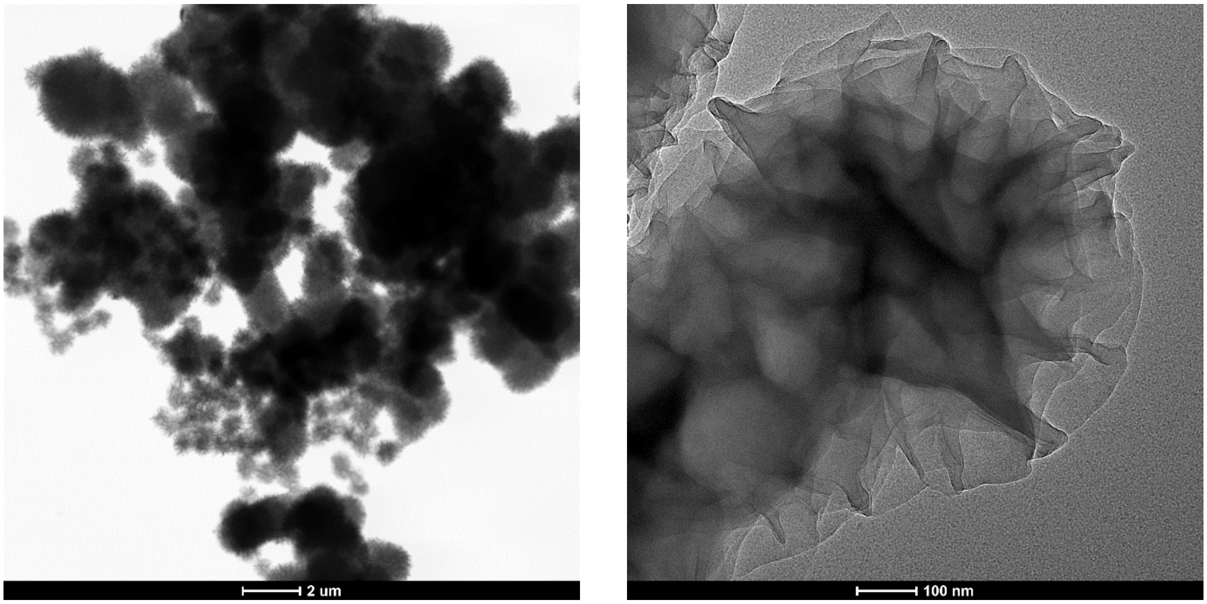

Figure S22. TEM images of **im-COF-2**.

9. DSC-TGA

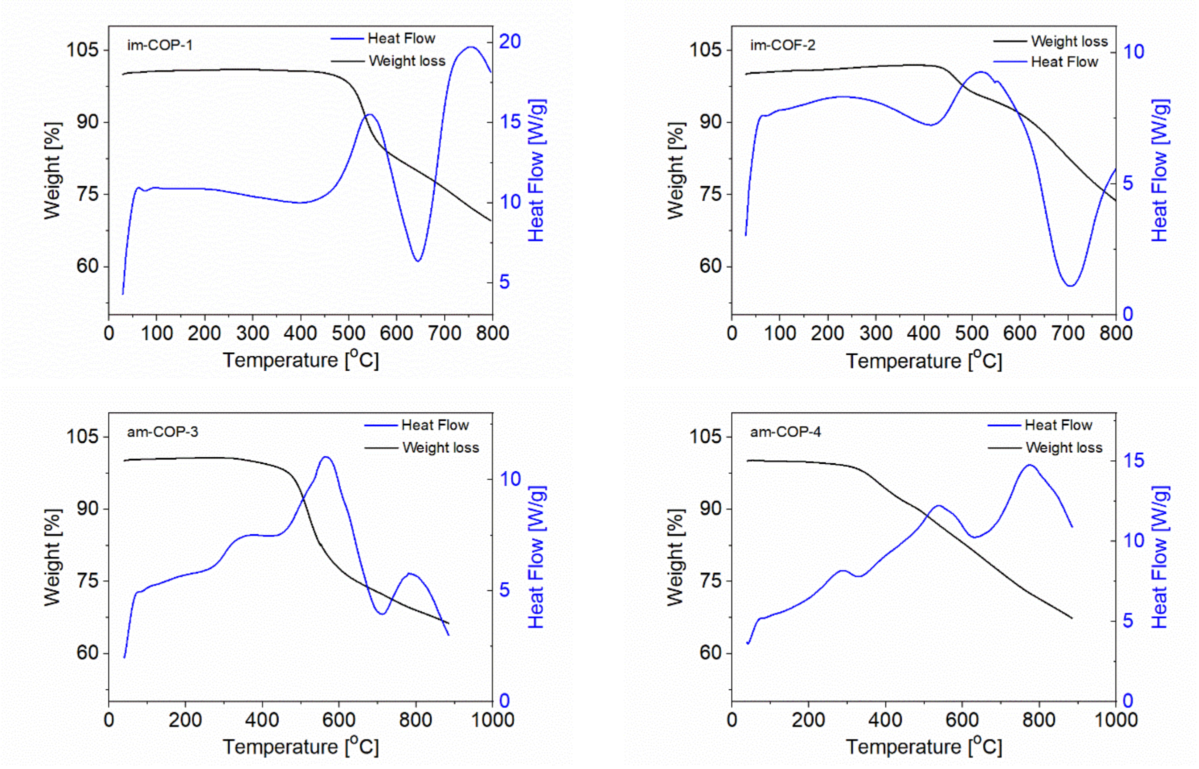

Figure S23. DSC-TGA graph representing thermal stability of **im-COP-1** (top left), **im-COF-2** (top right), **am-COP-3** (down left), and **am-COP-4** (down right): weight loss (black), heat flow (blue).

10. DFT

Table S6. Frontier orbitals distribution calculated for relaxed ground and excited states of models of **im-ref-1** and **am-ref-3**.

	model for **im-ref-1**		model for **am-ref-3**	
	S_0	S_1	S_0	S_1
**LUMO+2**	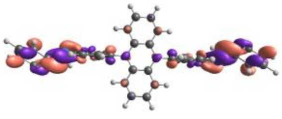 -0.69 eV	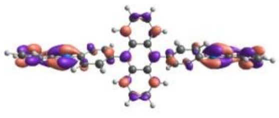 -0.66 eV	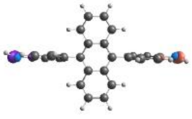 0.29 eV	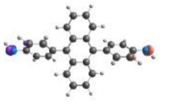 0.29 eV
**LUMO+1**	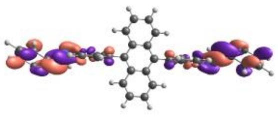 -0.69 eV	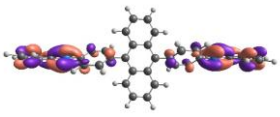 -0.74 eV	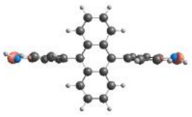 0.27 eV	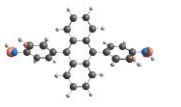 0.27 eV
**LUMO**	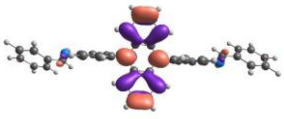 -0.79 eV	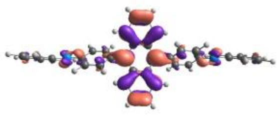 -1.24 eV	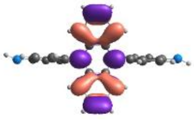 -0.62 eV	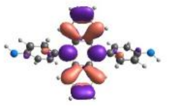 -0.95 eV
**HOMO**	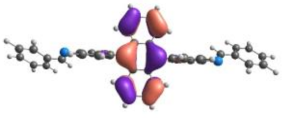 -6.38 eV	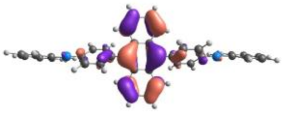 -6.12 eV	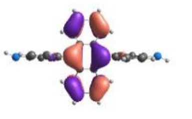 -6.40 eV	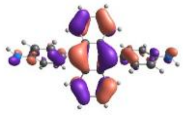 -5.87 eV
**HOMO-1**	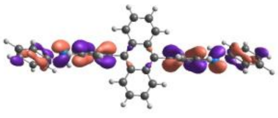 -7.58 eV	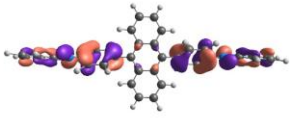 -7.55 eV	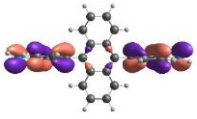 -7.11 eV	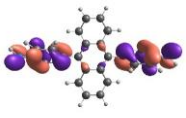 -7.09 eV
**HOMO-2**	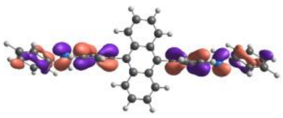 -7.59 eV	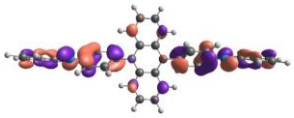 -7.65 eV	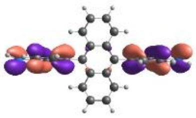 -7.12 eV	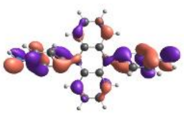 -7.24 eV

Table S6. Frontier orbitals distribution for ground and excited states of model of **im-ref-2**.

	model of **im-ref-2**		
	S_0	S_1	S_1 ESIPT
**LUMO+2**	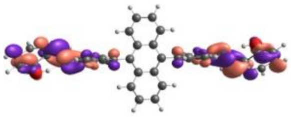	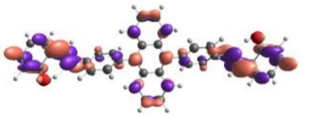	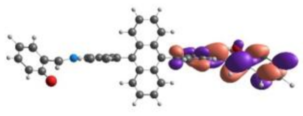
	-0.86 eV	-0.82 eV	-1.03 eV
**LUMO+1**	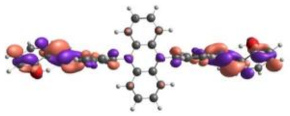	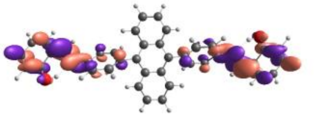	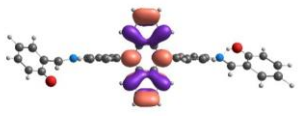
	-0.86 eV	-0.94 eV	-1.33 eV
**LUMO**	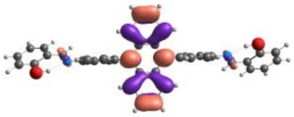	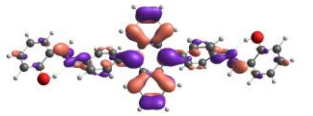	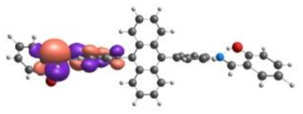
	-0.94 eV	-1.46 eV	-2.50 eV
**HOMO**	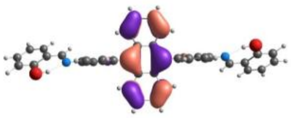	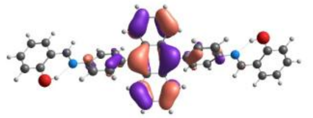	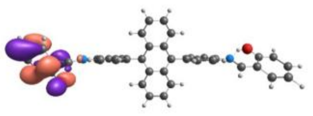
	-6.74 eV	-6.29 eV	-5.61 eV
**HOMO-1**	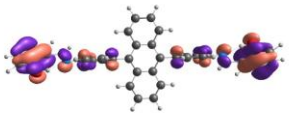	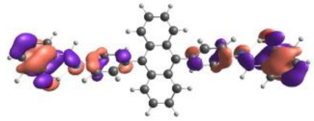	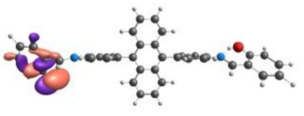
	-7.56 eV	-7.52 eV	-6.97 eV
**HOMO-2**	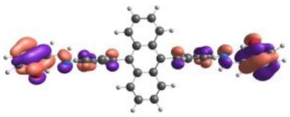	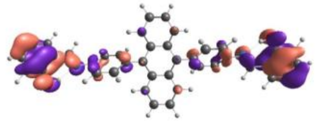	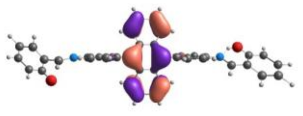
	-7.56 eV	-7.58 eV	-7.14 eV

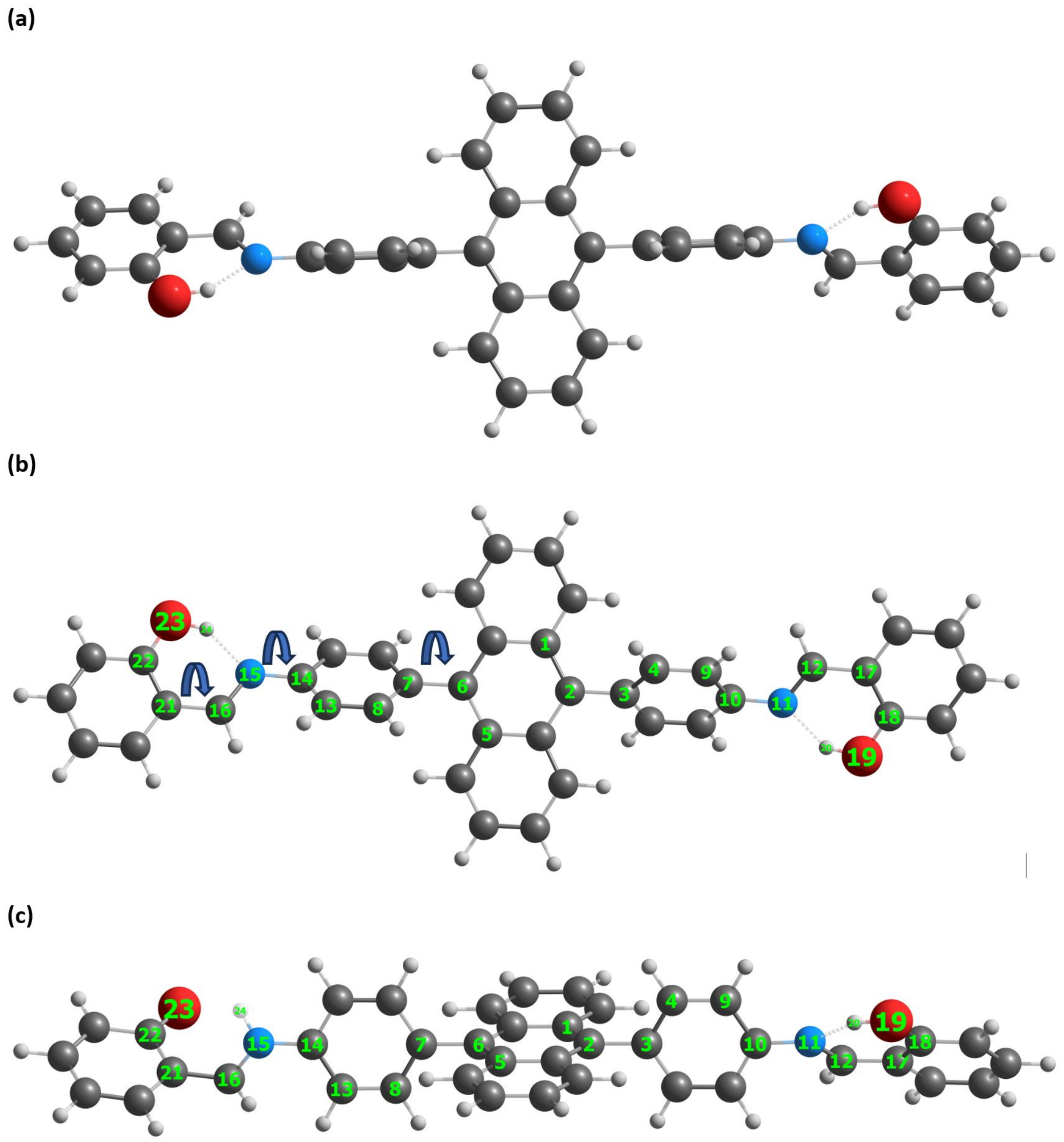

Figure S24. Optimized geometry of **am-ref-2** model in the S_0 electronic state (a), in the S_1 electronic state at the local energy minimum near the S_0 geometry (b), and at the global S_1 minimum with a single ESIPT (c).

Table S8. Selected structural and spectroscopic parameters of the **im-ref-2** model geometries obtained from simulations. Energies in nm are given with respect to the vertical transitions between the ground and excited states of the molecule in the given geometry.

model of im-ref-2	Vertical transition energy/ nm	Dihedral angle / deg.					
		(1-2-3-4)	(5-6-7-8)	(9-10-11-12)	(13-14-15-16)	(11-12-17-18)	(15-16-19-20)
**S_0**	357	88	88	41	41	1	1
**S_1 local minimum**	451	56	56	34	34	1	1
**S_1 global minimum (single ESIPT)**	5168	89	89	12	12	79	79

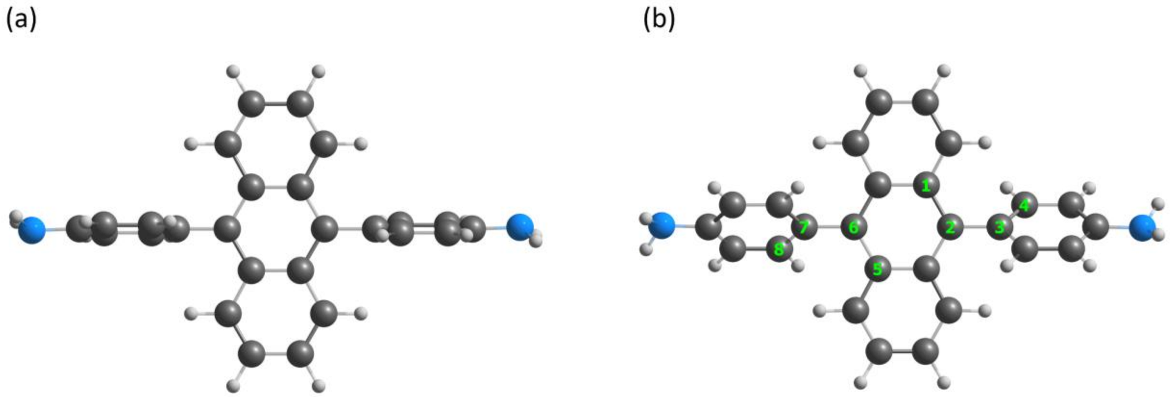

Figure S25. Optimized geometry of **am-ref-3** model in the S_0 electronic state (a) and in the S_1 electronic state (b).

Table S8. Selected structural and spectroscopic parameters of the **am-ref-3** model geometries obtained from simulations. Energies in nm are given with respect to the vertical transitions between the ground and excited states of the molecule in the given geometry.

model of am-ref-3	Vertical transition / nm	Dihedral angel (1-2-3-4) and (5-6-7-8) / deg.
**S_0**	356	85
**S_1**	446	58

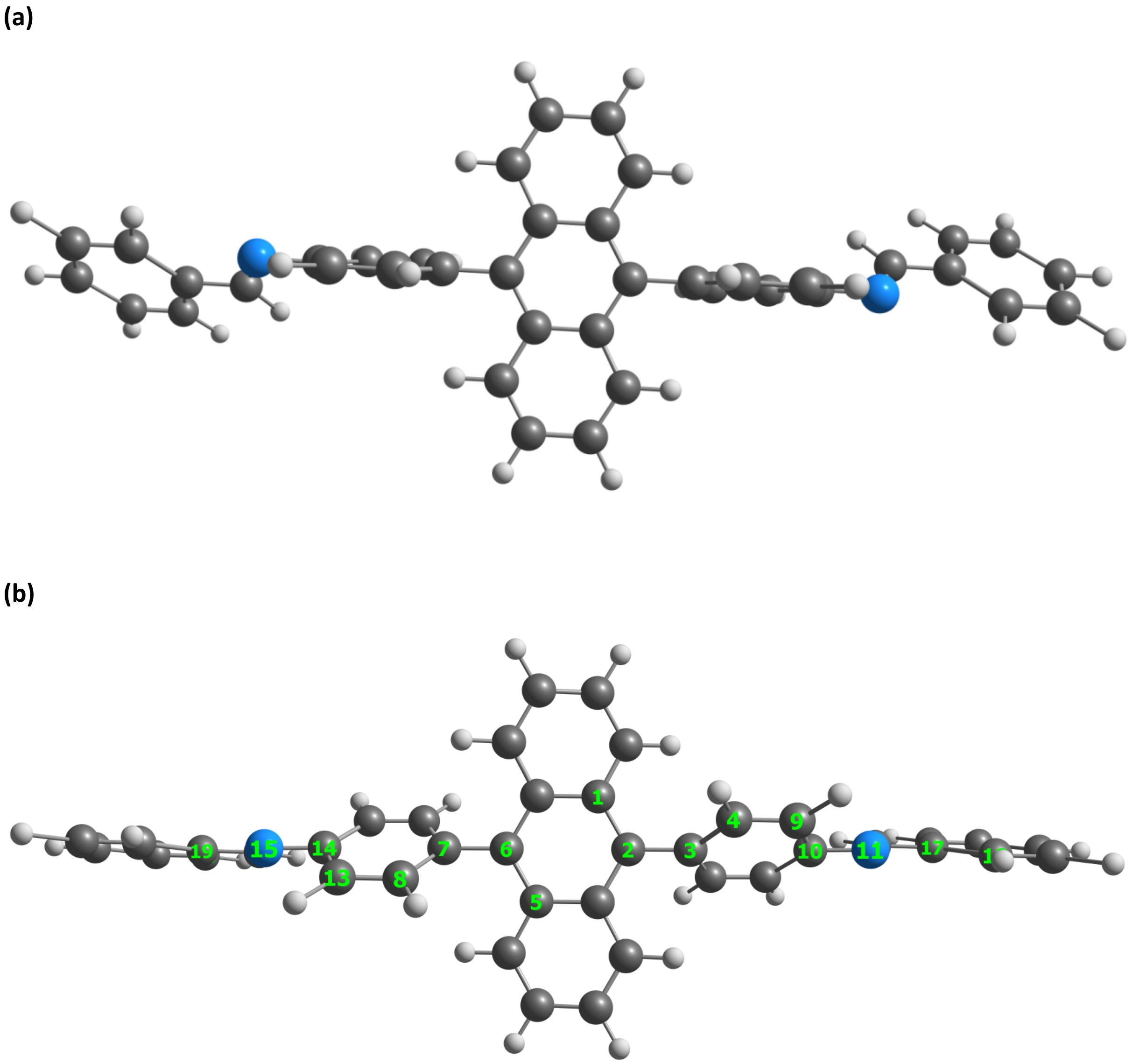

Figure S26. Optimized geometry of **im-ref-1** model in the S_0 electronic state (a) and in the S_1 electronic state (b).

Table S10. Selected structural and spectroscopic parameters of the **im-ref-1** model geometries obtained from simulations. Energies in nm are given with respect to the vertical transitions between the ground and excited states of the molecule in the given geometry.

model of im-ref-1	Vertical transition / nm	Dihedral angel (1-2-3-4) and (5-6-7-8) / deg.	Dihedral angel (9-10-11-12) and (13-14-15-16) / deg.	Dihedral angel (11-12-17-18) and (15-16-19-20) /deg.
**S_0**	357	87	44	1
**S_1**	448	58	18	2

11. Spectroscopy

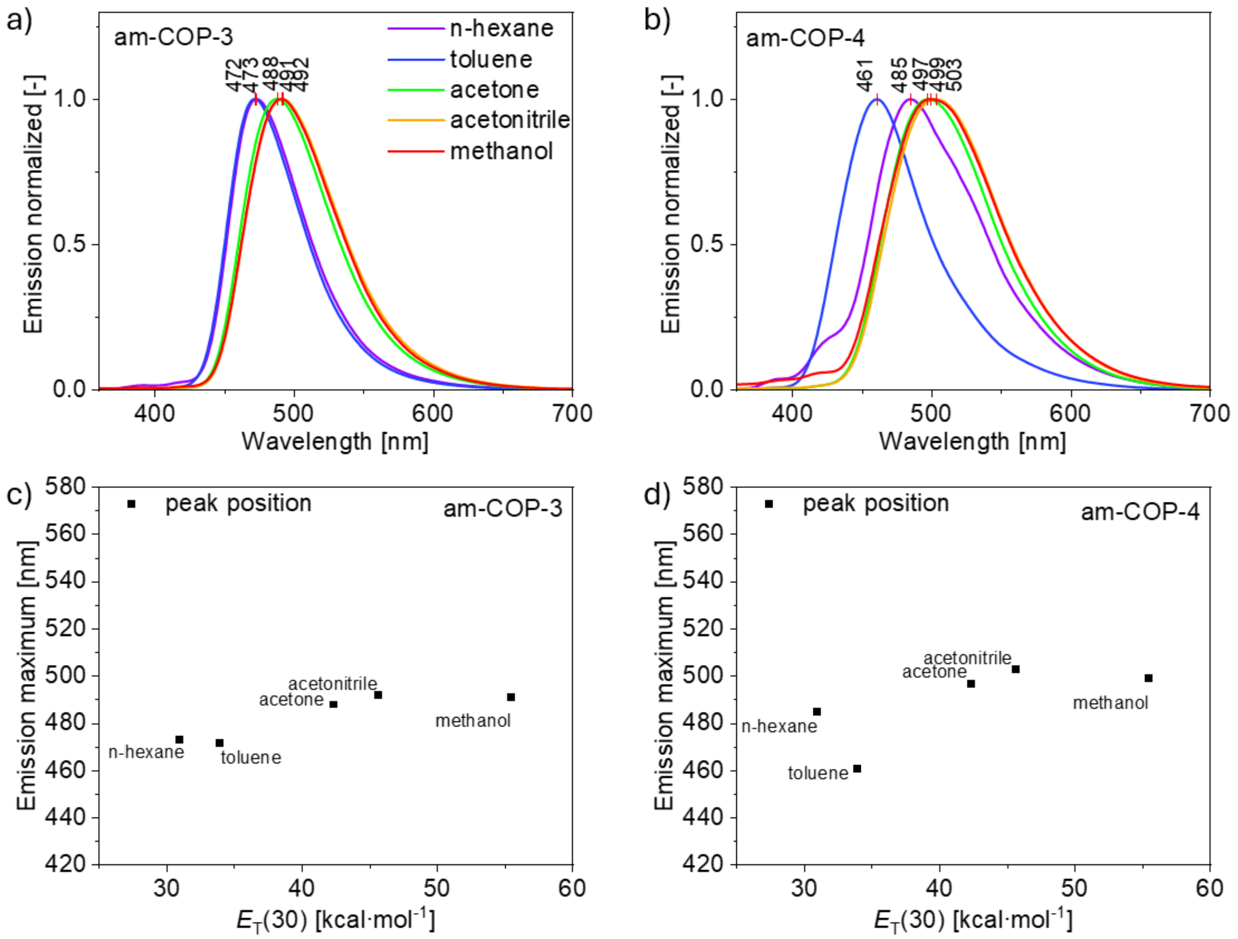

Figure S27. Normalized emission spectra of a) **am-COP-3** b) and **am-COP-4** measured in various solvents and dependence of the emission maximum on the ET(30) solvents' polarity scale for c) **am-COP-3** and d) **am-COP-4**. These plots visually demonstrate the shift in emission maximum with increasing solvent polarity. The spectra are normalized to their maximum intensity, illustrating the solvatochromic behavior of the materials. Solvents are indicated by color: n-hexane (purple), toluene (blue), acetone (green), acetonitrile (orange), and methanol (red).

Table S11. Fluorescence lifetimes of selected references and COFs measured in different solvents.

	Fluorescence lifetime, ns			
	**am-COP-3**	**am-COP-4**	**am-ref-3**	**am-ref-4**
n-hexane	2.3	2.4	1.94	2.11
toluene	1.5	1.8	2.12	2.25
acetone	1.6	2.2	4.23	5.28
acetonitrile	1.8	2.3	5.45	5.97
methanol	1.9	2.1	4.46	3.49

Table S12 Measured fluorescence quantum yields (QY, %) of reference compounds in different solvents.

	n-hexane	toluene	acetone	acetonitrile	methanol
**im-ref-1**	0.14±0.03	0.21±0.03	0.25±0.04	0.67±0.07	1.18±0.12
**im-ref-2**	0.10±0.01	0.16±0.05	0.17±0.02	0.56±0.04	1.2±0.06
**am-ref-3**	69.51±5.50	74.82±5.83	48.35±4.05	43.94±2.91	17.5±5.28
**am-ref-4**	77.96±7.23	75.18±6.06	50.93±1.34	38.62±0.68	22.3±0.39

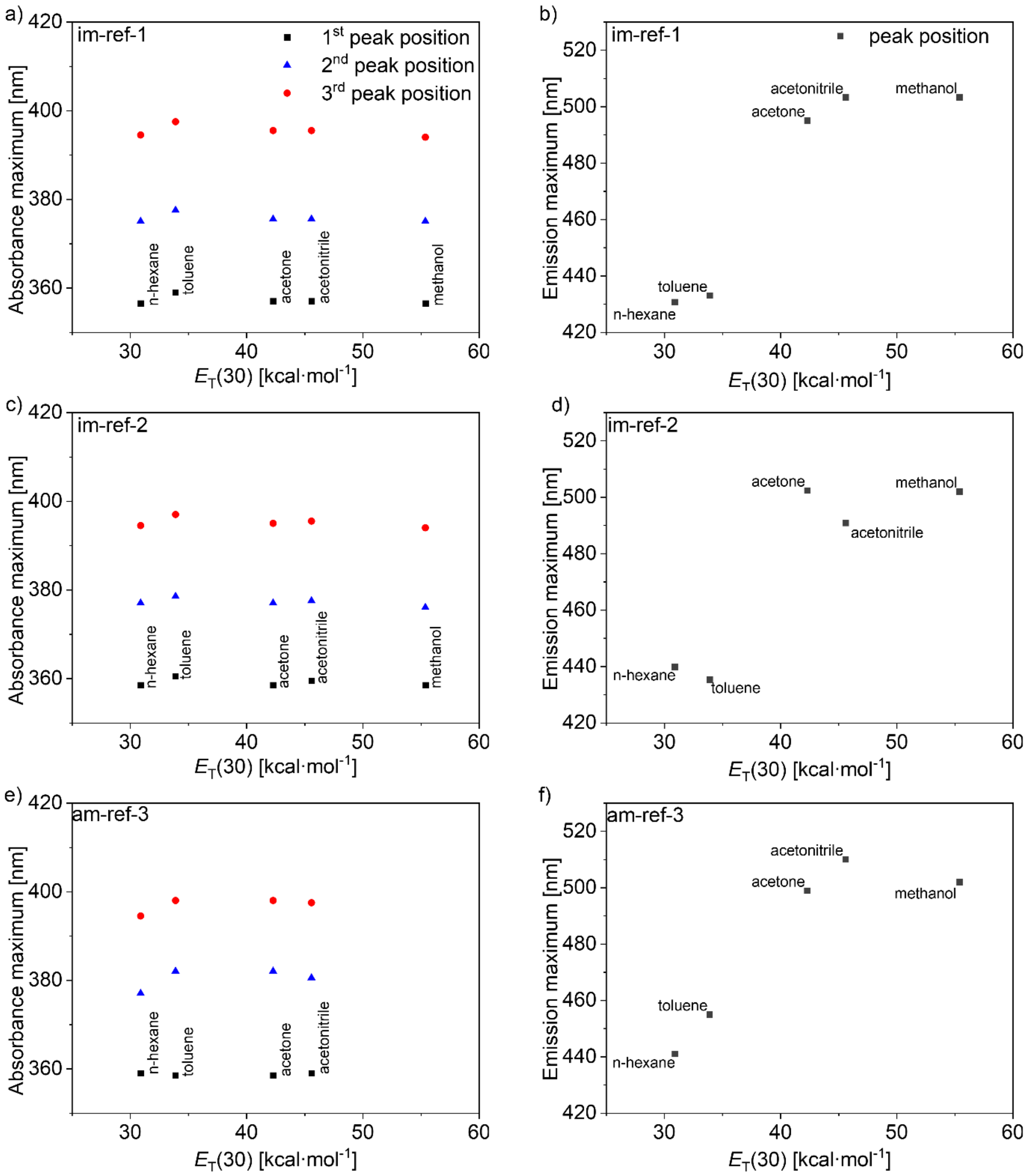

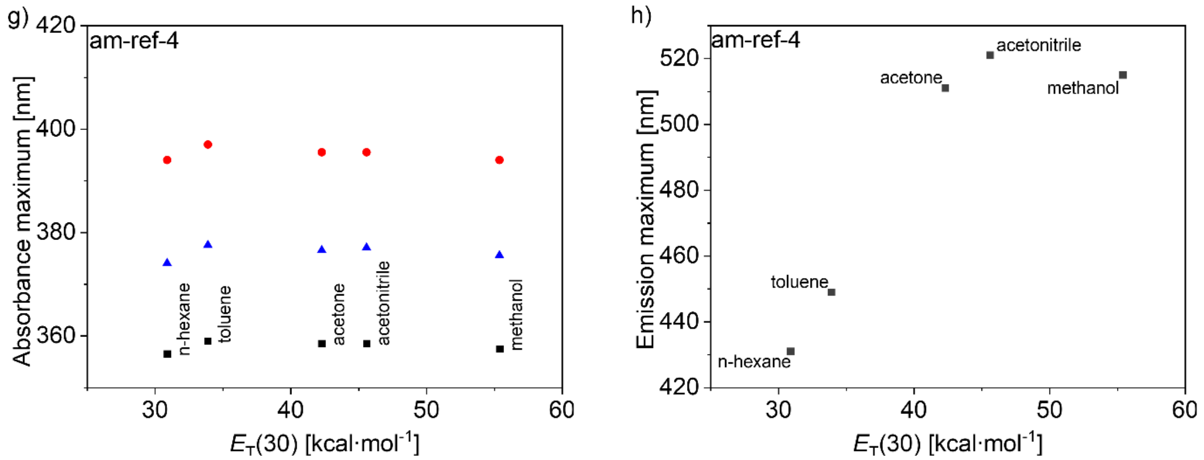

Figure S28 a), c), e), g) Absorbance maxima and b), d), f), h) emission maximum of reference **imines** a)-d) and **amines** e)-h) in relation to $E_T(30)$ solvents' polarity.

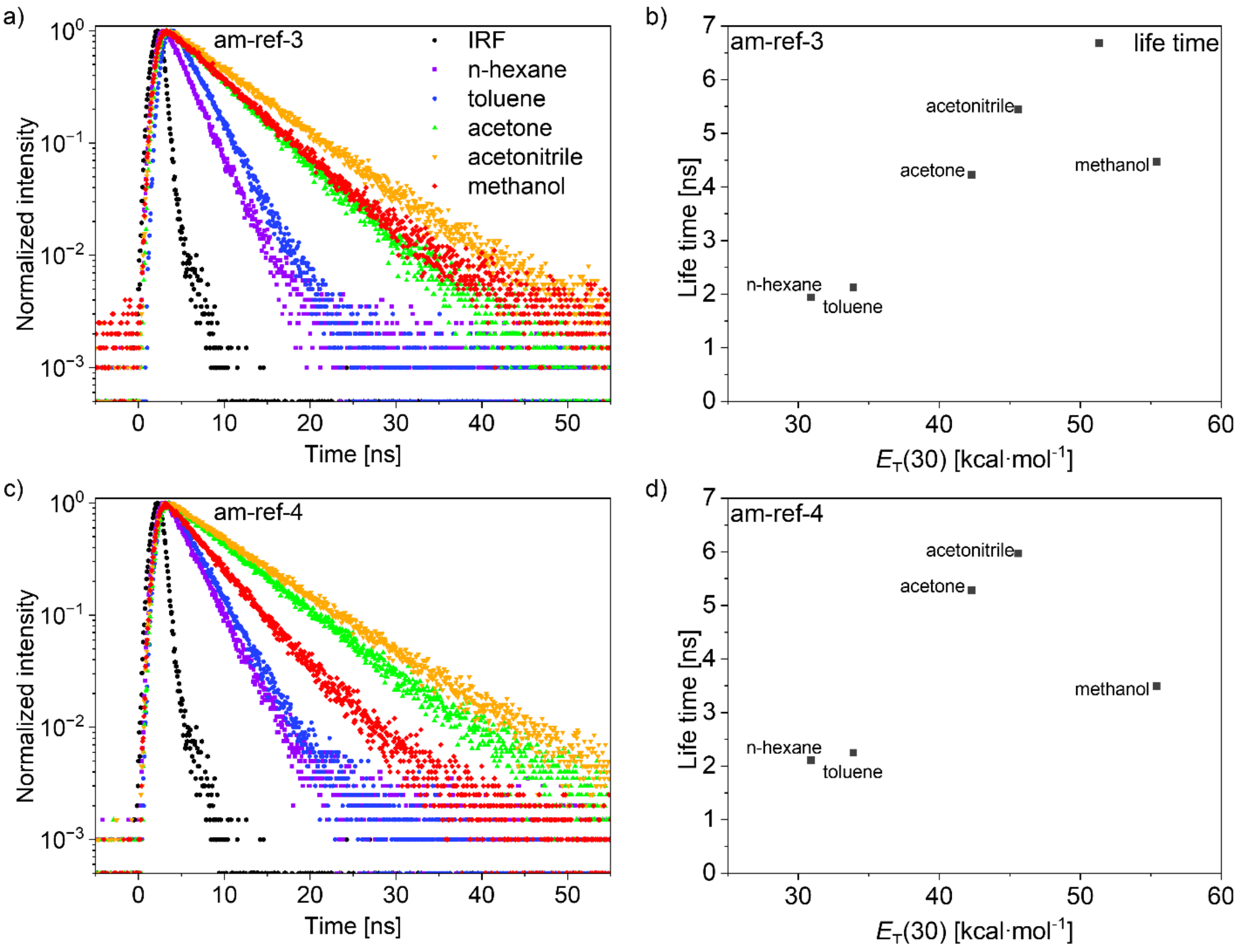

Figure S29 a) and c) TCSPC intensity and b) and d) lifetime dependence on $E_T(30)$ solvents' polarity for reference **amines** solutions.

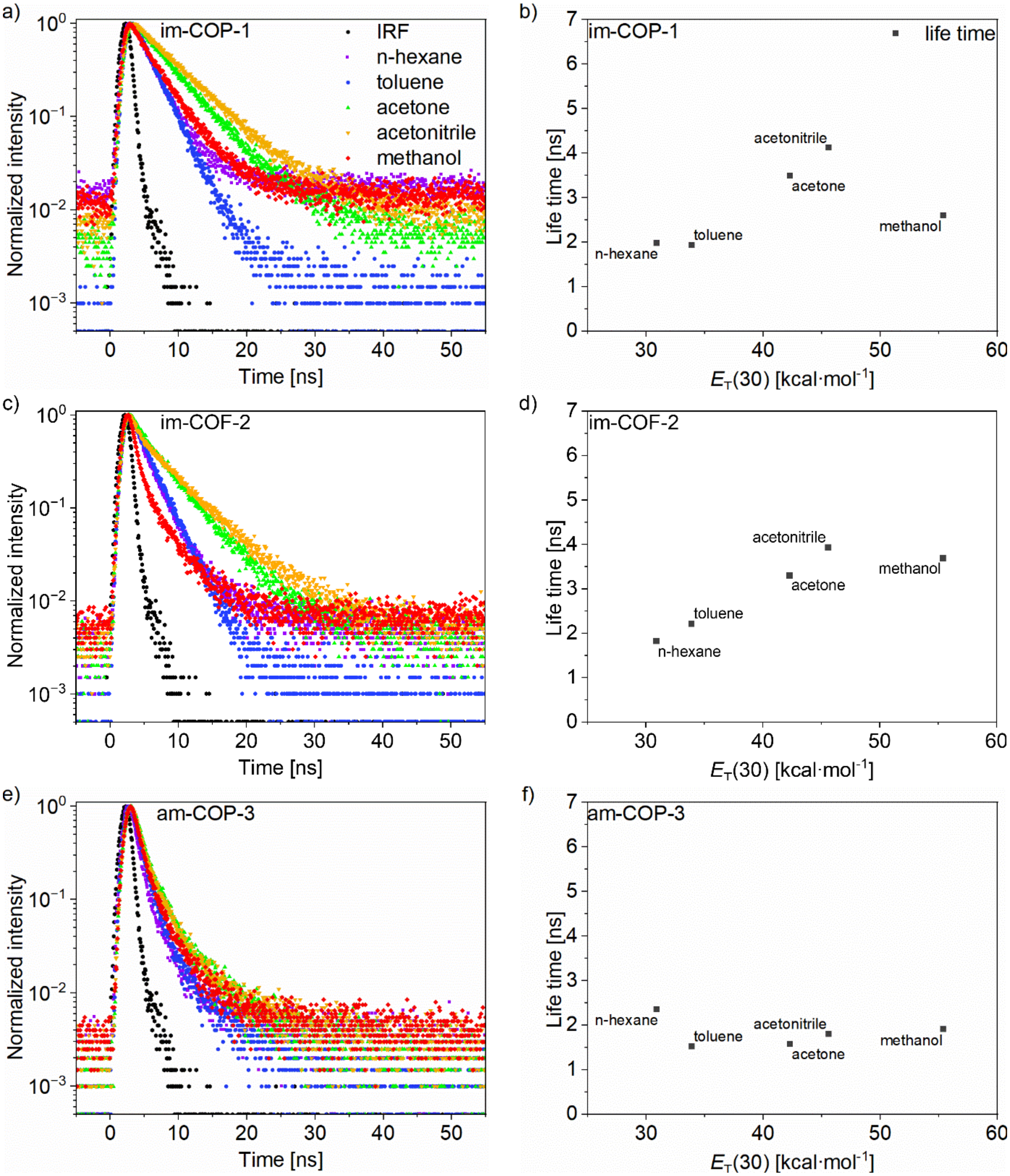

a)
im-COP-1
IRF
n-hexane
toluene
acetone
acetonitrile
methanol
Normalized intensity
Time [ns]
b)
im-COP-1
life time
Life time [ns]
acetonitrile
acetone
toluene
n-hexane
methanol
$E_T(30)$ [kcal·mol^{-1}]
c)
im-COF-2
Normalized intensity
Time [ns]
d)
im-COF-2
Life time [ns]
acetonitrile
methanol
acetone
toluene
n-hexane
$E_T(30)$ [kcal·mol^{-1}]
e)
am-COP-3
Normalized intensity
Time [ns]
f)
am-COP-3
Life time [ns]
n-hexane
acetonitrile
toluene
methanol
acetone
$E_T(30)$ [kcal·mol^{-1}]

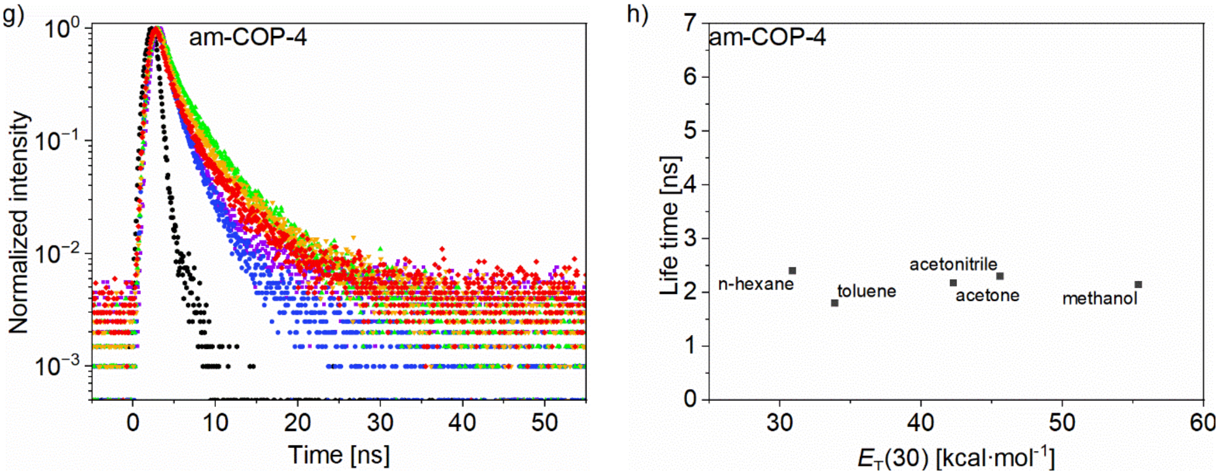

Figure S30 a), c), e), g) TCSPC intensity and b), d), f), h) lifetime dependence on $E_T(30)$ solvents' polarity for **imine** a)-d) and **amine** e)-h) COFs suspensions.

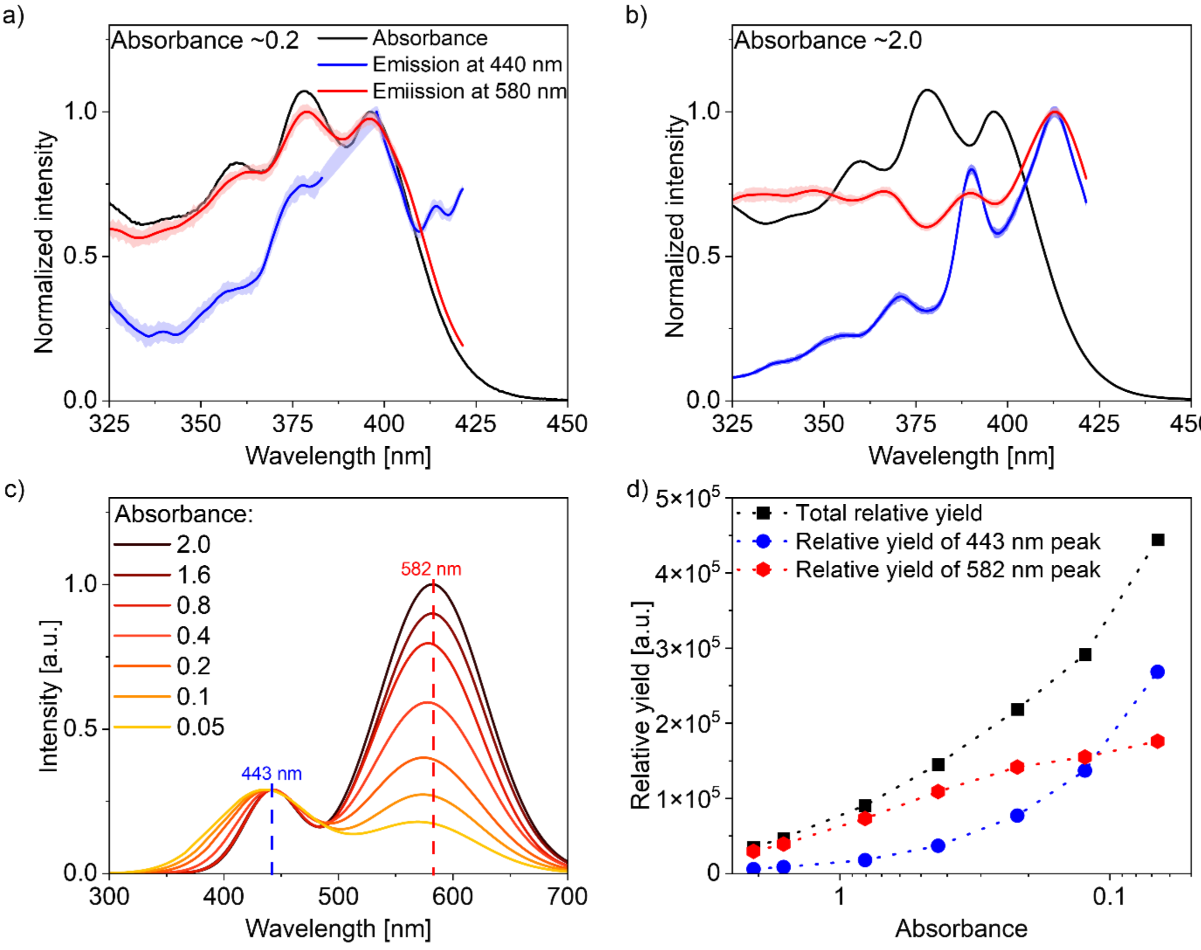

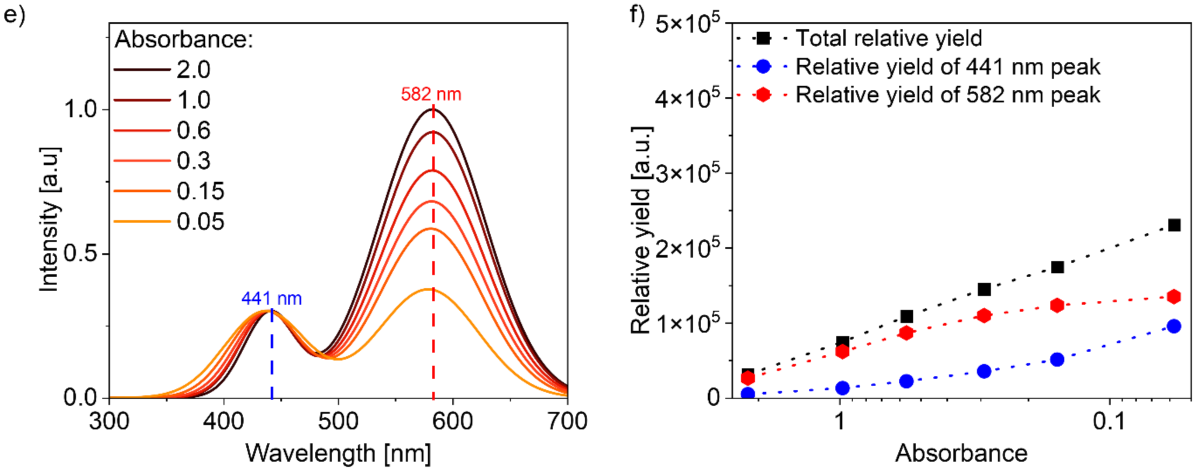

Figure S31 Concentration dependent spectra of **im-ref-2:** a), b) Absorbance and excitation spectra normalized to 3rd peak. c), e) emission spectra for samples with different maximal absorbance (recorded in a range 360–400 nm), and d), f) relative emission yield (integrated emission from a given peak divided by maximal absorbance recorded in a range of 360-400 nm). Data presented at c) – d) graphs are recorded immediately after sample preparation and data at e) and f) are recorded after 7 days of sample maturation in an inert environment (under a dry N_2 atmosphere). The shaded area on graphs a) and b) is STD.

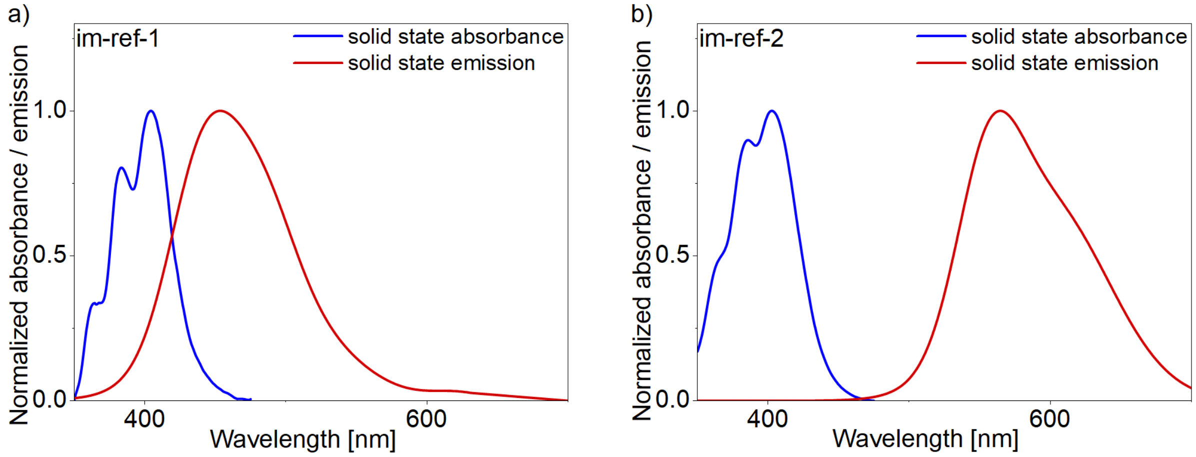

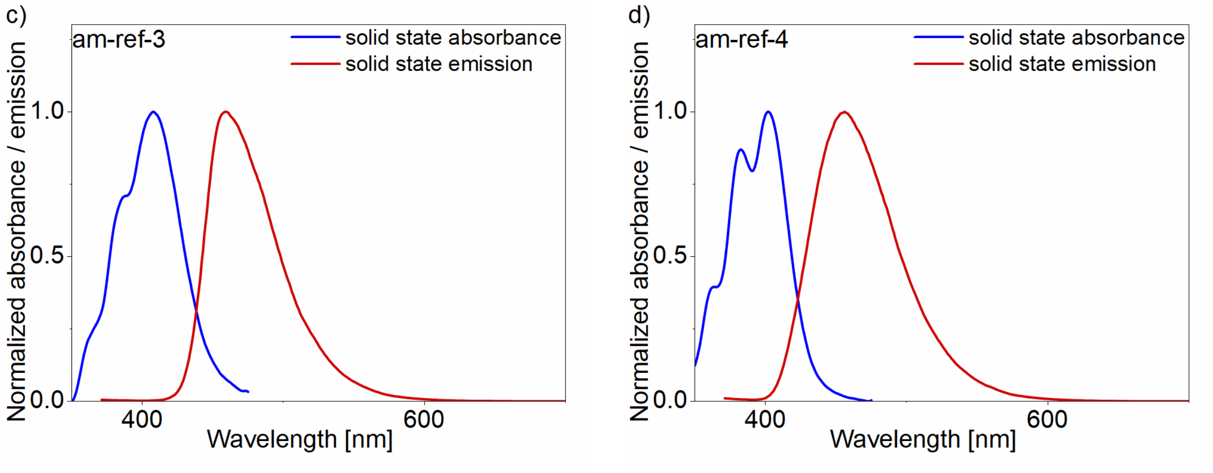

Figure S32 Normalized solid state absorption and emission spectra reference a) and b) imines and c) and d) amines.

12. Chromaticity diagrams

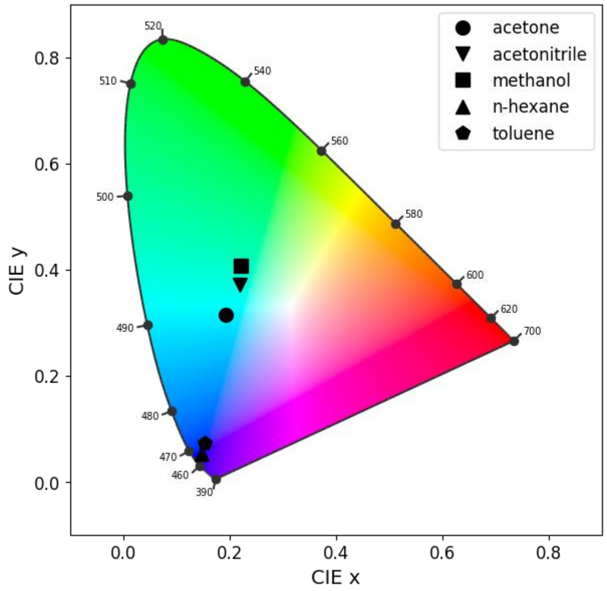

im-ref-1

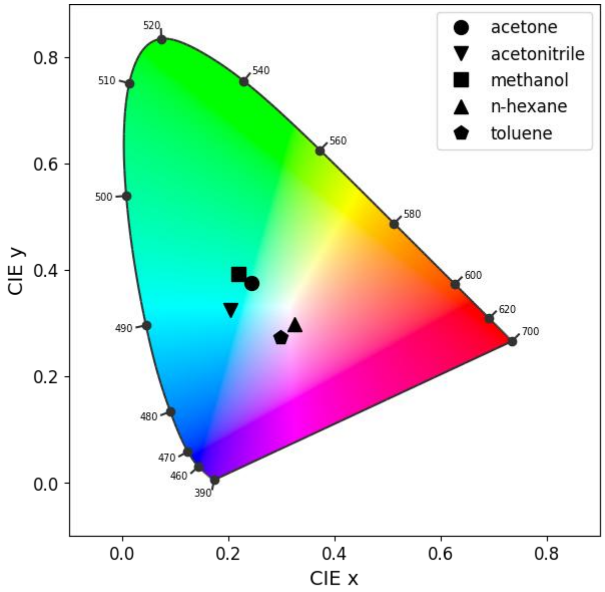

im-ref-2

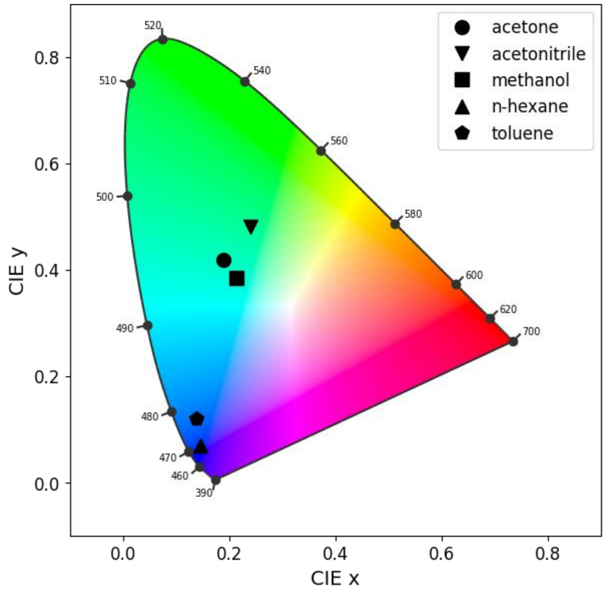

am-ref-3

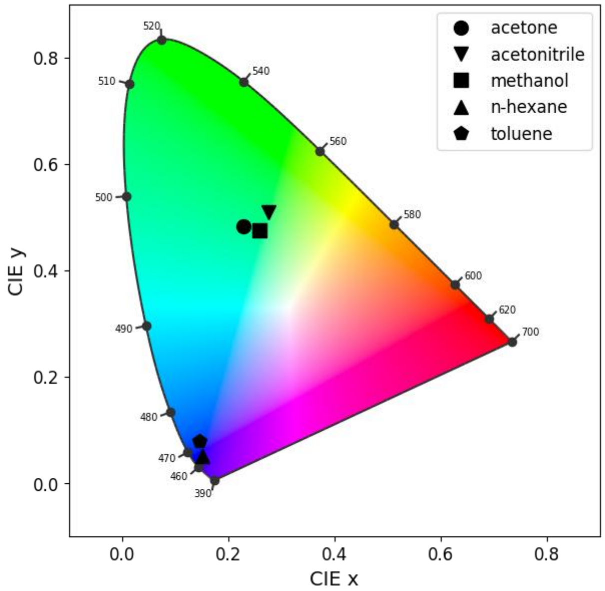

am-ref-4

Figure S33. Chromaticity diagrams calculated for reference imines and amines emission.

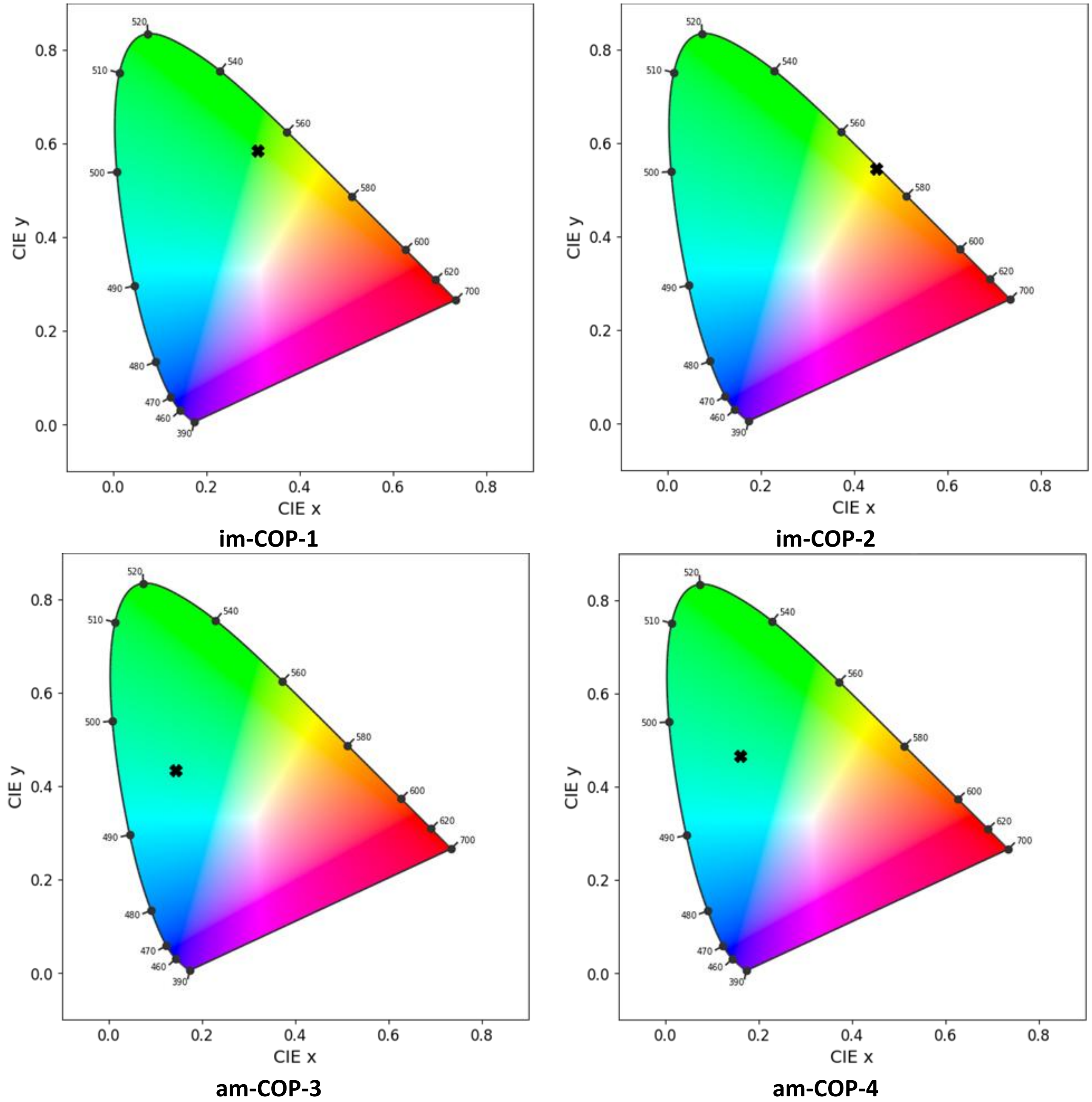

Figure S34. Chromaticity diagrams calculated for COFs emission.

13. Electrochemical measurements

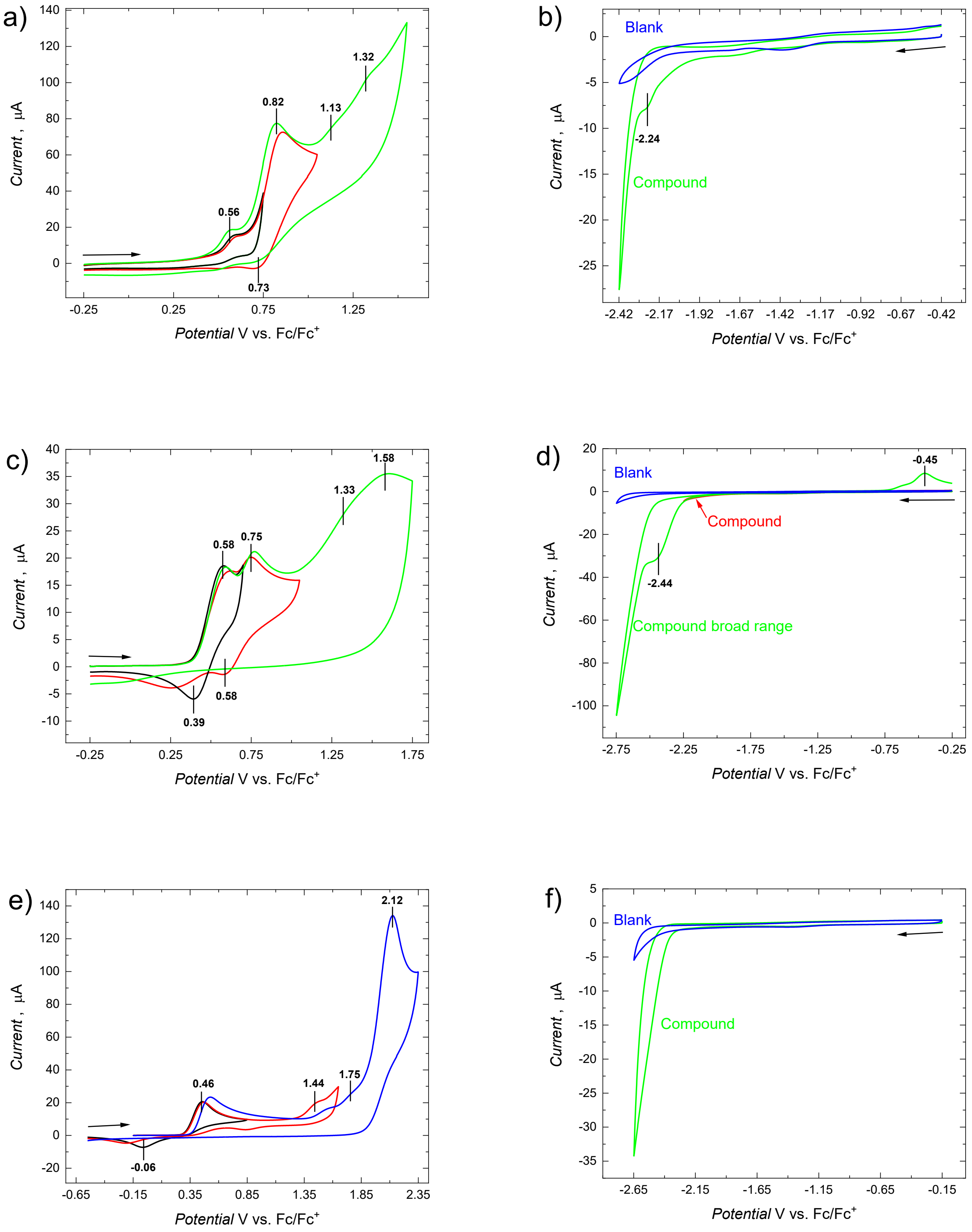

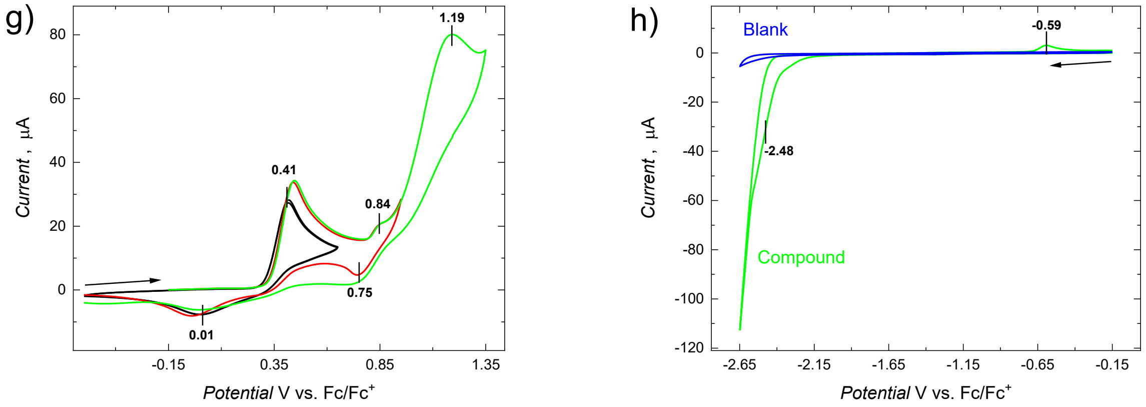

Figure S35. Cyclic voltammetry of a) 1.6 mM **im-ref-1**, c) 1.7 mM **im-ref-2**, e) 1.6 mM **am-ref-3**, and g) 1.7 mM **am-ref-4** in positive potential range, as well as b) 0.5 mM **im-ref-1**, d) 1,7 mM **im-ref-2**, f) 1.6 mM **am-ref-3**, and h) 1.7 mM **am-ref-4** in negative potential range. CVs were recorded on GC working electrode 2 mm in dia. In 0.1 M (TBA)PF_6 solution in CH_2Cl_2 (or acetonitrile/toluene 2:1 *v* : *v* in case of b) with 100 mV/s scan rate.

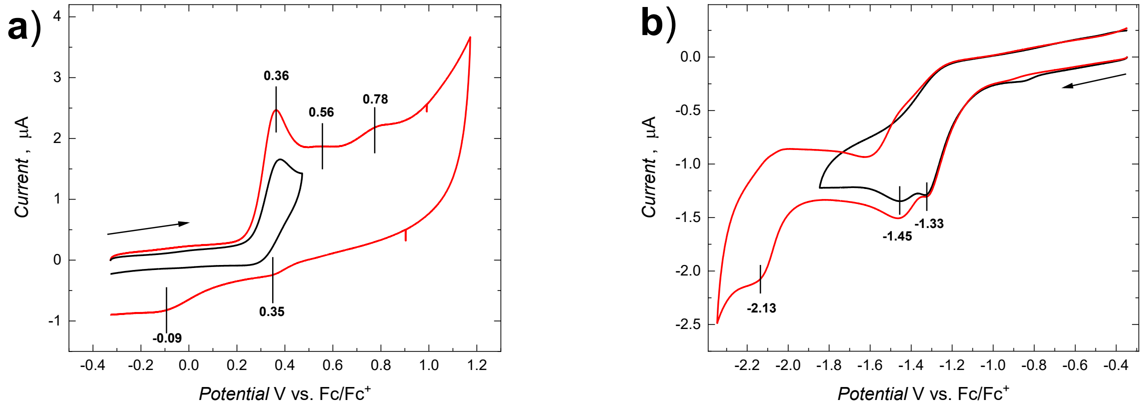

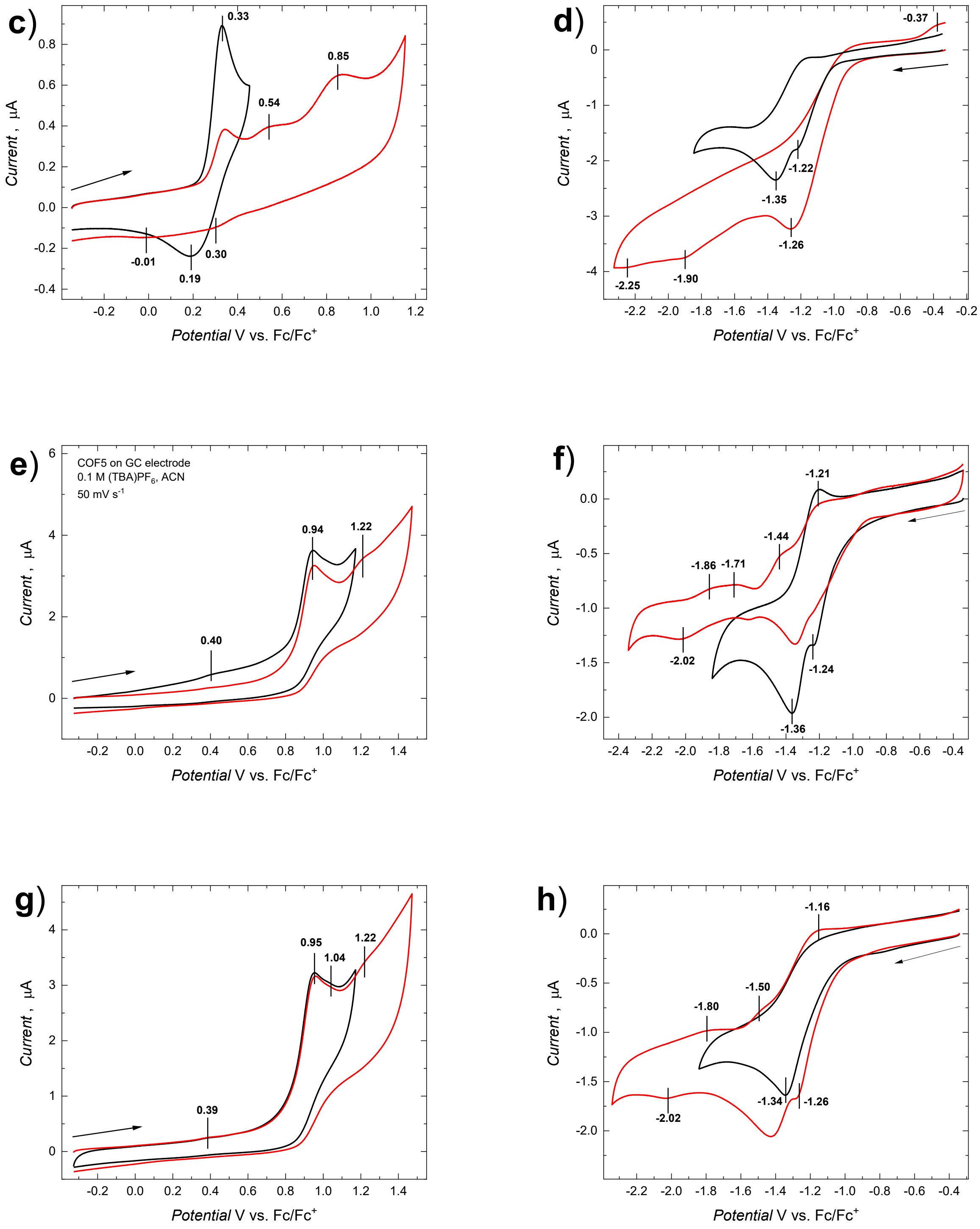

Figure S36. Cyclic voltammetry of solid films of a. b) **im-COP-1**, c, d) **im-COF-2**, e, f) **am-COP-3** and g, h) **am-COP-4** in a, c, e, g) positive and b, d, f, h) negative potential range. Films were deposited on GC working electrode 2 mm in dia. and recorded in 0.1 M (TBA)PF_6 solution in acetonitrile with 50 mV/s scan rate.

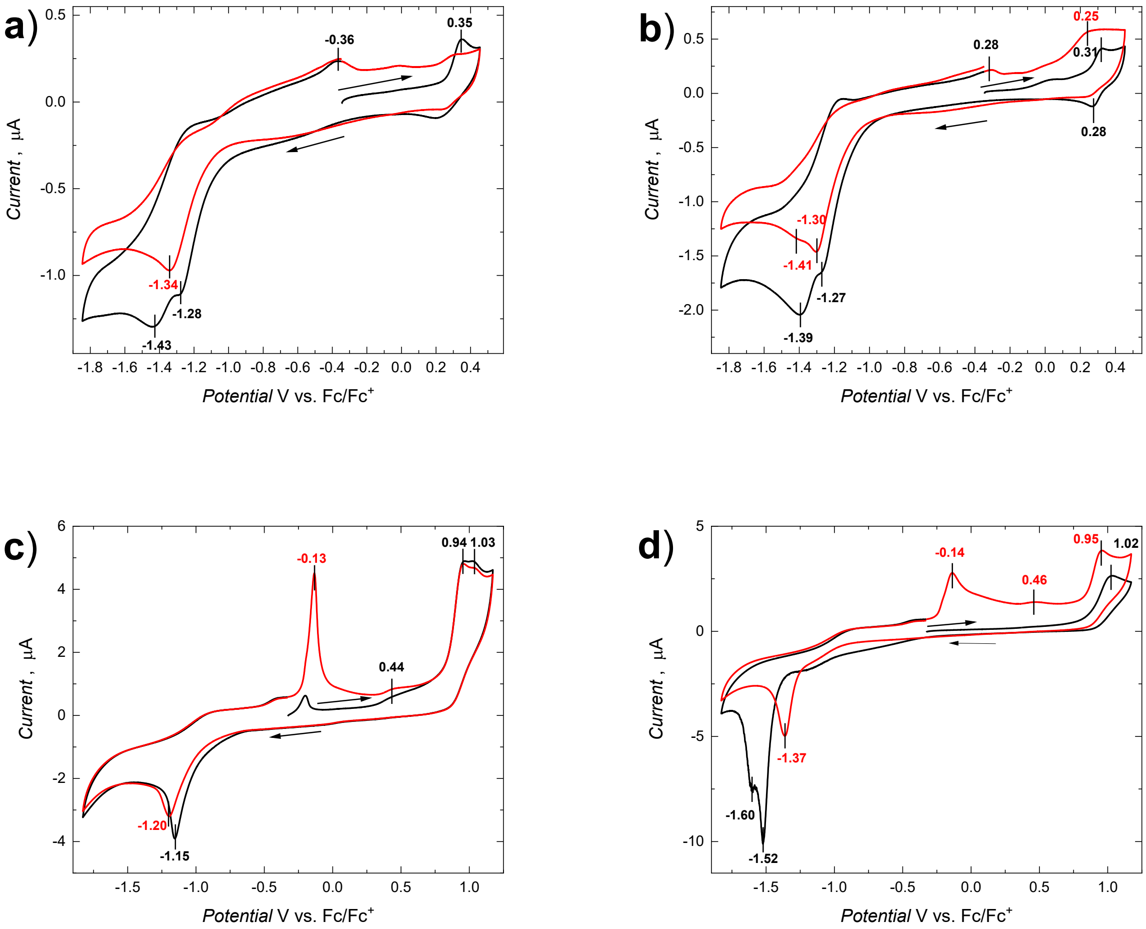

Figure S37. Multicyclic cyclic voltammograms of solid films of a) **im-COP-1**, b), **im-COF-2**, c), **am-COP-3** and d) **am-COP-4** in broader potential range. Films were deposited on GC working electrode 2 mm in dia. and recorded in 0.1 M (TBA)PF_6 solution in acetonitrile with 50 mV/s scan rate.

Table S13. Measured and calculated electrochemical characteristics of reference compounds and COFs

Compound	E_a^1 V vs. Fc/Fc^+	*E*(HOMO) , eV	E_c^1 V vs. Fc/Fc^+	*E*(LUMO) , eV	Electrochemical bandgap, eV
**im-ref-1**	0.55[a]	-5.26	-2.05[a]	-2.66	2.60
**im-ref-2**	0.48[b]	-5.19	-2.25[a]	-2.46	2.73
**am-ref-3**	0.29[a]	-5.00	-2.33[a]	-2.38	2.62
**am-ref-4**	0.29[a]	-5.00	-2.35[a]	-2.36	2.64
**im-COP-1**	0.25[a]	-4.96	-1.87	-2.84	2.12
**im-COF-2**	0.23[a]	-4.94	-1.99	-2.72	2.22
**am-COP-3**	0.82[a]	-5.53	-1.88	-2.83	2.70
**am-COP-4**	0.81[a]	-5.52	-1.90	-2.81	2.71

[a] onset potential

[b] formal potential

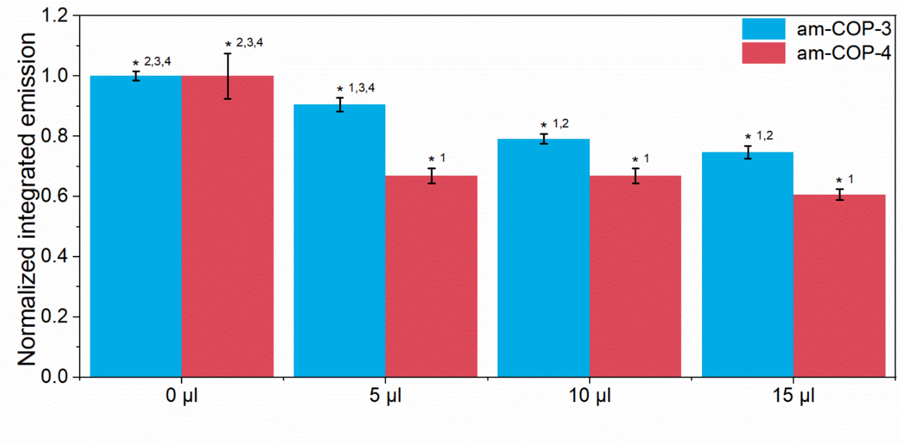

Figure S38. Normalized integrated emission of **am-COP-3** and **am-COP-4** suspensions upon successive additions of water. Suspensions were prepared in 3000 µL of dehydrated acetonitrile with 200 µg of powderized COF. Measurements were performed in an integrating sphere with an excitation wavelength of 380 nm, and the full emission spectra (400-720 nm) were integrated. Asterisks indicate statistical difference ($p < 0.05$, one-way ANOVA) from the column(s) corresponding to the number(s) provided.

14. Literature

[1] V. Petříček, M. Dušek, L. Palatinus, "Crystallographic Computing System JANA2006: General features" *Z. Für Krist. - Cryst. Mater.* **2014**, *229*, 345–352.

[2] M. Thommes, K. Kaneko, A. V. Neimark, J. P. Olivier, F. Rodriguez-Reinoso, J. Rouquerol, K. S. W. Sing, "Physisorption of gases, with special reference to the evaluation of surface area and pore size distribution (IUPAC Technical Report)" *Pure Appl. Chem.* **2015**, *87*, 1051–1069.

[3] J. P. Cerón-Carrasco, D. Jacquemin, C. Laurence, A. Planchat, C. Reichardt, K. Sraïdi, "Solvent polarity scales: determination of new E_T (30) values for 84 organic solvents" *J. Phys. Org. Chem.* **2014**, *27*, 512–518.

[4] M. N. Polyanskiy, "Refractive index database," https://refractiveindex.info. Accessed on 2025-09-15.

[5] J. E. Saunders, C. Sanders, H. Chen, H.-P. Loock, "Refractive indices of common solvents and solutions at 1550 nm" *Appl. Opt.* **2016**, *55*, 947.

[6] C. Würth, M. Grabolle, J. Pauli, M. Spieles, U. Resch-Genger, "Relative and absolute determination of fluorescence quantum yields of transparent samples" *Nat. Protoc.* **2013**, *8*, 1535–1550.

[7] M. Martini, M. Montagna, M. Ou, O. Tillement, S. Roux, P. Perriat, "How to measure quantum yields in scattering media: Application to the quantum yield measurement of fluorescein molecules encapsulated in sub-100 nm silica particles" *J. Appl. Phys.* **2009**, *106*, 094304.

[8] Colour Developers, Colour-Science/Colour, https://github.com/colour-science/colour. Accessed on 2025-09-15

[9] G. Kresse, J. Hafner, "*Ab initio* molecular dynamics for liquid metals" *Phys. Rev. B* **1993**, *47*, 558–561.

[10] G. Kresse, J. Hafner, "*Ab initio* molecular-dynamics simulation of the liquid-metal–amorphous-semiconductor transition in germanium" *Phys. Rev. B* **1994**, *49*, 14251–14269.

[11] G. Kresse, J. Furthmüller, "Efficiency of ab-initio total energy calculations for metals and semiconductors using a plane-wave basis set" *Comput. Mater. Sci.* **1996**, *6*, 15–50.

[12] G. Kresse, J. Furthmüller, "Efficient iterative schemes for *ab initio* total-energy calculations using a plane-wave basis set" *Phys. Rev. B* **1996**, *54*, 11169–11186.

[13] J. P. Perdew, K. Burke, M. Ernzerhof, "Generalized Gradient Approximation Made Simple" *Phys. Rev. Lett.* **1996**, *77*, 3865–3868.

[14] G. Kresse, D. Joubert, "From ultrasoft pseudopotentials to the projector augmented-wave method" *Phys. Rev. B* **1999**, *59*, 1758–1775.

[15] Y.-B. Zhang, H. Furukawa, N. Ko, W. Nie, H. J. Park, S. Okajima, K. E. Cordova, H. Deng, J. Kim, O. M. Yaghi, "Introduction of Functionality, Selection of Topology, and Enhancement of Gas Adsorption in Multivariate Metal–Organic Framework-177" *J. Am. Chem. Soc.* **2015**, *137*, 2641–2650.

[16] N. D. Litvinas, P. S. Fier, J. F. Hartwig, "A General Strategy for the Perfluoroalkylation of Arenes and Arylbromides by Using Arylboronate Esters and [(phen)CuRF]" *Angew. Chem. Int. Ed.* **2012**, *51*, 536–539.

[17] A. Tromelin, P. Demeresman, R. royer, P. Gayral, J. Fourniat, "Synthèse et étude biologique préliminaire de dérives dichloréthylaminés sur l'homocycle de nitro-2 benzofurannes" *Synthèse Étude Biol. Préliminaire Dérives Dichloréthylaminés Sur Homocycle Nitro-2 Benzofurannes* **1986**, *21*, 397–402.

[18] M. Gutsche, J. Podlech, "Synthesis of Octahydroperylene, the Framework of Altertoxin III" *Eur. J. Org. Chem.* **2024**, *27*, e202301053.

[19] T. K. Ronson, W. Meng, J. R. Nitschke, "Design Principles for the Optimization of Guest Binding in Aromatic-Paneled Fe^{II}_4 L_6 Cages" *J. Am. Chem. Soc.* **2017**, *139*, 9698–9707.

[20] J. Li, J. Wang, Z. Wu, S. Tao, D. Jiang, "Ultrafast and Stable Proton Conduction in Polybenzimidazole Covalent Organic Frameworks via Confinement and Activation" *Angew. Chem. Int. Ed.* **2021**, *60*, 12918–12923.

[21] W. A. Braunecker, K. E. Hurst, K. G. Ray, Z. R. Owczarczyk, M. B. Martinez, N. Leick, A. Keuhlen, A. Sellinger, J. C. Johnson, "Phenyl/Perfluorophenyl Stacking Interactions Enhance Structural Order in Two-Dimensional Covalent Organic Frameworks" *Cryst. Growth Des.* **2018**, *18*, 4160–4166.
